# CellRegMap: a statistical framework for mapping context‐specific regulatory variants using scRNA‐seq

**DOI:** 10.15252/msb.202110663

**Published:** 2022-08-16

**Authors:** Anna S E Cuomo, Tobias Heinen, Danai Vagiaki, Danilo Horta, John C Marioni, Oliver Stegle

**Affiliations:** ^1^ European Bioinformatics Institute (EMBL‐EBI) Cambridge UK; ^2^ Wellcome Sanger Institute Cambridge UK; ^3^ Division of Computational Genomics and Systems Genetics German Cancer Research Centre (DKFZ) Heidelberg Germany; ^4^ European Molecular Biology Laboratory (EMBL) Genome Biology Heidelberg Germany; ^5^ Faculty of Mathematics and Computer Science Heidelberg University Heidelberg Germany; ^6^ Faculty of Biosciences Heidelberg University Heidelberg Germany; ^7^ Cancer Research UK Cambridge Institute Cambridge UK; ^8^ Present address: Garvan Institute of Medical Science Sydney NSW Australia

**Keywords:** cell‐type specificity, eQTL, genetic interaction, single‐cell sequencing, Computational Biology

## Abstract

Single‐cell RNA sequencing (scRNA‐seq) enables characterizing the cellular heterogeneity in human tissues. Recent technological advances have enabled the first population‐scale scRNA‐seq studies in hundreds of individuals, allowing to assay genetic effects with single‐cell resolution. However, existing strategies to analyze these data remain based on principles established for the genetic analysis of bulk RNA‐seq. In particular, current methods depend on *a priori* definitions of discrete cell types, and hence cannot assess allelic effects across subtle cell types and cell states. To address this, we propose the *Cell Regulatory Map* (CellRegMap), a statistical framework to test for and quantify genetic effects on gene expression in individual cells. CellRegMap provides a principled approach to identify and characterize genotype–context interactions of known eQTL variants using scRNA‐seq data. This model‐based approach resolves allelic effects across cellular contexts of different granularity, including genetic effects specific to cell subtypes and continuous cell transitions. We validate CellRegMap using simulated data and apply it to previously identified eQTL from two recent studies of differentiating iPSCs, where we uncover hundreds of eQTL displaying heterogeneity of genetic effects across cellular contexts. Finally, we identify fine‐grained genetic regulation in neuronal subtypes for eQTL that are colocalized with human disease variants.

## Graphical abstract

CellRegMap is a statistical framework to identify and characterise genetic effects on gene expression in single cells. The model has enabled the identification of hundreds of context‐specific eQTL, including variants that are colocalized with human disease variants.

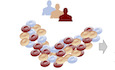

## Introduction

Seminal population‐scale single‐cell RNA sequencing (scRNA‐seq) studies have demonstrated the feasibility to map expression quantitative trait loci (eQTL) using scRNA‐seq as a readout. These studies have replicated eQTL that had previously been discovered using bulk RNA‐seq profiles (Cuomo *et al*, 2020a; Data ref: Cuomo *et al*, 2020b; van der Wijst *et al*, 2018), and more importantly, demonstrated increased resolution by mapping eQTL across individual cell types that are captured by scRNA‐seq (van der Wijst *et al*, 2018; Jerber *et al*, 2021a; Data ref: Jerber *et al*, 2021b; Neavin *et al*, 2021).

Despite the scope of these novel opportunities posed by using scRNA‐seq for genetic mapping, existing strategies for mapping eQTL using single‐cell data remain largely based on principles that were originally devised for bulk RNA‐seq profiling. For example, established “multi‐tissue” eQTL methods (e.g., refs. Ding *et al*, 2010; Petretto *et al*, 2010; Nica *et al*, 2011; Fu *et al*, 2012; Flutre *et al*, 2013; Sul *et al*, 2013; Di Narzo *et al*, 2014; Li *et al*, 2018; Urbut *et al*, 2019) can be adapted to scRNA‐seq, but require discretization of the single‐cell profiles into distinct cell clusters *a priori* to quantify gene expression. Additionally, while these approaches can be used to test for genetic effects in one or more of the defined cell types, they are not designed to model and test for continuous interactions with cell contexts such as “dynamic effects,” where continuous transitions in cell states modulate genetic effect size. Alternatively, “interaction tests” methods do exist and have been applied in the context of eQTL mapping (e.g., refs. Zhernakova *et al*, 2017; van der Wijst *et al*, 2018), yet current workflows remain limited to testing for interactions with one context at a time, and importantly do not effectively account for repeated or related samples. The latter limitation is particularly relevant for single‐cell data, where multiple cells are assayed for each individual. Consequently, these approaches do not fully leverage the resolution provided by single‐cell data, potentially failing to detect changes in allelic regulation across subtle cell subtypes. Discretization of single transcriptome profiles into distinct cell clusters can also be limiting in settings where cell states change in a continuous manner, as for example observed across developmental time courses or cellular differentiation. Additionally, even seemingly well‐defined discrete cell types may share common axes of heterogeneity, e.g., due to cell‐intrinsic factors such as the cell cycle, thus motivating to jointly analyze genetic effects across multiple cell states in order to capture all of these dimensions.

**Figure 1:**
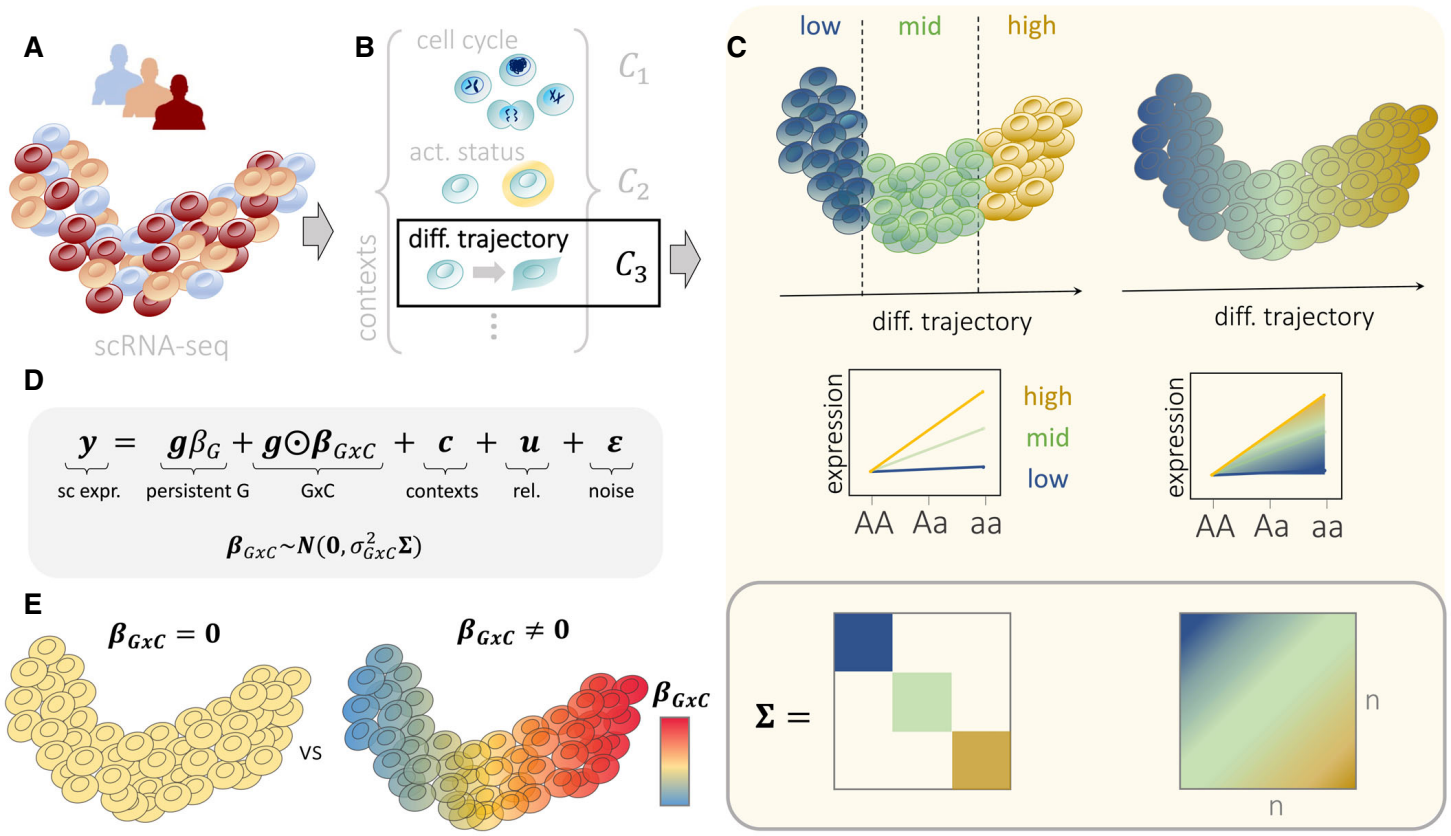
Overview of CellRegMap A, BEstablished workflows based on principal component analysis or factor analysis applied to scRNA‐seq can be used to both estimate cellular manifolds (A) and to uncover individual factors that capture different cellular contexts (B). In addition to capturing major cell types, these factors can also explain subtle subtypes, as well as cell‐type independent variation, such as the cell cycle and other cell‐intrinsic factors. These cellular contexts can represent both discrete and continuous cell‐state transitions, including cellular differentiation.CIllustration of a genotype–context (GxC) interaction where genetic effects are modulated by a cellular differentiation context. Established analysis strategies (left) typically require discretization into discrete cell clusters (here low, mid, high), whereas CellRegMap enables assaying allelic effects as a function of the continuous differentiation context (right). Top panel: cellular manifold with color denoting allelic effects, either estimated in discrete cell populations (left) or in continuous fashion using CellRegMap (right). Middle panel: Alternative representation of allelic effects for different genotype groups, again either considering a discrete (left) or continuous modeling approach (right). Bottom panel: Encoding of discrete cell types (left) and continuous gradients using a cellular context covariance matrix in CellRegMap (right).DThe CellRegMap model can be cast as a linear mixed model, where single‐cell gene expression values of a given gene are modeled as a function of a persistent genetic effect, GxC interactions, additive effects of cellular context, relatedness and residual noise. GxC interactions are modeled by treating allelic effect size estimates in individual cells ($\beta_{\mathrm{GxC}}$) as random variable with prior covariance Σ (C).ECellRegMap allows to test for heterogeneous genetic effects across cells due to GxC at a given locus for a given gene (testing $\beta_{\mathrm{GxC}}=0$ vs. $\beta_{\mathrm{GxC}}\neq 0$). Color denotes the estimated GxC interaction component of genetic effects in individual cells ($\beta_{\mathrm{GxC}}$).

Here, we propose the Cellular Regulatory Map (CellRegMap), a framework for mapping regulatory variants in an unbiased manner across cell types and cell states as obtained from scRNA‐seq profiles. CellRegMap does not depend on any discretization of cells into cell types, nor is it required to annotate specific cell states *a priori*. Instead, the model leverages a multi‐dimensional cell state manifold estimated from single‐cell transcriptome profiles to define *cellular contexts* in a continuous and unbiased manner. CellRegMap then allows to test for and characterize interaction effects between individual genetic variants and cellular context on gene expression traits (Fig 1). The primary use case of CellRegMap is to reanalyze eQTL variants with known additive effects, however the model can in principle also be used for variant discovery (Materials and Methods; Appendix Fig S1). We validate CellRegMap using simulated data, and apply the model to map context‐specific effects of eQTL previously identified in two recent single‐cell genetics studies (Cuomo *et al*, 2020a; Data ref: Cuomo *et al*, 2020b; Jerber *et al*, 2021a; Data ref: Jerber *et al*, 2021b), where we demonstrate increased power to detect genotype–context (GxC) interactions, and we identify regulatory modules of eQTL that are active in the same cellular contexts. Finally, we explore the relevance of cell–context interactions to enhance the characterization of colocalization events with human disease variants.

## Results

CellRegMap generalizes the classical linear interaction model for genotype‐environment interactions (Zhernakova *et al*, 2017; van der Wijst *et al*, 2018) and allows testing for interactions between genotype and a possibly large number of discrete and continuous cellular contexts. Briefly, CellRegMap encodes the cellular context using a covariance matrix that is estimated from the observed scRNA‐seq profiles. This covariance can be derived using existing workflows, including factor analysis (e.g., multi‐omics factor analysis, MOFA; Argelaguet *et al*, 2018) or principal component analysis (PCA; Fig 1A and B). CellRegMap incorporates the estimated cellular context covariance to account for additive contributions from cell context as well as interaction effects with genetic variants using random effect components within the linear mixed model (LMM) framework (Henderson, 1984; Kang *et al*, 2008; Lippert *et al*, 2011; Loh *et al*, 2015) (Fig 1C and D). In addition to a conventional fixed effect test for persistent genetic effects, CellRegMap implements a random effect test to identify GxC interactions due to heterogeneous genetic effects (Materials and Methods; Fig 1D). CellRegMap builds on and extends StructLMM, an LMM‐based method to assess genotype‐environment interactions in population cohorts (Moore *et al*, 2019). In particular, CellRegMap accounts for relatedness between samples using an additional random effect component, thereby appropriately addressing the repeat structure in single‐cell analyses. This is required because typically multiple cells are sampled from the same individuals (Materials and Methods).

More formally, CellRegMap models the single‐cell expression profile of a given gene (across a total of *N* cells from multiple individuals; y) as a sum of a conventional—persistent—genetic effect (G), interactions with cellular context (GxC), additive contributions from cell context (C), a relatedness component (rel.) and residual noise (Fig 1D). GxC interactions are modeled as an element‐wise product between the expanded genotype vector **g** at a given locus and a GxC effect size vector $\beta_{\mathrm{GxC}}={\left[\beta_{\mathrm{Gx}C_{1}},\ldots ,\beta_{\mathrm{Gx}C_{N}}\right]}^{T}$, which correspond to allelic effect sizes in individual cells. This vector follows a multivariate normal distribution, $\beta_{\mathrm{GxC}}∼N\left(0,{\sigma_{\mathrm{GxC}}}^{2}\Sigma \right)$. Depending on the parametrization of the cell–context covariance Σ, CellRegMap can be set up to model interactions with different cellular contexts, including discrete and related cell types, as well as continuous cell‐state transitions (Fig 1C and D; Materials and Methods). The same covariance is also used to account for additive effects of cellular context on expression, i.e.,$\;c∼N\left(0,{\sigma_{C}}^{2}\Sigma \right)$. To account for the repeat structure caused by sampling multiple cells from the same individual, CellRegMap includes an additional relatedness component, which is parametrized as the element‐wise product between a conventional relatedness covariance R (expanded to the level of individual cells) and the cell context covariance, i.e., $u∼N\left(0,{\sigma_{\mathrm{RC}}}^{2}R\;⊙\;\Sigma \right)$ (see Materials and Methods). This component ensures that the model retains calibration when multiple cells are sampled from the same individual. Finally, the model assumes Gaussian distributed and independently and identically distributed residual noise, i.e., $\varepsilon ∼N\left(0,{\sigma_{n}}^{2}I\right)$ (Fig 1D).

We propose a score test to identify gene‐loci pairs with significant GxC effects (testing $\beta_{\mathrm{GxC}}\neq 0$, Fig 1E), which generalizes the approach in ref. (Moore *et al*, 2019). Additionally, CellRegMap can be used to characterize GxC effects of eQTL by estimating the allelic effect for individual cells $\beta_{\mathrm{GxC}}$, which allows us to identify specific cell populations with increased or decreased genetic effects (Fig 1C; Materials and Methods). The model is implemented in efficient open‐source software, which leverages low‐rank representations and factorizations of the resulting total covariance, after marginalizing the random effect components (Materials and Methods). As a result, the computational complexity of CellRegMap scales linearly in the number of cells (Appendix Fig S1; Materials and Methods). The model can be applied efficiently to assess GxC interactions at known eQTL variants. The CellRegMap software also comes with an efficient implementation of a conventional association test that is consistent with the CellRegMap model and hence suitable for single‐cell sequencing data (CellRegMap‐Association, Materials and Methods). This test allows to rapidly screen for loci with association signals that can then be assessed using the CellRegMap interaction model (Appendix Fig S1).

### Model validation using simulated data

**Figure 2:**
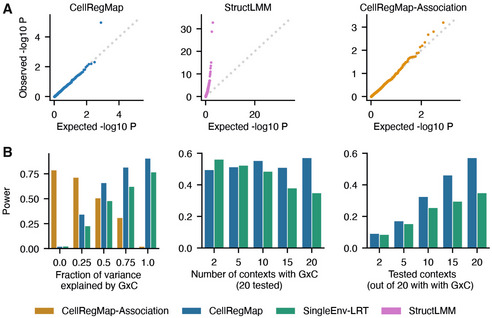
CellRegMap validation using simulated data Test performance for 500 simulated semi‐synthetic eQTL based on real expression profiles and genotypes (Materials and Methods).
ATest calibration under the null hypothesis (without any genetic effects). StructLMM, a model that does not account for the repeat structure in single‐cell sequencing data yields inflated test statistics. *P*‐values from CellRegMap and CellRegMap‐Association, a variant of CellRegMap for detecting persistent genetic effects only (Materials and Methods), follow the expected uniform distribution.BPower at significance level α = 0.01 as a function of the fraction of genetic variance explained by GxC (left), the number of simulated contexts with GxC (middle) and the number of tested contexts (out of 20 all contributing to GxC, right). Compared are CellRegMap, CellRegMap‐Association (where applicable) and a fixed‐effect likelihood‐ratio‐test for single contexts (minimum *P*‐value across all contexts, Bonferroni‐adjusted for the number of tested contexts; Materials and Methods).

**Figure EV1:**
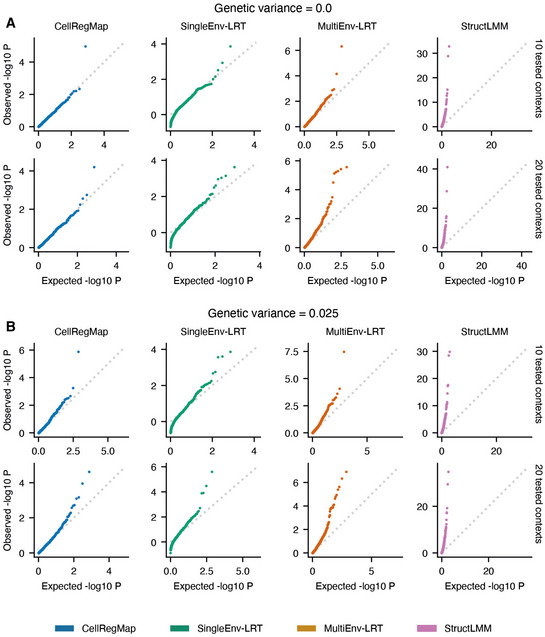
Assessment of statistical calibration based on simulated data Semi‐empirical simulated data as described in Materials and Methods. A, BQQ plots displaying expected versus observed negative log *P*‐values, considering different levels of persistent genetic variance (panel A versus panel B). Rows represent different numbers of tested context variables (10 or 20). All models control for the same number of background contexts as tested (additive effects of environmental context and context‐repeat‐structure interaction). Shown are CellRegMap (blue), a fixed‐effect likelihood‐ratio‐test for single contexts (SingleEnv‐LRT, min. *P*‐value across all contexts, Bonferroni‐adjusted for the number of tested contexts; green), a multicontext fixed‐effect test (MultiEnv‐LRT; orange) and StructLMM (pink; Moore *et al*, 2019). The latter model is related to CellRegMap but does not account for the repeat structure present in single‐cell data. All other models include a random effect component that captures the donor repeat structure across sampled cells. MultiEnv‐LRT and StructLMM do not achieve calibrated test statistics. Note that the QQ plots for CellRegMap, SingleEnv‐LRT and StructLMM from the first row in panel A (genetic variance = 0 and 10 contexts tested) are reused in Fig 2A.

Initially, we considered simulated data to validate the calibration of CellRegMap and to assess statistical power of the model. In particular, we used a semisynthetic simulation procedure, which builds on empirically observed genotypes, gene expression profiles and cellular contexts extracted from real scRNA‐seq data (Data ref: Cuomo *et al*, 2020b; Materials and Methods). We confirmed the statistical calibration of CellRegMap, both when simulating no genetic effects (Figs 2a and EV1) and when simulating from a persistent effect model without GxC interactions (Fig EV1; Materials and Methods). We also compared CellRegMap to StructLMM (Moore *et al*, 2019), a reduced model that does not include the relatedness component, thereby confirming that the relatedness component is required to retain calibrated *P*‐values when multiple cells are assayed from each individual (Figs 2A and B, and EV1). Next, we conducted experiments to assess the statistical power of CellRegMap for identifying loci with simulated GxC effects (Fig 2b; Materials and Methods). For comparison, we also considered a conventional linear interaction test (similar to the approach in Zhernakova *et al*, 2017) that assesses a linear interaction with individual cellular contexts (SingleEnv‐LRT; adjusted for multiple testing across factors using Bonferroni; Materials and Methods), but using otherwise the same random effect components to account for additive effects of context and relatedness employed in CellRegMap (Materials and Methods). Initially, we considered simulated expression profiles with variable fractions of genetic variance explained by GxC. The power of both tests increased as the fraction of the genetic effect explained by GxC increases, noting that CellRegMap was substantially better powered than the SingleEnv‐LRT test. As a second parameter, we varied the number of cellular contexts that are simulated to contribute to GxC (out of 20 included in both tests). The results of this analysis show that CellRegMap outperformed the corresponding SingleEnv‐LRT GxC test when larger numbers of cellular contexts were simulated to contribute to GxC (> 5 contexts). We also varied the number of cellular contexts tested in the model, again finding that CellRegMap offers advantages for larger numbers of contexts.

We extended the benchmark to consider additional comparisons partners. In addition to the CellRegMap interaction test, we assessed the CellRegMap association test (CellRegMap‐Association), which as expected is best powered to identify variants with primarily association signals (Fig 2A). We also considered a fixed effect equivalent of CellRegMap, implemented as a linear interaction test that accounts for multiple contexts at once using a multiple degrees of freedom approach (MultiEnv‐LRT), which however was not calibrated in particular for larger numbers of cellular contexts (Materials and Methods; Fig EV1). Furthermore, we considered discrete cellular contexts (derived using clustering, Materials and Methods) instead of continuous cellular context covariance, which was inferior on both simulated and real data (Appendix Fig S2). Taken together, these results demonstrate power advantages and robustness of CellRegMap, compared with existing methods, particularly when multiple cellular contexts contribute to GxC.

Finally, we used simulated data to assess the impact of expression level and expression variance on the power to detect genuine GxC effects, finding that our power to identify GxC effects is increased for genes with higher overall expression level mean and lower variance (Appendix Fig S3).

### Application to a continuous trajectory of iPS cells differentiating towards definitive endoderm

**Figure 3:**
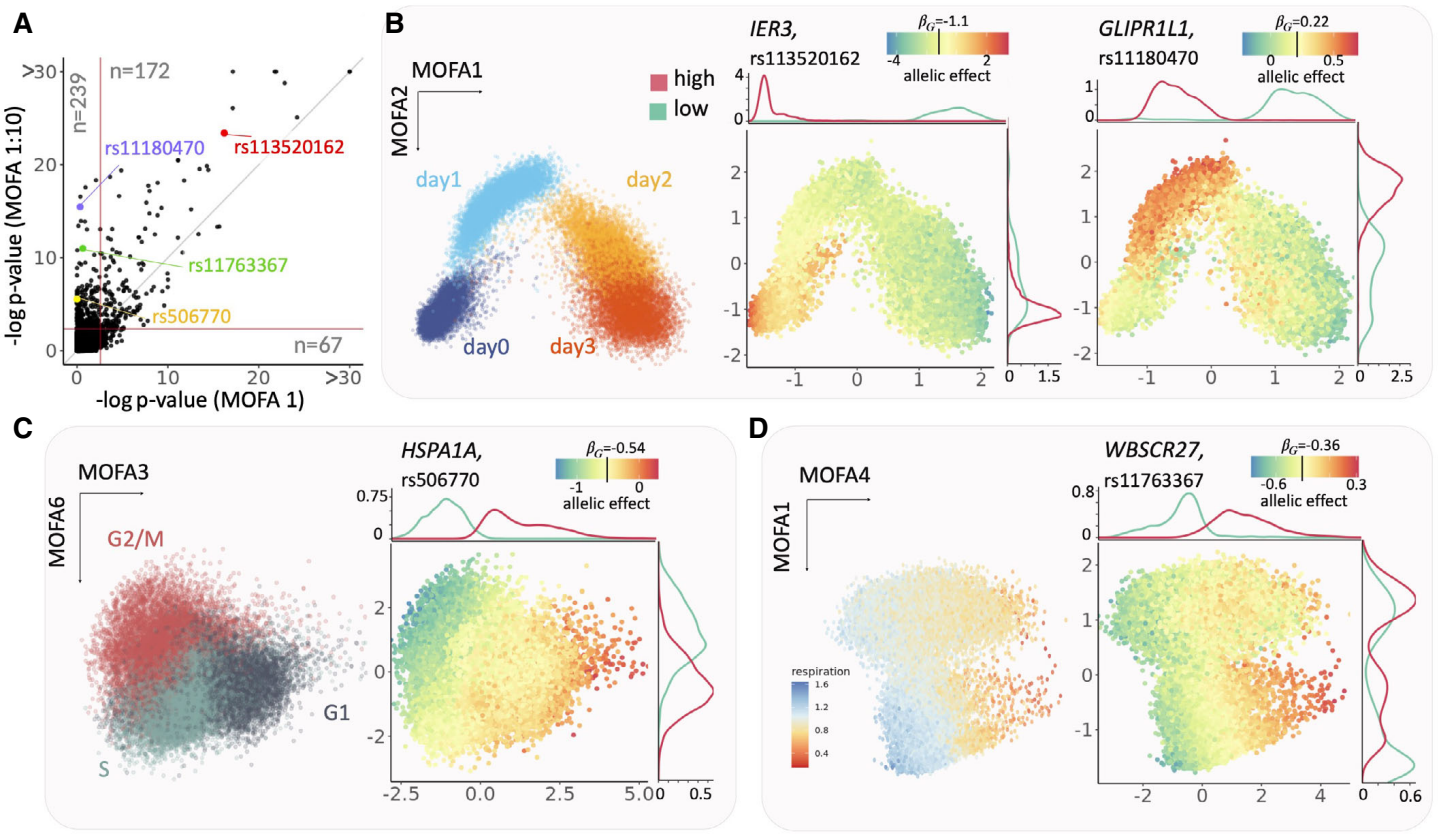
Application of CellRegMap to iPSCs differentiating towards definitive endoderm AComparison of the CellRegMap GxC interaction test, either considering the first MOFA factor to define the cell context covariance (MOFA 1, *x*‐axis) or using the leading 10 cellular factors (MOFA 1:10, *y*‐axis). Shown is a scatter plot of negative log *P*‐values obtained from the respective tests applied to 4,470 eQTL variants and genes. Horizontal and vertical lines denote the FDR < 5% significance threshold (Benjamin‐Hochberg adjusted). Shown in each quadrant is the number of eQTL with evidence for a GxC effect (e.g., 239 GxC effects are exclusively detected by the model that uses 10 MOFA factors; FDR < 5%).B–DRepresentative examples of eQTL with GxC interaction. (B) Left: scatter plot of the first two MOFA factors (capturing cell differentiation as context) with color denoting the time point of collection (days 0, 1, 2 and 3 of endoderm differentiation); middle: identical scatter plot with color encoding the estimated allelic effect for the eQTL variant rs113520162 for the gene *IER3*; right: allelic effect for the eQTL at rs11180470 for the gene *GLIPR1L1*. Shown are total allelic effects ($\beta_{G}+\beta_{\mathrm{GxC}}$) for individual cells. The allelic effect size color bar is centered on the persistent genetic effect ($\beta_{G}$). Panels on the top and right display marginal densities of cells that have either increased (high, red) or decreased (low, cyan) allelic effects (corresponding to the bottom and top 10% quantiles, respectively). Whereas the GxC effect for the eQTL for *IER3* is primarily explained by the first MOFA component, the GxC effect for *GLIPR1L1* is captured by the combination of the first two MOFA factors. (C) Analogous presentation as in (B), displaying a scatter plot between MOFA factors 3 and 6 with cells colored by alternative annotations. Left: inferred cell cycle phase (Materials and Methods); Right: allelic effects for an eQTL at rs506770 for *HSPA1A* (yellow). (D) As in (B, C) scatter plot of MOFA factors 4 and 1. Left: cells colored by cellular respiration (Materials and Methods); Right: allelic effects for the eQTL at rs11763367 for *WBSCR27* (green).

Next, we applied our model to a single‐cell RNA‐seq dataset of differentiating induced pluripotent stem cells (iPSCs) that spans 125 genetically diverse individuals (Cuomo *et al*, 2020a; Data ref: Cuomo *et al*, 2020b). Briefly, a total of ~ 30,000 cells were captured at four time points of iPSC differentiation (day 0: iPSCs, day 1, day 2 and day 3 of differentiation towards definitive endoderm; Fig 3), using the SMART‐Seq2 (Picelli *et al*, 2014) protocol. As expected, cell differentiation is the dominant cellular context in this study, and hence this dataset is an ideal test case to assess the ability of CellRegMap to identify continuous changes of allelic effects across a cellular trajectory.

**Figure EV2:**
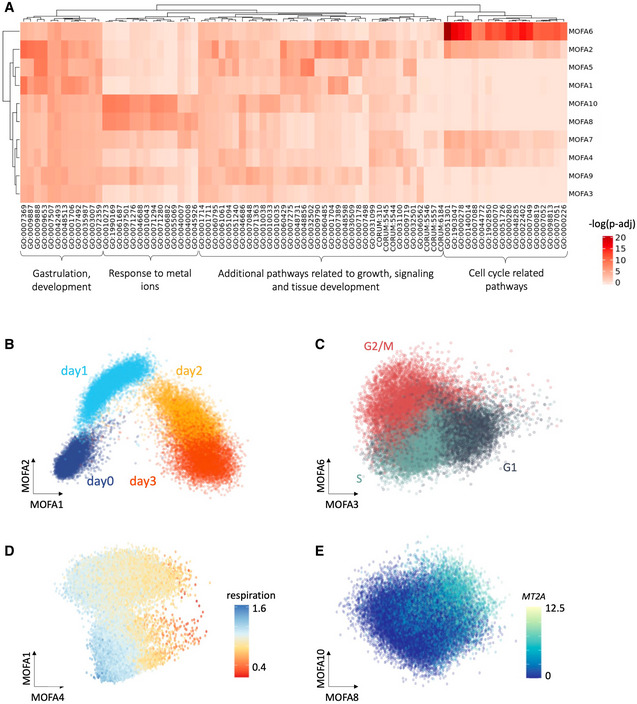
Annotation of the MOFA factors from the endoderm differentiation study Data ref: Cuomo *et al* (2020b). AHeatmap displaying negative log *P*‐values from GO enrichment analyses based on the absolute values of the loadings of individual MOFA factors (Materials and Methods).BScatter plot of MOFA factors 1 & 2, with color corresponding to the time point of collection (day 0,1,2 & 3 of endoderm differentiation).CScatter plot of MOFA factors 3 & 6, colored by estimated cell cycle phase (G1, G2/M, S; estimated using Seurat).DScatter plot of MOFA factors 1 & 4 capturing respiration.EScatter plot of MOFA factors 8 & 10, capturing a signature linked to response to metal ions, colored by expression of gene with top loadings, *MT2A* (Materials and Methods). Note that the scatter plots of MOFA factors in panels (B, C and D) are reused as reference in Fig 3.

We used MOFA (Argelaguet *et al*, 2018) to infer latent factors that explain variation in gene expression in the data, which captured both differences in major cell types across the differentiation trajectory, but also more subtle cell states. For example, the first factor (MOFA 1) primarily explained the differentiation axis, with cells transitioning between a pluripotent state and the definitive endoderm fate. Higher order factors captured other cellular contexts, including cell cycle phase (MOFA 3 and 6), respiration (MOFA 4) and others (Fig EV2; Materials and Methods).

We applied CellRegMap to test for GxC effects at 4,470 eQTL variant/gene pairs that were previously reported in the primary analysis of the dataset using a conventional eQTL mapping workflow that does not account for GxC interactions (Cuomo *et al*, 2020a; Data ref: Cuomo *et al*, 2020b). For comparison, we also considered the CellRegMap Association test to identify eQTL variants within a single end‐to‐end workflow, which yielded a consistent set of variants (Appendix Fig S4). We compared CellRegMap when only using the first MOFA factor to define the cell context covariance, which is similar to the approach taken in the primary analysis (Cuomo *et al*, 2020a; Data ref: Cuomo *et al*, 2020b), to a model that leverages the information contained in the leading 10 MOFA factors. The model with 10 components yielded a substantially larger number GxC effects (322 vs. 183, FDR < 5%; Fig 3A, Table EV1), indicating that despite cell differentiation being the major driver of expression variation, other more subtle cellular states also manifest in GxC interactions on gene expression.

To assess the robustness of the identified GxC effects, we considered alternative latent variable methods to capture cellular contexts, including principal component analysis, which yielded broadly consistent results (Appendix Fig S5; Materials and Methods). As a second technical control, we investigated the extent to which variation in genetic effects due to GxC across the contexts were associated with changes in expression level of the same genes. Reassuringly, the gene expression dynamics and the eQTL dynamics tended to be distinct, demonstrating that gene expression level is not the primary mechanism governing variation in genetic effects (Appendix Fig S6). Finally, we assessed whether variation in gene expression level could result in synthetic interactions, finding that the model retained calibration in such settings (Appendix Fig S7).

**Figure EV3:**
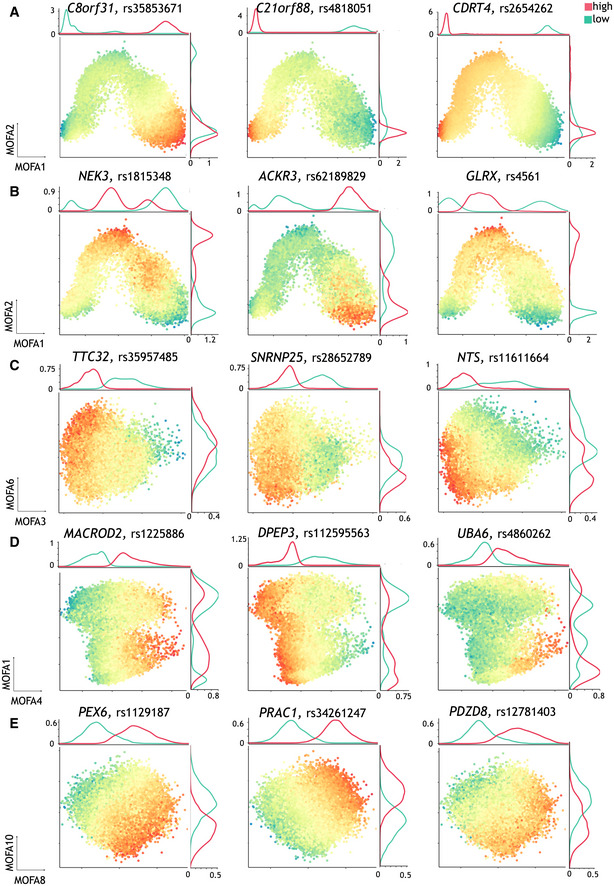
Additional examples of GxC interactions identified in the endoderm differentiation study using CellRegMap Data ref: Cuomo *et al* (2020b). AAnalogous to main text Fig 3B (middle), examples of dynamic eQTL that vary continuously along MOFA 1 only.BSimilar to the main text Fig 3B (right), additional examples of eQTL with GxC effects associated with MOFA factors 1 & 2.CAdditional examples as in the main text to Fig 3C, displaying GxC effects across MOFA factors 3 & 6 (capturing cell cycle).DAdditional examples as in the main text Fig 3C, displaying GxC effects along MOFA factors 4 and 1 (capturing cell respiration).EExamples of eQTL with GxC effects that are associated with cell states captured by MOFA 8 & 10, related to response to metal ions (c.f. Fig EV2).

Next, we set out to characterize specific cellular contexts that are associated with the identified GxC interactions. We used CellRegMap to estimate the GxC allelic effects in each cell, thereby recovering the continuous landscape of the GxC component of genetic effects across the cell–context manifold (Materials and Methods). This analysis identified a range of allelic patterns, including GxC effects that are primarily governed by cellular differentiation but also more complex patterns that involve multiple cellular contexts and higher‐order cellular factors. For example, the eQTL variant rs113520162 for *IER3* had a GxC effect that reflects variation across cell differentiation explained by the first MOFA component (Fig 3B, middle). Other eQTL, such as rs11180470 for *GLIPR1L1*, had GxC effects that were associated with two MOFA factors (Fig 3B, right). More generally, we observed that higher order MOFA components capture changes in cellular contexts beyond cellular differentiation, including the cell cycle (Fig 3C), cellular respiration (Fig 3D), and others (Fig EV3). Collectively these results illustrate how CellRegMap can be used to uncover different cellular contexts that manifest in GxC interactions.

### Application to iPSC‐derived dopaminergic neurons

Next, we applied CellRegMap to a single‐cell dataset of 215 iPS cell lines that were assayed at three stages of differentiation towards dopaminergic neurons (Jerber *et al*, 2021a; Data ref: Jerber *et al*, 2021b) (11, 30 and 52 days of differentiation) using the 10X Genomics technology (3′ kit; Zheng *et al*, 2017), as well as a stress condition at the most differentiated time point. These data feature prominent discrete cell states rather than continuous changes, thus providing a complementary use case.

**Figure 4:**
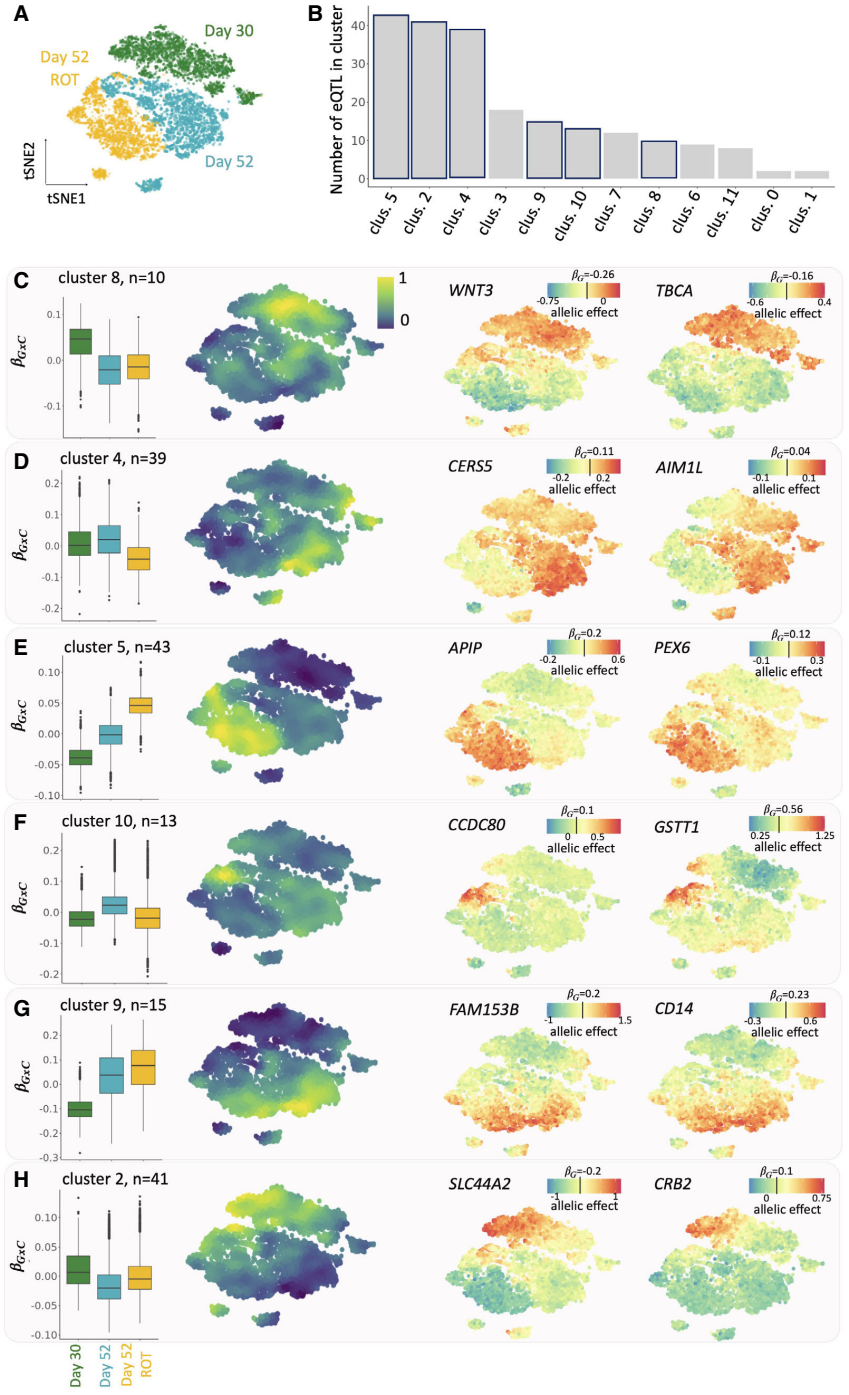
CellRegMap identifies fine‐grained regulatory modules in dopaminergic neurons AOverview of the cell subpopulations: tSNE plot of 8,648 pseudocells (Materials and Methods), highlighting three major populations of dopaminergic neurons: young neurons (day 30 of iPSC differentiation), more mature neurons (day 52) and rotenone‐treated day 52 dopaminergic neurons (day 52 ROT).B–HResults from clustering of GxC allelic effect size estimates of individual cells for different genes. (B) Barplots indicating the number of eQTL with GxC effects assigned to each of 12 clusters. Highlighted are the six representative clusters that are displayed in subsequent panels. (C–H) For each of 6 representative clusters, from left to right: box plot of the distribution of the relative GxC effect sizes estimates for cells in each of the three major cell populations (as in A), manifold of consensus relative GxC effect sizes estimates for each cluster (A); example eQTL with allelic effect size estimates across the cell manifold as in a with color denoting total allelic effects ($\beta_{G}+\beta_{\mathrm{GxC}}$); the color bar is centered on the persistent genetic effect size estimate for each eQTL ($\beta_{G}$). For box plots, central bands represent median values, upper and lower box limits represent the 25% and 75% quantiles, and whiskers correspond to minimum and maximum values.

To assess whether CellRegMap can identify GxC effects associated with finer grained neuronal subtypes, we considered 147,801 cells that were annotated as dopaminergic neurons in the primary analysis of this dataset (based on marker genes; Jerber *et al*, 2021a; Data ref: Jerber *et al*, 2021b). This selection included cells collected at three of the four time points and conditions: young neurons (at day 30 of iPSC differentiation), mature neurons (day 52) and mature neurons followed by rotenone treatment (day 52 ROT). A t‐SNE embedding of these cells identified discrete cell populations that reflect the combination of differentiation stage and stimulus (Fig 4A). Our hypothesis is that while it is expected that regulatory variants can be specific to these major sub populations, there could also be GxC effects that are more granular due to cellular contexts that capture subpopulations within these clusters, or that capture shared cellular contexts that are present across these clusters. To mitigate the sparsity of 10X sequencing data compared with SMART‐Seq2, we aggregated the read count information into pseudocells (similar to approaches described in refs. (Baran *et al*, 2019; DeTomaso *et al*, 2019); resulting in 17 cells on average, 8,648 pseudocells in total, Appendix Fig S8; Materials and Methods). We again considered the leading 10 MOFA components to define the cell context covariance for analysis using CellRegMap.

We tested for GxC effects at 1,374 SNP‐gene pairs identified as eQTL in at least one of the three discrete cell populations in the primary analysis of the data (Jerber *et al*, 2021a; Data ref: Jerber *et al*, 2021b) (FDR < 5%; Materials and Methods). This identified 213 eQTL with evidence for GxC interactions (FDR < 5%, Materials and Methods, Table EV2). We also considered the impact of considering a discrete cell–context covariance, either using the three cell populations described in the original paper (Jerber *et al*, 2021a; Data ref: Jerber *et al*, 2021b) or using clustering to derive discrete contexts (Materials and Methods), revealing that in all cases a discrete cell–context covariance resulted in a much smaller number of discoveries (Appendix Fig S2D–F).

**Figure EV4:**
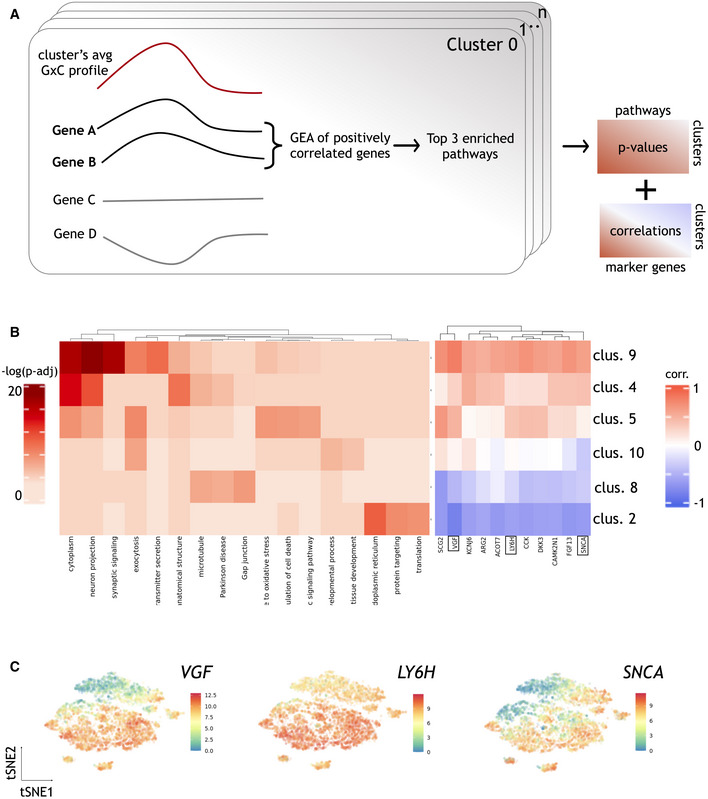
Annotation of GxC clusters based on gene set enrichment in the neuronal differentiation data Data ref: Jerber *et al* (2021b). AIllustration of the clustering approach to identify patterns of GxC interactions. Briefly, for each cluster we considered genes whose single‐cell profiles were positively correlated with the average GxC allelic effect profile. Genes with positive correlation (Pearson's correlation > 0.4) were considered for gene enrichment analysis using gprofiler, and up to 3 top significant (adjusted *P*‐values < 0.05) terms were considered per cluster (Materials and Methods).BLeft: Enrichment results for the 6 clusters in Fig 4C–H. Shown is a heatmap of adjusted negative log *P*‐values of enrichment results obtained by gprofiler. Right: Correlation coefficients between the GxC allelic effect profile and expression level for selected literature‐curated dopaminergic neuron markers. For each cluster, the correlation coefficient between the expression level of the respective marker gene and the aggregate cluster allelic effect size profile is shown.CExpression profiles across pseudocells for three selected neuronal marker genes highlighted in the right panel of (B). tSNE plots, colored by expression level of the three genes.

Next, for each of the 213 eQTL with a significant GxC effect, we again estimated GxC allelic effects in individual cells. To identify general patterns of genetic regulation, we adapted a clustering approach originally designed for spatial transcriptomics data to group eQTL based on their allelic effect patterns across the cell context manifold (implemented in SpatialDE (Svensson *et al*, 2018; Fig EV4A)). This identified 17 clusters of eQTL with distinct GxC effect profiles (Fig 4B). We annotated individual clusters by examining the subpopulation of cells with the largest absolute GxC effects. Briefly, for each cluster we ranked genes by the correlation between their single‐cell expression profiles and the pattern of absolute GxC allelic effects. Based on this gene ranking, we then assessed enrichments of known pathways (over‐representation analysis using a hypergeometric test and annotations from GO, Reactome, KEGG, HPO and others), as well as using literature‐curated marker gene sets of dopaminergic neurons (see Materials and Methods for details, Fig EV4B).

Some of the clusters primarily captured genetic effects that were specific to the three major cell populations. For example, cluster 8 corresponded to eQTL that are primarily active in the day 30 population, cluster 4 eQTL are primarily active in day 52 cells, and cluster 5 captures effects specific to the rotenone‐treated day 52 cell population (Fig 4C–E). Gene enrichment analysis of these clusters yielded processes that are consistent with the expected function of the corresponding cell populations, such as response to oxidative stress (GO: 006979) for cluster 5 (Materials and Methods; Fig EV4).

Beyond these expected patterns of GxC effects, other eQTL had interaction effects that were explained by clusters that exhibit continuous changes of allelic effects across developmental time, or that are specific to more fine‐grained sub populations (Fig 4F–H). For example, cluster 10 captured eQTL that are active in common subpopulation of day 52 treated and untreated cells (Fig 4F). Functional enrichment analysis linked this cluster to processes related to exocytosis and neurotransmitter transport through synapsis, suggesting an association with neurons that are actively transmitting cell–cell information (Fig EV4). Clusters 2 and 9 exhibit GxC effects with opposing directions, with cluster 2 being associated with increased genetic effects and cluster 9 with decreased effects. Cluster 9 eQTL show increasing absolute effect sizes in more mature neurons, regardless of the stimulation status. Enrichment of this cluster highlights neuronal‐specific features such as synaptic signaling (GO:0099536; Figs 4G and EV4). Cluster 2, on the other hand, is specific to a subpopulation of day 30 cells (Fig 4H) that corresponds to less mature dopaminergic neurons, as evident by continuous gradients of canonical dopaminergic neuronal markers (Fig EV4; Materials and Methods).

We also considered to what extent estimated allelic effects in single cells could be used to identify opposite effects, that is eQTL variants with opposite signs of effects for different cellular contexts. Globally, we identified 45 GxC effects (21%) with putatively opposing effects (defined as at least 25% of cells having opposite sign in the estimated GxC portion of genetic effects; $\beta_{\mathrm{GxC}}$). We also reassessed these effects using conventional eQTL mapping by defining pseudo‐bulk expression profiles in the bottom and top 20% quantiles of GxC allelic effects, which confirmed 72% of these putative opposite effects (Materials and Methods; Appendix Fig S9).

**Figure 5:**
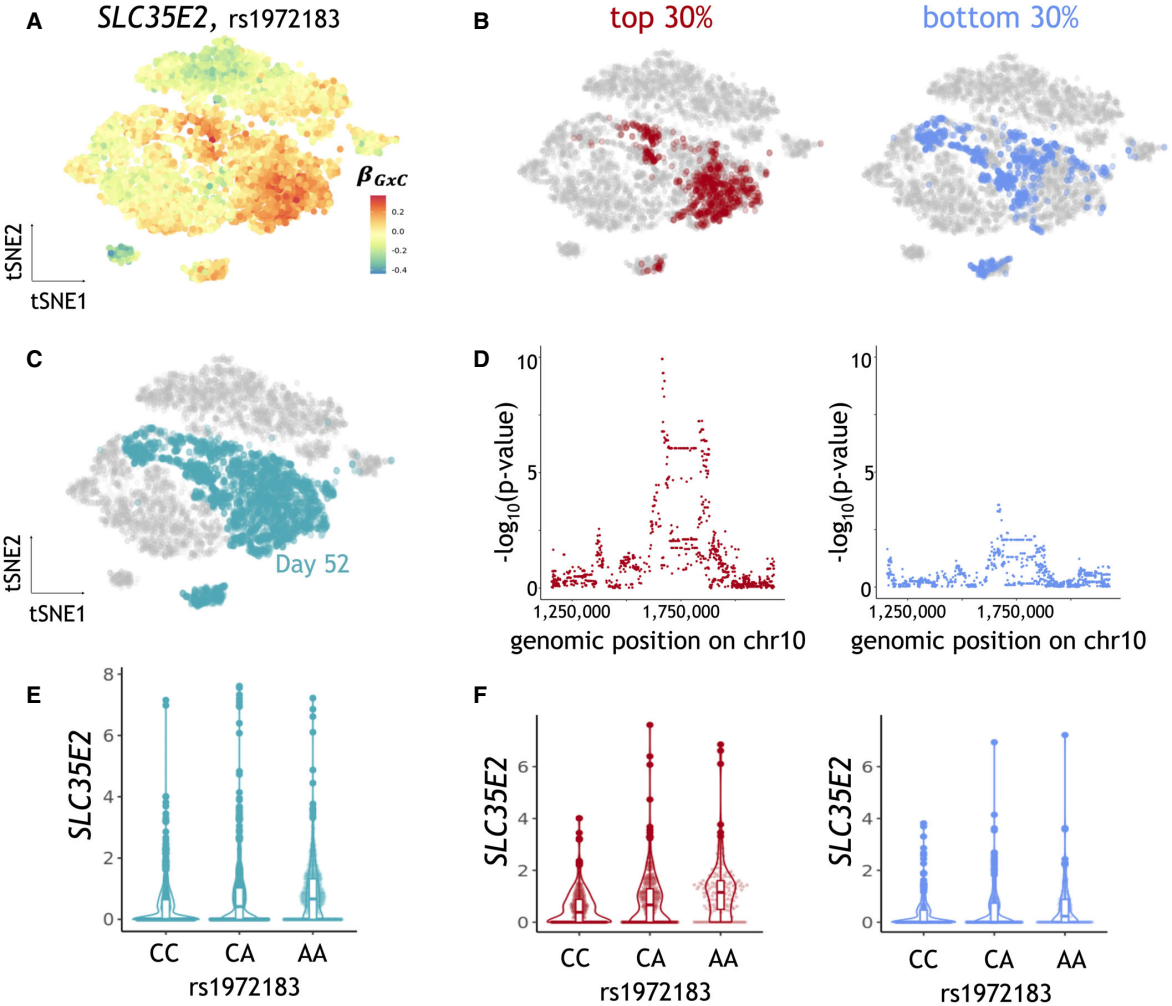
CellRegMap allows to pinpoint subpopulations of cells linked to human disease variants AAllelic effect size estimates for the rs1972183 on *SLC35E2*. Shown is a scatter plot of tSNE coordinates with color denoting estimated GxC allelic effects ($\beta_{\mathrm{GxC}}$).B–F
*SLC35E2*‐eQTL results obtained from a conventional eQTL mapping workflow (Materials and Methods), using the CellRegMap output to select alternative cell populations to estimate expression phenotypes. (B, C) tSNE plots as in a, with color indicating alternative selected subpopulations of day 52 untreated cells. (B) Top and bottom 30% quantiles of day 52 untreated cells ranked by the absolute GxC allelic effect. (C) Day 52 untreated cells. (D) Manhattan plots displaying negative log *P*‐values from a conventional eQTL workflow when using the subpopulations as in b to estimate expression phenotypes. Shown are negative log *P*‐values (*y*‐axis) as a function of the genomic position of common variants (*x*‐axis). (E, F) Violone plots displaying effect size estimates on *SLC35E2* (*y*‐axis) stratified by genotype at the lead variant rs1972183 (*x*‐axis), either considering all cells for pseudo bulk expression estimation (E) or (F) considering the subpopulations as in (B, D).

**Figure EV5:**
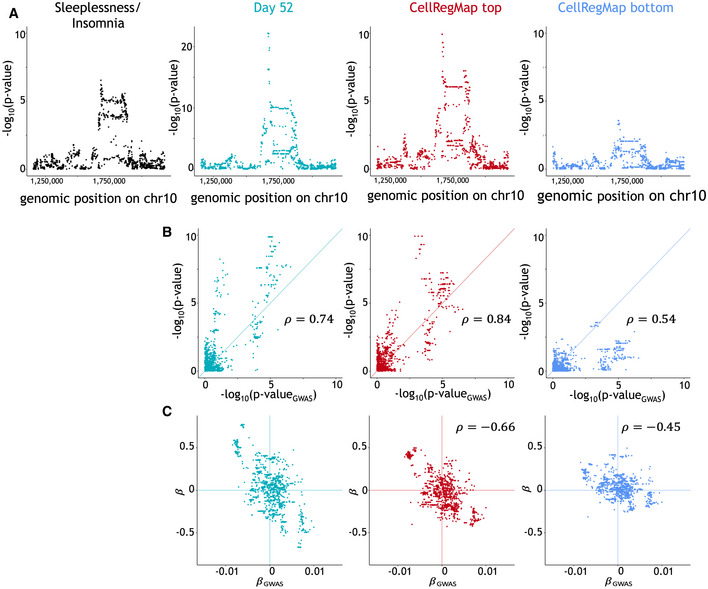
CellRegMap allows to characterize human disease variants colocalized with eQTL in dopaminergic neurons GxC profile at rs1972183 for *SLC35E2* identifies cellular population linked to GWAS variant for insomnia/sleeplessness. Data ref: Jerber *et al* (2021b), when considering cells identified as dopaminergic neurons only (Materials and Methods). Columns represent all cells at day 52 (aqua), cell population that CellRegMap identifies to have strongest effects (red, top 30% quantile from $\beta_{\mathrm{GxC}}$) and weakest (blue, bottom 30% quantile).
AManhattan plots for the relevant genomic region. From left to right: GWAS for Sleeplessness Insomnia, eQTL using aggregate expression estimates across all day 52 (untreated), eQTL Manhattan plot when considering day 52 cells in the top quantile, eQTL Manhattan plot when considering day 52 cells in the bottom quantile. Note that the latter two Manhattan plots for the top and bottom quantiles using CellRegMap are reused in Fig 5D.B, CComparison between GWAS signal and eQTL signal, considering alternative traits based on the cell populations as in (A). (B) Scatter plots of negative log *P*‐values from GWAS (*x*‐axis) versus eQTL (*y*‐axis). (C) As in (B), displaying effect size estimates.

Finally, we considered a subset of 94 eQTL with evidence for statistical co‐localization with GWAS hits for neuronal and human disease traits (Jerber *et al*, 2021a; Data ref: Jerber *et al*, 2021b) (Materials and Methods). Out of these, 14 eQTL had significant GxC interactions. For example, the eQTL variant rs1972183 for *SLC35E2* has a GxC effect explained by cluster 4 and is colocalized with a GWAS variant for sleeplessness and insomnia in the subpopulation of day 52 untreated cells (Fig 5A–C). CellRegMap allowed for pinpointing a specific sub‐population within this cluster with elevated allelic effect sizes (Appendix Fig S10A). We again considered the allelic GxC effect estimates in single cells to stratify cells into the top and bottom 30% quantiles of absolute genetic effects, and conducted conventional *cis* eQTL mapping using the corresponding pseudo bulk expression profiles (Cuomo *et al*, 2021). This analysis confirmed the expected difference in eQTL effect sizes (Fig 5D–F) and, notably, the trait associated with the top quantile of allelic effects yielded higher evidence for co‐localization with the disease GWAS signal (Fig EV5).

## Discussion

Here, we presented the cellular regulatory map (CellRegMap), a linear mixed model for the identification and characterization of context‐specific eQTL that is applicable to cellular states derived from scRNA‐seq. Critically, CellRegMap overcomes the need to define cellular contexts *a priori* (Fig 1) and instead uses cell manifolds derived from single‐cell transcriptome profiles to estimate cellular contexts in an unbiased manner to then test for interaction effects.

Conceptually, CellRegMap is related to and builds on StructLMM, a model that was originally designed to identify genotype‐environment interactions in population cohorts (Moore *et al*, 2019). CellRegMap adapts these concepts to single‐cell genomics, by including an additional relatedness component in the model to account for dependencies across cells that are assayed from the same individual. CellRegMap retains calibrated test statistics (Figs 2A and B, and EV1) and enjoys power benefits compared with conventional fixed‐effect interaction tests (Fig 2C–E). Additionally, we complement our framework with a fast genetic association test designed specifically for single‐cell sequencing data (CellRegMap‐Association), allowing to efficiently generate sets of candidate eQTL to be tested for GxC.

To illustrate the model, we applied CellRegMap to a single‐cell dataset of iPS cells from 125 individuals across differentiation towards a definitive endoderm fate (Cuomo *et al*, 2020a; Data ref: Cuomo *et al*, 2020b). The main source of variation in this dataset is a continuous differentiation signal, which manifests in dynamic eQTL across differentiation. Notably, we also identify eQTL associated with other dimensions of transcriptome variation, including factors associated with cell‐cycle phase or respiration (Fig 3). As a second‐use case, we applied CellRegMap to scRNA‐seq data from iPSCs across differentiation towards a dopaminergic neuronal fate (Jerber *et al*, 2021a; Data ref: Jerber *et al*, 2021b). Our analysis demonstrated that cell‐type specific eQTL are not only observed for major subpopulations linked to known cell types, but instead a substantial number is driven by other more subtle variations in cellular context (Fig 4).

An important insight from both use cases is that continuous and subtle allelic regulation, which manifests in GxC in specific subpopulations, is common. Even in cell populations that seemingly correspond to well‐defined cell types, CellRegMap identified heterogeneity in genetic effects that manifests in GxC interactions. These interactions are particularly relevant if they are linked to eQTL with evidence for colocalization with human disease variants. We illustrated this for one disease‐linked GxC effect, where CellRegMap allowed to pinpoint the specific subpopulation of cells that is primarily responsible for this eQTL signal. Notably, this step does not only enhance the interpretation of most relevant cell populations but can also yielded more fine‐grained *cis* eQTL signals for mapping variants.

Although we demonstrated that CellRegMap is broadly applicable to different datasets and scRNA‐seq technologies, the model is not free of limitations. At present, CellRegMap is primarily designed as a tool to annotate known eQTL variants rather than facilitating variant discovery. This is analogous to the two‐stage strategy for mapping of genotype‐environment interactions at known GWAS loci in population cohorts. Such procedures build on the assumption that the persistent genetic effect signal is sufficiently strong to enable discovery. The CellRegMap‐Association test implemented as part of the software can be used to define an end‐to‐end workflow in the cases where eQTL are not known *a priori* (Materials and Methods, Appendix Fig S11). Future extensions of CellRegMap could enable the discovery of eQTL variants while accounting for GxC. A second limitation of the model is that it currently requires appropriate processing steps (e.g., variance stabilization and quantile‐normalization; Materials and Methods) to provide cell‐level or pseudo‐cell expression estimates that approximately follow a Gaussian distribution. Although our results indicate that this approximation is acceptable in practice and retains statistical calibration (Fig EV1), explicit modeling of count data could provide additional power benefits, in particular in the regime of lowly expressed genes. Similarly, standardization of the cell contexts (e.g., MOFA factors) is recommended for robust results, but may in some cases fail to fully capture the underlying structure of the cellular landscape (for example, in the case of outlier cells). Finally, as datasets grow in size, future developments on the scalability may be warranted. While CellRegMap scales linearly in the number of cells already, the computations required to account for the relatedness component could be prohibitive when analyzing very large datasets from thousands of individuals. Datasets of this magnitude will become available through large data‐integration efforts, for example via federated analysis envisioned in the single‐cell eQTLGen consortium (van der Wijst *et al*, 2020).

## Materials and Methods

### Reagents and Tools table

**Table:** 

Reagent/Resource	Reference or Source	Identifier or Catalog Number
**Software**		
Scanpy 1.8.2	https://scanpy.readthedocs.io	
ZINB‐WaVe 1.18.0	https://bioconductor.org/packages/release/bioc/html/zinbwave.html	
scvi‐tools 0.14.5	https://scvi‐tools.org	
LDlinkR 1.2.1	https://cran.r‐project.org/web/packages/LDlinkR/index.html	
CellRegMap 0.0.3	https://github.com/limix/CellRegMap	
MOFA 1.2.0	https://github.com/bioFAM/MOFA	

### Methods and Protocols

#### The cellular regulatory map model

CellRegMap builds on and extends the structured linear mixed model (StructLMM; Moore *et al*, 2019), which has recently been proposed to test for genotype‐environment interactions on physiological traits in population cohorts. CellRegMap extends this model to test for interactions between genotype and cellular context on gene expression using single‐cell RNA‐seq as readout.

The model can be cast as:

$$y=g\beta_{G}+g\;⊙\;\beta_{\mathrm{GxC}}+u+c+e,$$

where

y denotes the log‐transformed single‐cell expression for a given gene,
g is the SNP genotype,
$\beta_{G}$ is the persistent genetic effect,
$\beta_{\mathrm{GxC}}$ $∼N\left(0,\sigma_{\mathrm{GxC}}^{2}\Sigma \right)$ is the cell‐specific GxC effect,
$u∼N\left(0,\sigma_{\mathrm{RC}}^{2}\;R\;⊙\;\Sigma \right)$ accounts for repeat samples,
$c∼N\left(0,\sigma_{C}^{2}\Sigma \right)$ accounts for effects of cell context and
$e∼N\left(0,\sigma_{n}^{2}I\right)$ is the noise term,

and⊙denotes the element‐wise Hadamard product.

##### Relation to StructLMM

CellRegMap extends StructLMM, which can be cast as

$$y=g\beta_{G}+g\;⊙\;\beta_{\mathrm{GxC}}+c+e,$$

by introducing an additional random effect component that accounts for relatedness or sample repeat structure (u). First, we note that the phenotype (y) now represents single‐cell resolved expression data, so samples are expression levels in cells, not individuals. This introduces additional structure in the data, as typically multiple cells are sampled from the same individual. To account for this repeat structure, an additional random effect component is included in the model. This additional random effect component accounts for relatedness or the repeat structure, which is parameterized as a product kernel between relatedness (R) and the environmental covariance (Σ). Here, R denotes the relatedness matrix of individuals expanded to all cells based on the known assignment of cells to individuals and the covariance Σ again denotes the cell‐level environmental context. Notably, this parametrization extends the classical LMM, which would exclusively consider a relatedness component R. One way to interpret this covariance is to account for polygenic interactions between environment and relatedness, which has previously been considered to estimate the GxE component of heritability (Heckerman *et al*, 2016). We note that the introduction of this additional covariance term, R⊙Σ is critical for retaining calibration (Figs 2 and EV1).

##### Construction of the cellular context covariance and processing guidelines

Typically, we define the cellular context covariance using a linear covariance function of a matrix of environmental contexts C, i.e., $\Sigma =CC^{T}$. In practice, we consider as cellular contexts axes of variation in the dataset (for example, captured by principal components or MOFA (Argelaguet *et al*, 2018) factors), appropriately standardized (mean = 0, standard deviation = 1) and build $\Sigma =CC^{T}$ accordingly. Depending on the type and structure of cellular contexts, Σ can simply separate cells into groups, and appear as a block diagonal or capture continuous transitions (Fig 1C). In principle, CellRegMap can also be used in conjunction with other parameterizations of the cell context covariance.

##### The CellRegMap tests and downstream analysis

###### Interaction (GxC) test

The main test implemented in CellRegMap allows users to test for GxC effects for a given eQTL (or gene‐SNP pair). We refer to this as “CellRegMap Interaction test,” or simply “CellRegMap.” The test consists in comparing the following models under the null (H0) and alternative (H1) hypotheses, i.e., we are testing whether $\beta_{\mathrm{GxC}}\neq 0$ (or more accurately, $\sigma_{\mathrm{GxC}}^{2}>0$):

$y=g\beta_{G}+u+c+e$

$y=g\beta_{G}+g\;⊙\;\beta_{\mathrm{GxC}}+u+c+e.$

In order to evaluate the significant contribution of GxC effect, we use a score test, similar to strategies adopted in StructLMM (Moore *et al*, 2019), which in turn uses the approach described in Sequence Kernel Association Test (SKAT; Wu *et al*, 2011). For more details on the implementation, we refer the reader to our Appendix Supplementary Methods.

###### Association test

In addition to the GxC interaction test, it is also possible, within the CellRegMap framework, to test for persistent genetic effects, while appropriately accounting for cell context and multiple cells per donor in the background. CellRegMap‐association essentially draws from the background of the full CellRegMap model and compares the following two models (i.e., testing $\beta_{G}\neq 0$):

y=u+c+e,

and

$y=g\beta_{G}+u+c+e.$

We note that this model is similar to a conventional LMM, bar the specific of the relatedness component ($u∼N(0,\sigma_{R}^{2}R$)). To evaluate statistical significance in this model, we employ a likelihood ratio test (LRT), as implemented within the LIMIX (preprint: Lippert *et al*, 2015) software, which in turn adopts fast LMM testing as first proposed in Lippert *et al* (2011) (Appendix Supplementary Methods).

In cases where eQTL are not already known for a given dataset, the association test can be run first in discovery mode, and all SNP‐gene pairs passing a significance threshold (e.g., we recommend FDR < 20%) can be then tested for GxC effects using the CellRegMap interaction test described above.

###### Single‐cell GxC effect size estimation

Finally, within the CellRegMap framework it is possible to estimate allelic effects in individual cells (thus estimating $\beta_{\mathrm{GxC}}$) for each gene‐SNP pair tested. These estimates can be obtained from posterior predictions of the model, which is analogous to the best linear unbiased predictor (BLUP) in a classical LMM (Henderson, 1984) (Appendix Supplementary Methods).

#### Simulation strategy

Synthetic data for 500 gene‐SNP pairs were generated using empirically observed data extracted from (Cuomo *et al*, 2020a; Data ref: Cuomo *et al*, 2020b), including genotypes (50 individuals), background gene expression profiles (100 cells per individual) and cellular contexts derived from scRNA‐seq data (MOFA factors). We primarily focused on simulating continuous effects by constructing a cell covariance matrix from the observed MOFA factors. As part of the power assessment, we additionally considered discrete contexts (see below). SNPs and genes were sampled uniformly from different chromosomes, thereby avoiding the possibility of confounding the simulated eQTL with existing *cis* eQTL in these data. Synthetic expression counts with GxC effects were simulated for individual cells using a conventional linear interaction model with Poisson noise model:

$$y∼\text{Poisson}\left(\lambda \right),\lambda =\exp\left(y_{\text{base}}+\sum_{i=1}^{k}g\;⊙\;c_{i}{\left(\beta_{\mathrm{GxC}}\right)}_{i}+g\beta_{G}\right),$$

where

$y_{\text{base}}$ is the log‐transformed observed (background) gene expression vector for a given gene in the reference dataset,
g is the SNP genotype vector from the reference dataset,
$c_{i}$ denotes the *i*‐th context variable (MOFA factor),
${\left(\beta_{\mathrm{GxC}}\right)}_{i}∼N\left(0,{\sigma_{G}}^{2}\rho_{\mathrm{GxC}}\right)$ is the interaction effect size for context *i*,
$\beta_{G}∼N\left(0,{\sigma_{G}}^{2}\left(1-\rho_{\mathrm{GxC}}\right)\right)$ is the effect size of the persistent genetic effect and
${\sigma_{G}}^{2}$ denotes the total genetic variance and $\rho_{\mathrm{GxC}}$ is the fraction of genetic variance explained by GxC.

Notably, possible confounding factors such as read count distribution (dropout, overdispersion), batch effects or context‐specific expression variation present in the observed expression counts $y_{\text{base}}$ do not need to be simulated using a parametric or model‐based approach.

##### Calibration

We assessed the statistical calibration of the proposed tests, CellRegMap and CellRegMap‐Association, as well as three alternative models (Figs 2 and EV1):

- **StructLMM** (Moore *et al*, 2019), a linear interaction model for capturing genotype–environment interactions (GxE). This model is conceptually similar to CellRegMap but does not account for the repeat structure present in population‐scale single‐cell measurements.
- **SingleEnv‐LRT**, a fixed‐effect version of CellRegMap, where we test for GxC interactions with individual context dimensions using a likelihood ratio test and report the minimum *P*‐value across all contexts (Bonferroni‐adjusted for the number of contexts).
- **MultiEnv‐LRT**, a fixed‐effect version of CellRegMap with a multiple‐degree‐of‐freedom likelihood ratio test for GxC effects.

Both SingleEnv‐LRT and MultiEnv‐LRT share the same null model as CellRegMap. Data were simulated assuming only persistent ($\rho_{\mathrm{GxC}}=0,{\sigma_{G}}^{2}=0.025)$ or no genetic effects (${\sigma_{G}}^{2}=0.025)$ and testing for GxC effects using either 10 or 20 contexts (MOFA factors). As shown before (Moore *et al*, 2019), MultiEnv‐LRT does not retain calibration for larger numbers of context variables and was therefore excluded from other simulation experiments.

##### Power

Statistical power for CellRegMap, CellRegMap‐Association and SingleEnv‐LRT was evaluated in three different settings (all simulations assume ${\sigma_{G}}^{2}=0.025$, Fig 2):

- Varying $\rho_{\mathrm{GxC}}$, the fraction of genetic variance explained by GxC (0, 0.25, 0.5, 0.75, 1.0) for 10 tested and simulated contexts.
- Varying the number of simulated contexts with GxC (2, 5, 10, 15, 20), for 20 tested contexts (only CellRegMap and SingleEnv‐LRT).
- Varying the number of tested contexts (2, 5, 10, 15, 20) when simulating 20 contexts with GxC (only CellRegMap and SingleEnv‐LRT).

Additionally, we assessed the relationship between statistical power (estimated *P*‐values) and background mean gene expression and variance, as well as minor variant allele frequency (10 tested and simulated contexts, $\rho_{\mathrm{GxC}}=0.5$, Appendix Fig S3). For the same simulated data, we also compared power to detect GxC effects when defining discrete (one‐hot‐encoded) contexts, using either experimental annotations (Cuomo *et al*, 2020a; Data ref: Cuomo *et al*, 2020b) (day of sample collection) or Leiden clusters (Wolf *et al*, 2018; Traag *et al*, 2019) (based on 20 MOFA factors; resolution of 0.5 and 1.0, resulting in 12 and 24 clusters, respectively, Appendix Fig S2).

##### Computational complexity

From using the implementation described above, and using the LMM efficient implementation described by Lippert *et al* (2011; see more detail in Appendix Supplementary Methods) it follows that the complexity is O(N), where N is the minimum between the number of cells and the product of the number of unique individuals × the number of cellular contexts.

Empirical runtime was evaluated using observed expression profiles and contexts (MOFA factors) from the endoderm differentiation dataset (Cuomo *et al*, 2020a; Data ref: Cuomo *et al*, 2020b) and simulated individuals / genotypes. We assessed the runtime of CellRegMap and CellRegMap‐Association as a function of either the total number of cells (5,000, 7,500, 10,000, 12,500 or 15,000 cells sampled without replacement from the full dataset), the number of individuals (50, 75, 100, 125, 150) or the number of contexts tested for GxC effects (2, 5, 10, 15 or 20 leading MOFA factors; using the same number as background effects). Nonvarying parameters were set to a default of 10,000 cells, 100 donors and 10 tested contexts. All experiments were run on an Intel Xeon CPU E5‐2660 v4 with 2.00GHz and averaged across 125 simulated eQTL (Appendix Fig S1).

#### Application to endoderm differentiation

We considered single‐cell expression data from 33,964 cells across 125 individuals from (Cuomo *et al*, 2020a; Data ref: Cuomo *et al*, 2020b). These data cover *in vitro* differentiation of iPS cells from pluripotent stage (day0) to definitive endoderm (day3). Compared with the entire dataset considered in the original publication, we discarded two outlying cell sub‐populations (*n* = 475 and *n* = 1,212 cells, respectively).

##### Preprocessing of scRNA‐seq count data

Count data were processed as in the primary paper (Cuomo *et al*, 2020a; Data ref: Cuomo *et al*, 2020b), where counts were normalized using scran (Lun *et al*, 2016) and log‐transformed (log2(x + 1)). Prior to be inputed into the model as phenotype vectors (i.e., as y), single‐cell counts for a given gene were quantile‐normalized to better fit the Gaussian distribution assumed by the model. The log‐normalized count data (prior to quantile normalization) for the top 500 highly variable genes is also used as input for MOFA (see details below).

##### Estimation and annotation of MOFA factors

MOFA (Argelaguet *et al*, 2018) factors calculated from the expression profiles of the top 500 highly variable genes identified across all cells (using scran's function “modelGeneVar”), using default parameters. Annotation of the leading 10 MOFA factors was performed using gprofiler (Raudvere *et al*, 2019), considering the absolute loading of individual genes as estimated by MOFA. For each MOFA factor, we considered the top 20 enriched pathways to annotate individual factors (adjusted *P*‐values < 0.05; Fig EV2).

##### Testing for GxC effects

We considered *cis* eQTL from (Cuomo *et al*, 2020a; Data ref: Cuomo *et al*, 2020b). In particular, we considered all gene‐SNP pairs that were significant (FDR < 10%) in one or more of the developmental stages considered in the original paper, i.e., iPSCs, mesendoderm, definitive endoderm. In total, this corresponds to 4,470 eQTL pairs (3,240 unique Genes with an eQTL). This approach is similar to established strategies to test for GxE interactions, where SNPs with at least weak persistent effects (g only) are prioritized as candidates to assess GxE effects.

Next, we mapped context‐specific effects using either 1 or 10 MOFA factors. In both cases, all 20 MOFA factors were used to construct the background term, i.e., columns in C standardized and then used to build $\Sigma =CC^{T}$. To account for multiple testing, the resulting *P*‐values were adjusted at the gene‐level using the Bonferroni procedure to control the family‐wise error rate, followed by an adjustment across genes using the Storey method to control the false‐discovery rate (FDR). Significant results were reported at FDR < 5%.

##### Estimation of single‐cell effect sizes

We estimated both persistent genetic effects (βG) and cell‐level effect sizes due to GxC (β_GxC_; Appendix Supplementary Methods) for eQTL with significant GxC interactions (FDR < 5%), when considering either the first MOFA factor (MOFA 1, capturing differentiation) or the first 10 MOFA factors to define cell contexts.

##### Calibration of the GxC test

We evaluated calibration of the CellRegMap GxC interaction test by considering all genes on chromosome 22 (*n* = 270) and permuting the genotypes across individuals for two random SNPs per gene, finding calibrated test statistics. Additionally, we stratified genes by their correlation with differentiation time (the dominant axis of variation in this dataset) to rule out inflation of CellRegMap's test statistics in cases where the cell context has a strong effect on the outcome variable y (Appendix Fig S7A and C).

##### Alternative context definition

In order to assess the effects of the workflows used to define cell context, we considered chromosome 22 of the endoderm differentiation data (Cuomo *et al*, 2020a; Data ref: Cuomo *et al*, 2020b) (total number of eQTL: 121). We reassessed GxC effects when compare MOFA to alternative latent variable methods to definitions of contexts, namely using principal component analysis (PCA), linearly‐decoded variational autoencoder (LDVAE; Svensson *et al*, 2020), and zero‐inflated negative binomial‐based wanted variation extraction (ZINB‐WaVe; Risso *et al*, 2018). Both LDVAE and ZINB‐WaVe were run using a latent space dimension of 10 and accounting for the experiment identifier as a batch covariate. All other parameters were set to default values of the respective method. Despite differences on the level of the individual estimated factors using these different pipelines, CellRegMap's results were largely consistent demonstrating its robustness to the choice of factors used to capture context (Appendix Fig S5).

##### GxC interaction test (discovery)

We assessed an end‐to‐end workflow, which employs a two‐stage procedure, where CellRegMap‐Association is used first to identify candidate GxC eQTL prior to assessing interaction effects using CellRegMap (interaction test). Specifically, we considered 643 expressed genes on chromosomes 20–22 (*n* = 279, 53 and 270 on each chromosome, respectively) and used the CellRegMap‐Association to identify candidate variants to test for GxC effects. All analyses were based on a cis eindow of 100 kb window flanking the gene body, and SNPs with MAF > 5%. As expected, we observed that this orthogonal filter greatly reduced the number of GxC test compared with an exhaustive analysis (Appendix Fig S11A). More importantly, this filter also increased the total number of eQTL with detected GxC effects, indicating that this filter is helpful to mitigate the burden of multiple testing (Appendix Fig S11A).

#### Application to neuronal differentiation

We considered single‐cell transcriptomic data from over 200 individuals from (Jerber *et al*, 2021a; Data ref: Jerber *et al*, 2021b). We focused on a single cell type: midbrain dopaminergic neurons (DA), across three conditions defined in the original publication: day 30, day 52 untreated, and day 52 rotenone‐treated. In total, this consists of 135,435 cells from 210 donors.

##### Preprocessing of scRNA‐seq count data

Count data were processed following the procedure as outlined in the primary paper (Jerber *et al*, 2021a; Data ref: Jerber *et al*, 2021b), where counts were normalized using scanpy (Wolf *et al*, 2018) and log‐transformed (log2(x + 1)). Single‐cell counts for a given gene were quantile‐normalized to a Gaussian distribution prior to using them as input vector (y). In case of very large numbers of cells and sparse data (e.g., when considering 10X data), it may be appropriate to consider a pseudo‐cell approach (see below) to aggregate data across small numbers of cells to improve the signal‐to‐noise ratio.

##### Pseudo‐cell calculation

We obtained pseudo‐cells by clustering transcriptionally related cells in UMAP space using an approach similar to approaches described by Baran *et al* (2019) and DeTomaso *et al* (2019). Briefly, for each cell type we calculated the first 50 PCs across all conditions and donors, using all expressed genes (after QC, *n* = 32,738). Next, we applied batch‐correction for the experimental batches using Harmony (Korsunsky *et al*, 2019), as implemented in scanpy (“scanpy.external.harmony integrate”). Harmony‐adjusted PCs were then used to build a k‐NN (k = 10) graph based on Euclidean distances, separately for each condition and donor. Subsequently, cells were clustered using scanpy's implementation of the Leiden algorithm ((Traag *et al*, 2019), as implemented in “scanpy.tl.leiden”) at a resolution of 3.4.

The pseudo‐cell calculation as described above resulted in a total of 8,479 pseudocells (10–40 pseudocells per donor, 10–100 cells per pseudocell; Appendix Fig S8).

##### Clustering of single‐cell effect sizes

Next, we set out to cluster context‐specific eQTL based on their allelic effects due to GxC. We considered 212 eQTL which displayed significant GxC effects (FDR < 5%), identified using CellRegMap based on the leading 10 MOFA factors as cellular contexts. Next, we considered the estimated effect size profiles due to GxC, which were normalized to relative values in the range 0 to 1. We clustered these normalized profiles using SpatialDE (Svensson *et al*, 2018), using the same first 10 MOFA factors as spatial coordinates, and default parameters. This identified 12 clusters, containing between 2 and 43 genes (Fig 4B).

##### Cluster enrichment

For each cluster, we considered Pearson's correlation between the cluster's summary profiles (as outputted by SpatialDE) and single‐cell gene expression across all genes (*n* = 32,738). Next, we selected all genes with positive correlation larger than 0.4 and used gprofiler (Raudvere *et al*, 2019) to identify enriched pathways (considering Gene Ontology (GO) biological processes, molecular function, cellular components, pathways from KEGG Reactome and WikiPathways; miRNA targets from miRTarBase and regulatory motif matches from TRANSFAC; tissue specificity from Human Protein Atlas; protein complexes from CORUM and human disease phenotypes from Human Phenotype Ontology). The gprofiler function (“g:GOSt” as implemented in R) performs over‐representation analysis on input gene list (ordered by correlation level) using a hypergeometric test, corrected for multiple testing. The latter is performed using a tailor‐made method (g:SCS algorithm) which analytically approximates a threshold t corresponding to the 5% upper quantile of randomly generated queries of the provided size. All actual *P*‐values resulting from the query are transformed to corrected *P*‐values by multiplying these to the ratio of the approximate threshold t and the initial experiment‐wide threshold a = 0.05. Finally, we selected the top 3 significant (adjusted *P*‐values < 0.05) pathways per cluster (Fig EV4).

Further statistical details and derivations are provided in Appendix Supplementary Methods.

## Author contributions

**Anna SE Cuomo:** Conceptualization; data curation; software; formal analysis; investigation; visualization; methodology; writing – original draft; writing – review and editing. **Tobias Heinen:** Formal analysis; validation; writing – review and editing. **Danilo Horta:** Software; methodology. **John C Marioni:** Supervision; writing – original draft. **Oliver Stegle:** Conceptualization; supervision; funding acquisition; methodology; writing – original draft; project administration; writing – review and editing.

In addition to the CRediT author contributions listed above, the contributions in detail are:

AC and OS conceived the method. AC and DH implemented the software and methods with input from TH. AC performed computational analyses on real datasets. AC and TH devised the simulation strategy and TH conducted all experiments using simulated data. DV performed some data processing tasks. AC, JM and OS interpreted the results and wrote the manuscript.

## Disclosure and competing interests statement

OS is a paid advisor to Insitro, Inc. All the other authors declare that they have no conflict of interest.

## Supporting information



Appendix

Expanded View Figures PDF

Table EV1

Table EV2

Table EV3

Table EV4

Table EV5

## Data Availability

CellRegMap is available under an open‐source license at: https://github.com/limix/CellRegMap/. Code to reproduce the specific analyses presented here can be accessed under: https://github.com/annacuomo/CellRegMap_analyses/.
