# Supplementary material for: CellRegMap: a statistical framework for mapping context‐specific regulatory variants using scRNA‐seq
Source: Mol Syst Biol. 2022 Aug 16;18(8):e10663. doi: 10.15252/msb.202110663 (PMC9380406; doi:10.15252/msb.202110663)
Supplement: Supplementary file 1 — Appendix [file MSB-18-e10663-s001.pdf]

Appendix for:

CellRegMap: A statistical framework for mapping context-specific regulatory variants using single cell RNA-sequencing.

Anna S.E. Cuomo, Tobias Heinen, Danai Vagiaki, Danilo Horta,
John C. Marioni, Oliver Stegle

Contents

1 Supplementary Figures **2**

2 The CellRegMap model **14**
- 2.1 Model definition 14
- 2.2 Construction of the cellular context covariance 16
- 2.3 Statistical testing 16
- 2.4 Implementation 18

3 CellRegMap-Association test **20**

4 Predicting cell-specific effect sizes driven by GxC interactions **21**

5 Derivations **23**
- 5.1 Detailed derivations from section 4 23

References **26**

1 Supplementary Figures

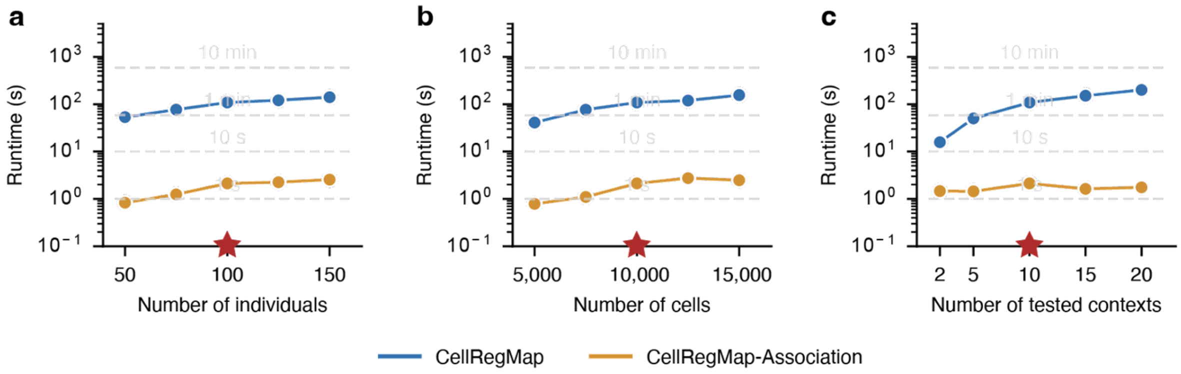

Appendix Figure S1: **Empirical assessment of the computational complexity of CellRegMap and CellRegMap-Association test.** Shown are empirical runtimes for simulated data (setup as described in **Materials and Methods**) averaged across 150 eQTL. Runtimes evaluated on an Intel Xeon CPU E5-2660 v4 with 2.00GHz. Shown are runtimes (y-axis) for testing a single eQTL as a function of the number of individuals **(a)**, the total number of cells **(b)** and the number of context variables **(c)**; runtimes are evaluated for both the CellRegMap model (interaction test, blue), and the corresponding association test (orange). Stars highlight default values for fixed parameters. All parameters retained at their default parameter values except the parameter that is indicated on the x axis.

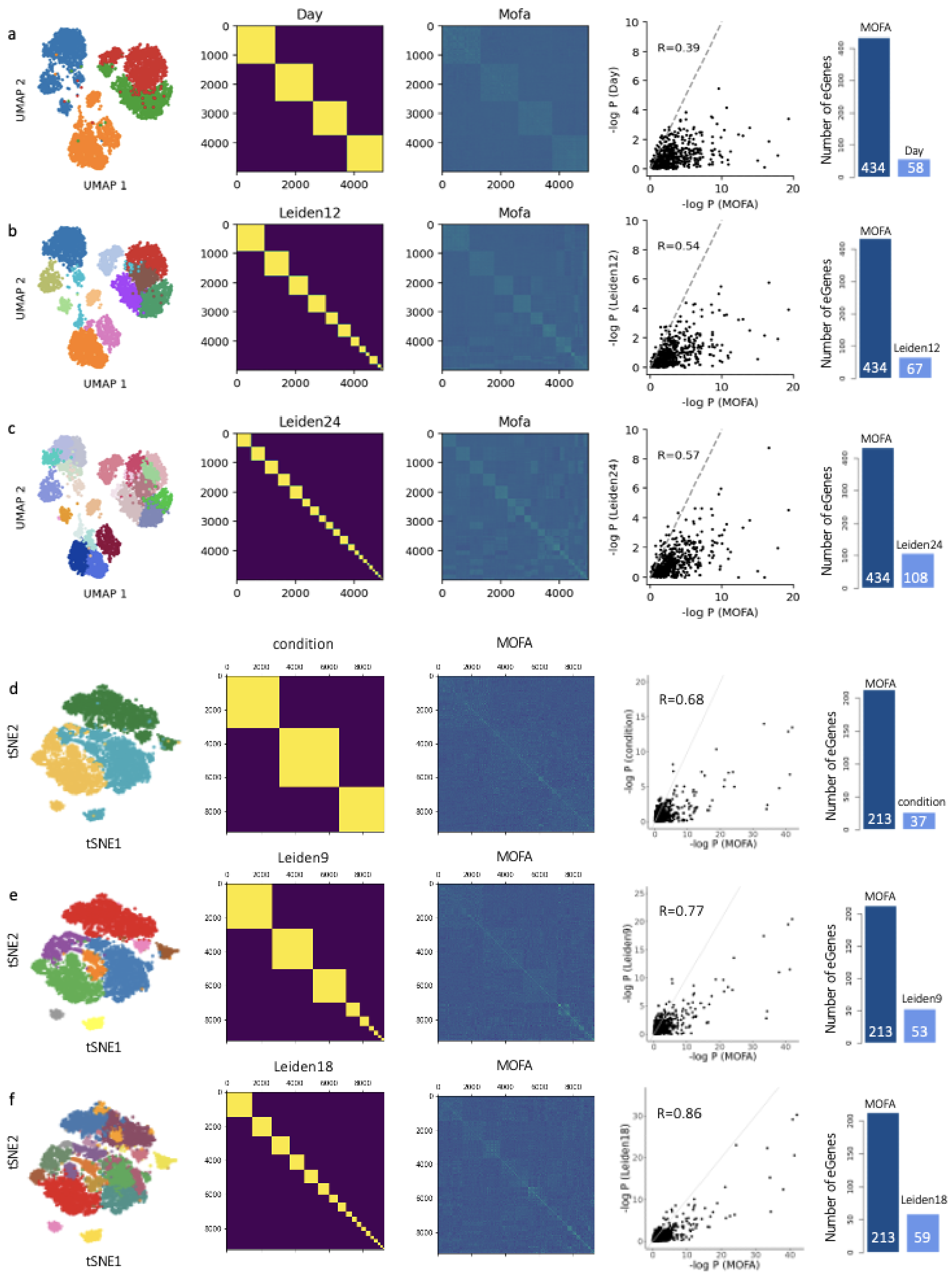

Appendix Figure S2: **Comparison of CellRegMap when using discrete cell contexts instead of continuous cell contexts on simulated and real data (Full legend on next page).**

Comparison of CellRegMap when using discrete cell contexts instead of continuous cell contexts on simulated and real data (continued). Concordance of results when using discrete vs. continuous contexts for CellRegMap. **Left:** 2-dimensional visualisation of the gene expression data and discrete clusters. **Middle:** Discrete and continuous covariance matrices (based on MOFA factors) sorted by cluster membership. **Right:** Result comparison; -log10(P-values) comparing models using continuous (x-axis) vs discrete (y-axis) contexts, as well as bar plots showing the number of significant GxC eQTL identified using the different context-covariance matrices. **(a-c)** Results for 500 semi-synthetic eQTL as used for the results presented in main text **Fig. 2** for the setting of 10 leading MOFA factors and the fraction of genetic variance explained by GxC = 0.5 (**Materials and Methods**). We consider discrete contexts at three different resolutions: **(a)** the original 4 sampling time points (Day), **(b)** 12 and **(c)** 24 Leiden clusters. Number of significant eGenes is out of 500 tested (FDR $<$ 10%). **(d-f)** Results on real data, considering the neuronal differentiation data from [1]. Results from running CellRegMap with 8,479 pseudo cells (**Materials and Methods**) and testing 2,051 eQTL (1,374 eGenes) in dopaminergic neurons from the original study. We compare results when using, as contexts, **(d)** the three discrete conditions from the original study (day 30, day 52 and rotenone-treated day 52; "condition"), **(e)** 9 and **(f)** 18 Leiden clusters, as opposed to 10 continuous MOFA factors, as done in the main analysis. Number of significant eGenes is out of 1,374 tested (FDR $<$ 5%, to match results from the main analysis).

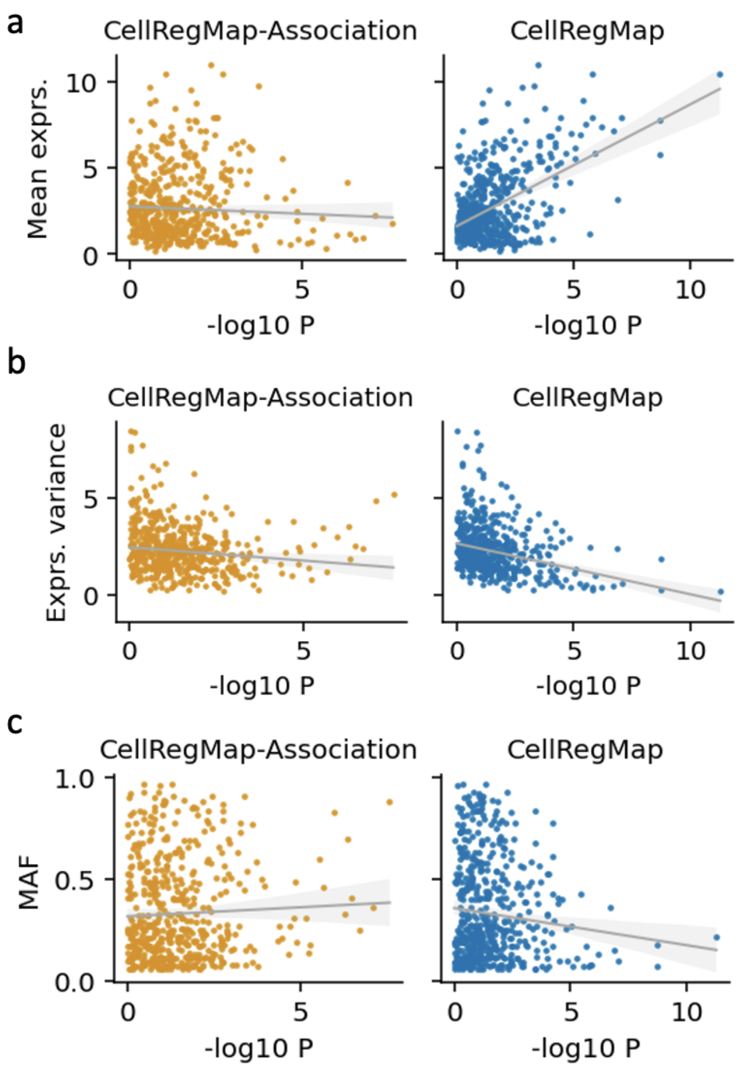

Appendix Figure S3: **eQTL statistics stratified for gene properties on simulated data.** Results for simulated genetic effects using the same parameters as in **Appendix Fig. S2** (10 MOFA factors with GxC, fraction of genetic variance explained by GxC = 0.5; **Materials and Methods**). Shown are the **(a)** mean observed gene expression of the simulated eGene (prior to adding the genetic effect), **(b)** the prior gene expression variance and **(c)** the minor allele frequency as a function of the P-value estimated by CellRegMap (blue) and CellRegMap-Association (orange). Both models are fitted using the same set of ground truth context variables as used in the simulation. Lines show the regression fit and shaded areas 95% confidence intervals.

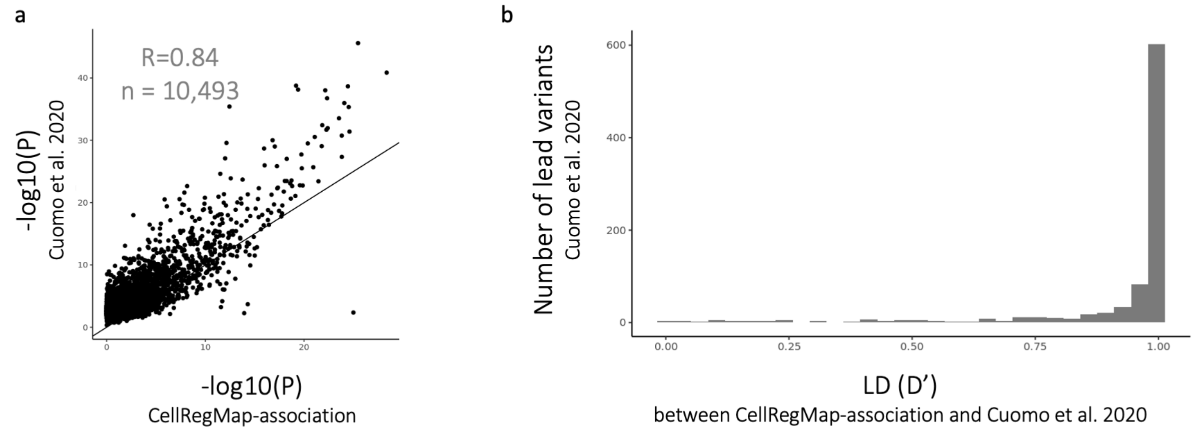

Appendix Figure S4: **Concordance of persistent genetic effects on endoderm differentiation data.** Data from [2]. **(a)** Scatter plot of -log10 p-values from the original study ([2], y-axis) vs -log10 p-values when using the CellRegMap-association test (x-axis). For the published study, the top variant from each gene is considered (n=10,493 genes). These results were achieved using aggregated "pseudocounts" from each of the three stages, and for each gene-SNP pair we are here selecting the smallest p-values between the iPSC, mesendoderm and definitive endoderm eQTL. **(b)** For each of the significant eGenes from the original study (n=2,996), we assessed whether the lead variant identified was the same or in linkage disequilibrium (LD) with the lead variant identified by CellRegMap-association. Histogram of the LD (D', as calculated using the R package "LDlinkR", [3]) distribution between lead variants from the two tests.

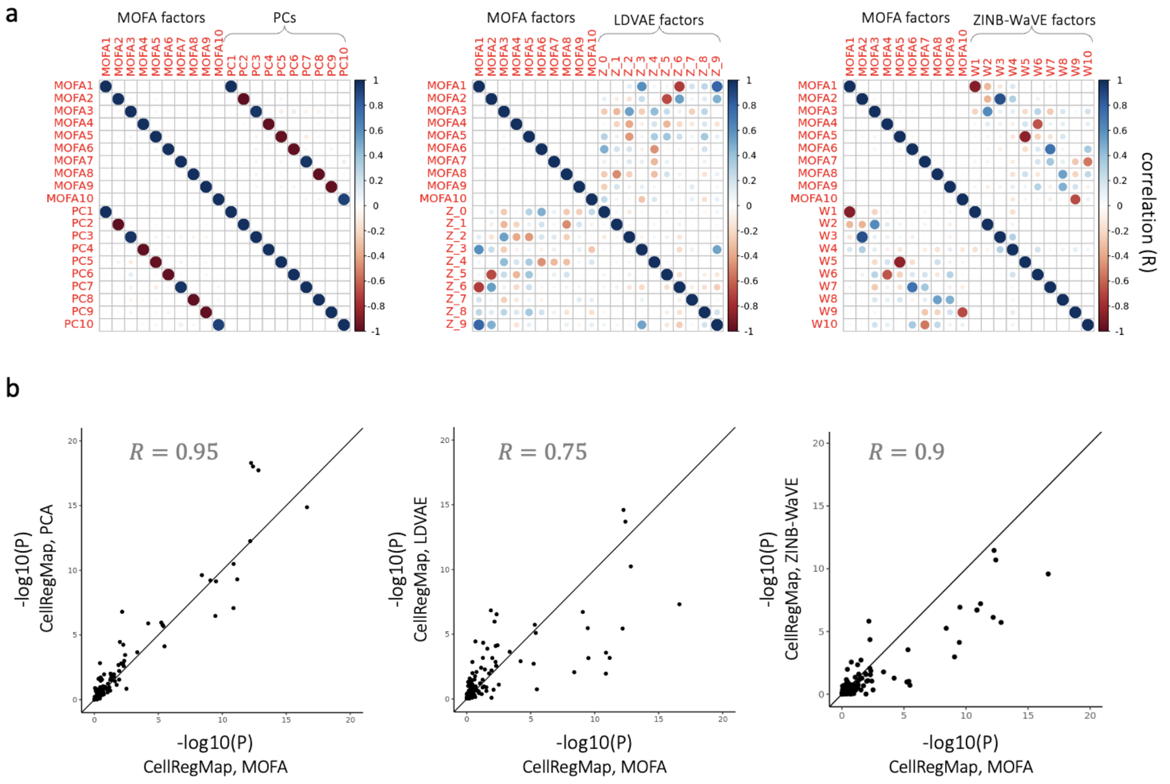

Appendix Figure S5: **Comparison of alternative methods to define cellular contexts.** Assessment based on the endoderm differentiation study [2]. We compared the MOFA workflow to define cellular contexts to principal component analysis (left), linearly-decoded variational autoencoder (LDVAE [4], middle) and zero-inflated negative binomial-based wanted variation extraction (ZINB-WaVe [5], right). **(a)** Correlation matrix between the leading 10 factors identified by MOFA and the respective alternative method. **(b)** Scatter plot of negative log p-value of the CellRegMap interaction test when using MOFA cell contexts (x-axis) versus using a cell-context covariance derived using the respective alternative method (y-axis). Considered are n=121 SNP-gene pairs from 88 unique genes on chromosome 22 only.

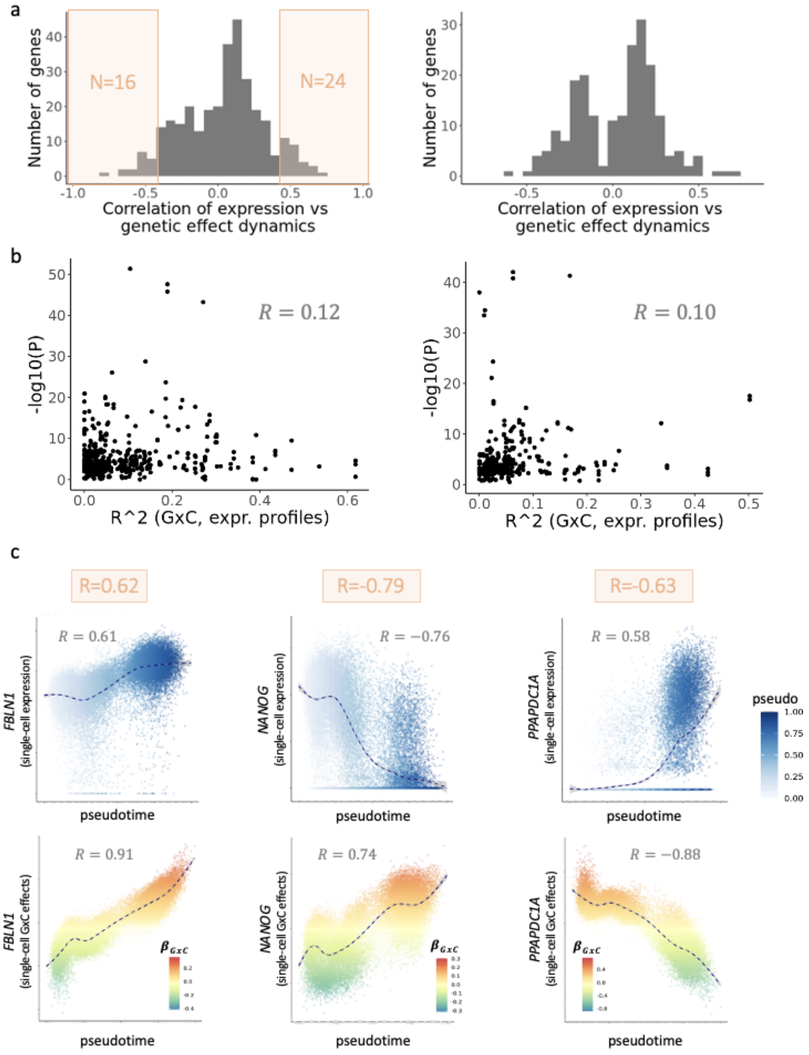

Appendix Figure S6: **Correlations of GxC with gene expression dynamics.** **(a)** Correlation (R) between single-cell expression profiles and estimated single-cell genetic effect profiles for 322 and 213 genes with significant GxC effects respectively (FDR<5%) (left: endoderm differentiation data from Cuomo et al [2], right: neuronal differentiation data from Jerber et al [1]). **(b)** Scatter plot between the R2 correlation coefficient between gene expression and GxC (x axis, as in **a**, but considering R2 instead of R) versus the statistical significance of GxC effects (-log10(p-value), y axis) (left: endoderm differentiation data from Cuomo et al, right: neuronal differentiation data from Jerber et al). **(c)** For three examples from **a** (high |R| between expression and GxC effects dynamics), the top row represents single-cell expression the genes (y-axis) as a function of pseudotime (x-axis); the bottom row represents the single-cell genetic effect estimates due to GxC (y-axis) as a function of pseudotime (x-axis). In orange, the R coefficient between expression and GxC effect dynamics, as in **a**.

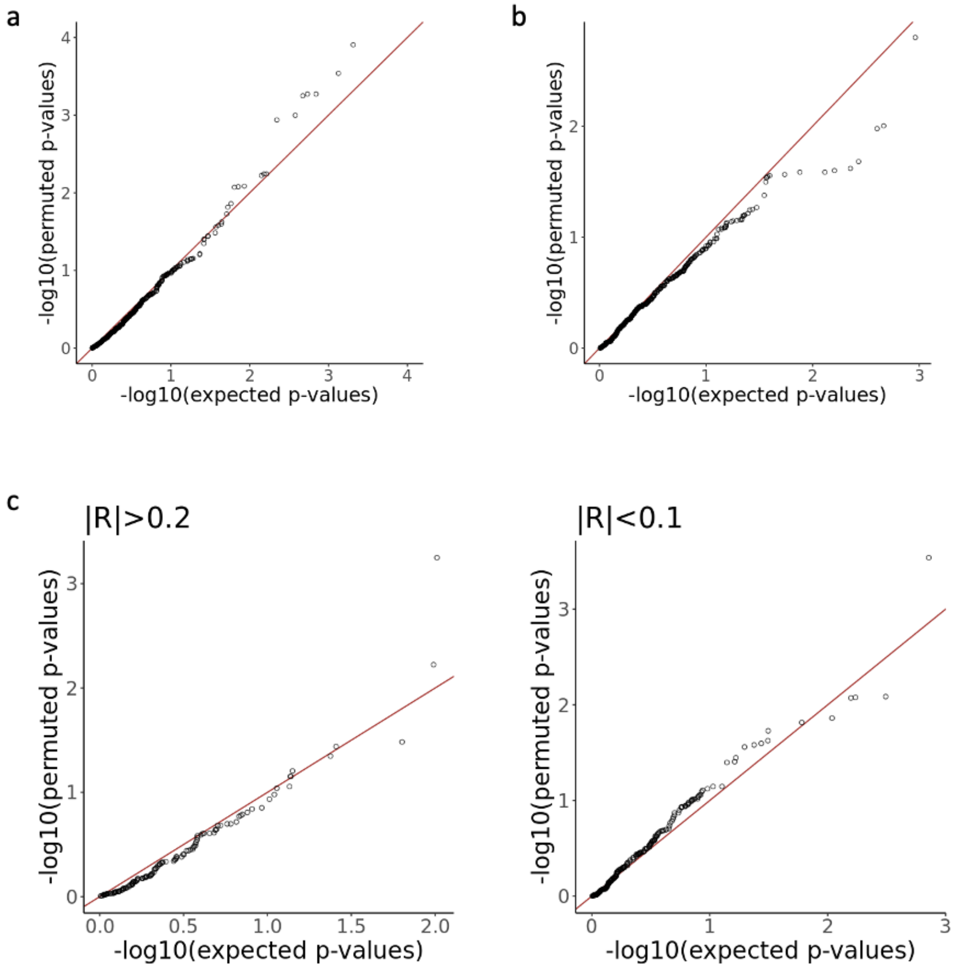

Appendix Figure S7: **Calibration of CellRegMap in relation to expression dynamics.** Shown are results obtained from the endoderm differentiation data [2]; (GxC interaction test) considering genes on chromosome 22 only. QQplots demonstrating calibration using permitted genotypes (GxC component only) **(a)** across all gene-SNP pairs from (n=540, i.e. 270 genes, 2 SNPs each): median=0.57; **(b)** across all gene-SNP pairs from (neuron differentiation data from Jerber et al, [3]) (n=500, *i.e.,* 250 genes, 2 SNPs each): median=0.51. **(c)** Considering the endoderm differentiation data, as in a, but stratifying genes by those whose expression is (left) correlated with differentiation time (|R| >0.2; n=134, median=0.51) and (right) not correlated with differentiation time (|R| <0.1; n=218, median=0.47).

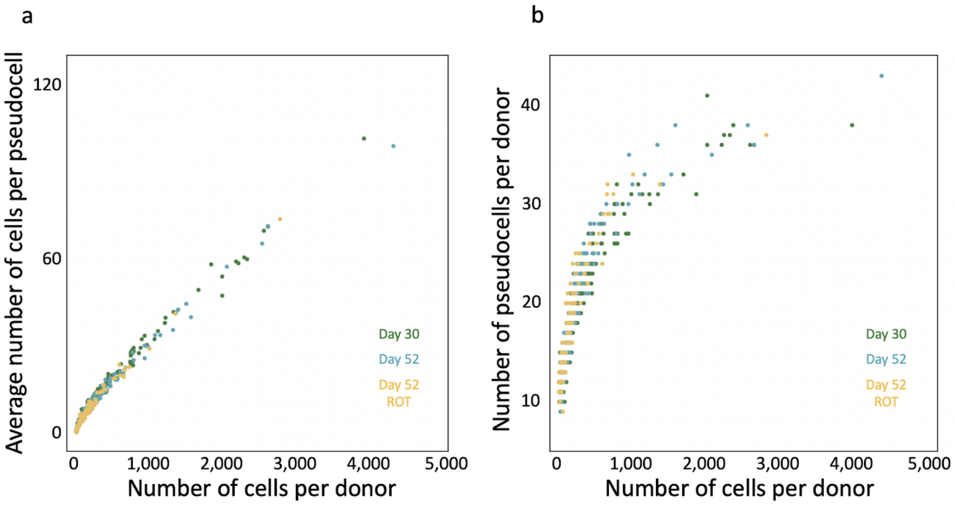

Appendix Figure S8: **Dopaminergic neuron pseudocell aggregation.** Transcriptionally related cells were aggregated into pseudocells, thereby reducing the sparsity in this dataset (dopaminergic neurons from [1]). Pseudocells were constructed separately for each individual and conditions (day 30, day 52, rotenone-treated day 52; **Materials and Methods**). **(a)** Scatter plot between the number of cells for each individual (x axis) versus the average number of cells contained in a pseudocell for the corresponding individual (y axis). Colour denotes the three main cell populations (collected across three conditions). **(b)** Analogous as in **a**, comparing the number of cells per donor versus the number of pseudocells per donor.

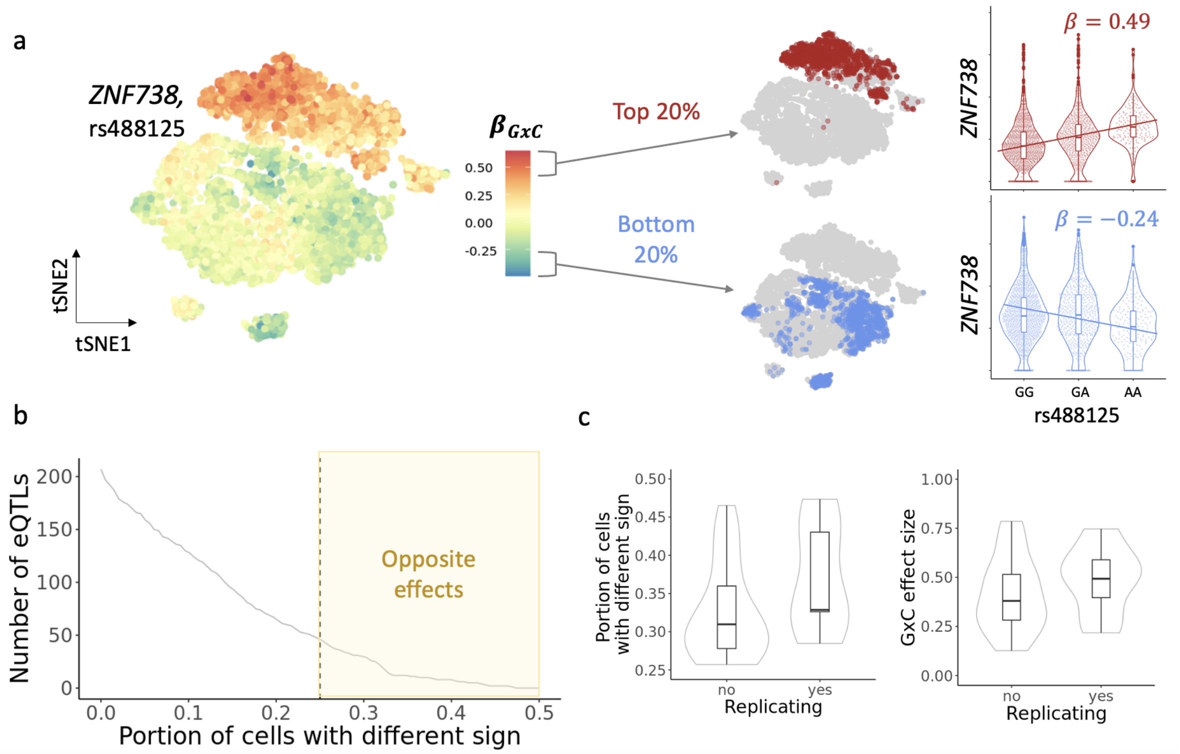

Appendix Figure S9: **Opposite effects detected by GxC.** Considering data from [2]. **(a)** Example of "true" opposite effects. The estimated genetic effects due to GxC (GxC) across all cells for the depicted eQTL (gene: *ZNF738*, SNP: chr19:21474173) indicates opposite effects across different populations, as shown by the tSNE plot on the left. To further assess this effect, we considered two extreme strata of cells (top and bottom 20% of the GxC values, tSNE plots in the middle) and assessed direction of effect in each stratum using a standard eQTL mapping strategy (based on pseudo bulk mapping; **Materials and Methods**), demonstrating true opposite effects (box plots on the right). **(b)** Distribution of the portion of cells with different sign from the rest in the GxC values (0 corresponds to the setting that indicates that GxC estimates for a given eQTL variant have consistent sign across all cells; 0.5 corresponds to 50% of all cells having an opposite effect signtake on assumes only positive values across all cells only, 1 negative for all cells), for all significant GxC eQTL (n=213, FDR<5%; neuronal development data). We define a GxC eQTL as displaying opposite effects when at least 25% of cells have a different sign compared to the rest in the estimated GxC values. **(c)** Next, we considered the 45 opposite effects from **(b)**, and assessed replication, by mapping standard eQTL when considering the top and bottom 20% of cells (based on GxC values, as shown for the example in a). When comparing the opposite effects that do replicate (opposite effect sizes using standard strategies as described in a; **Materials and Methods**) to those that do not replicate, we observe that (left) portion of cells with different signs is higher for the replicating ones (closer to a 50-50 split between positive and negative values), and (right) the estimated magnitude of GxC effect sizes (calculated as the delta of the top and bottom 10% of GxC values) is lower for the non-replicating opposite effects.

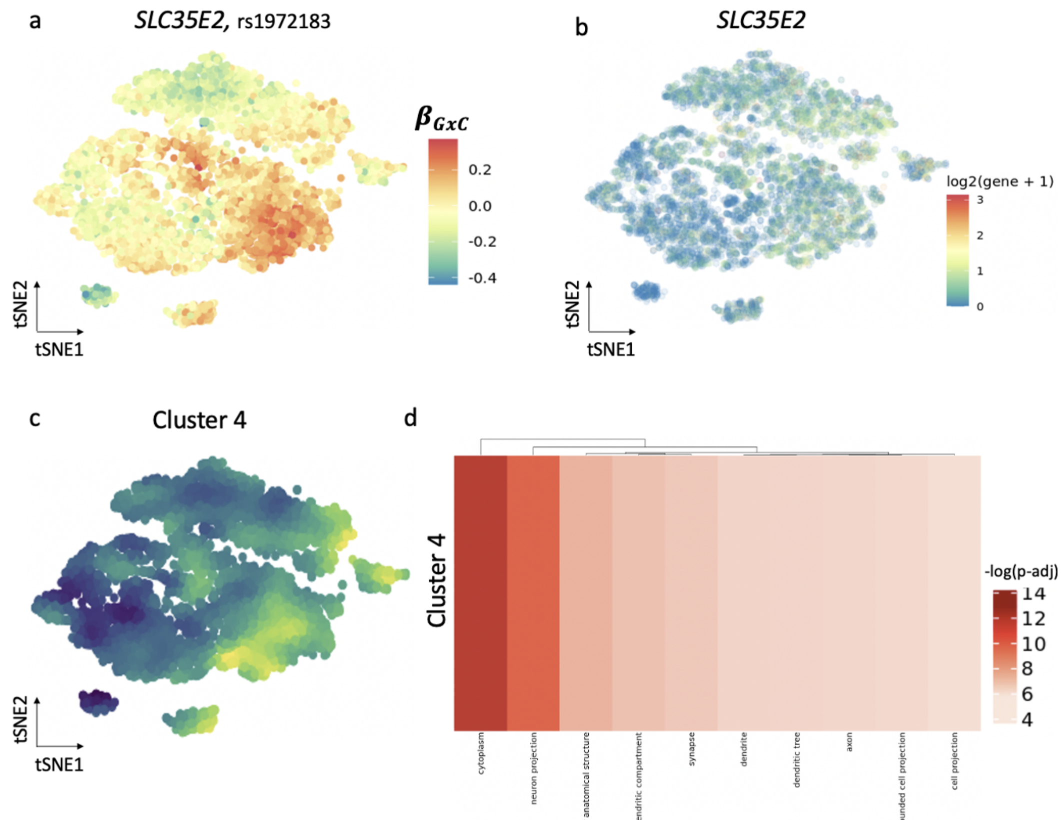

Appendix Figure S10: **CellRegMap to fine-map cellular context for human disease variant.** Considering the subset of cells identified as dopaminergic neurons from [1]. **(a)** GxC profile at rs1972183 for *SLC35E2*, which is colocalized with a GWAS variant for sleeplessness and insomnia in the subpopulation of day 52 untreated cells. Shown is a scatter plot of the first two tSNE coordinates with colour denoting the estimated GxC effect component GxC. **(b)** As in **a**, with colour denoting *SLC35E2* single-cell gene expression levels. **(c)** Manifold of consensus relative GxC effect sizes estimates for cluster 4, which contains the GxC effects for *SLC35E2*. Note that this figure is re-used from Main text Figure 4, panel d. **(d)** Extract of the gene enrichment analysis for cluster 4 (based on **Fig. EV4b**).

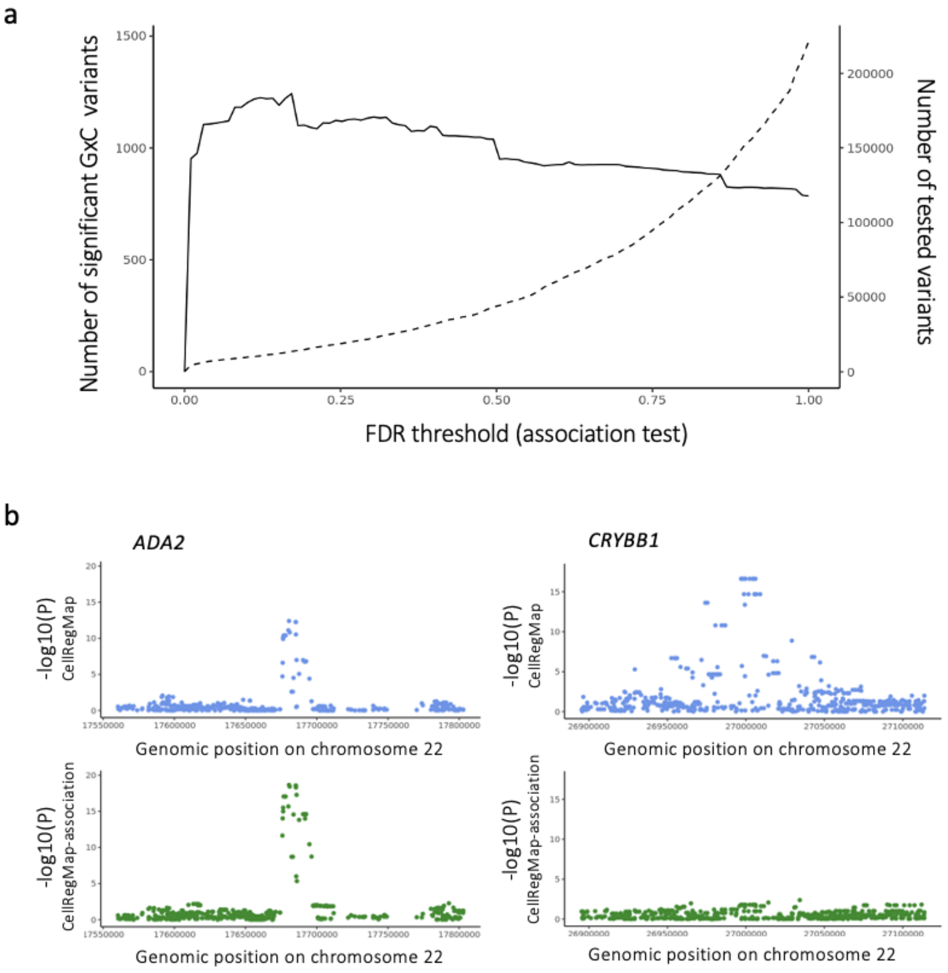

Appendix Figure S11: **Assessment of two-stage workflow using the CellRegMap-association test to define candidate eQTL for GxC analysis.** Results based on the endoderm differentiation data [2], using the de-novo for both persistent effects using the CellRegMap-association test and GxC interactions using the CellRegMap interaction test (+/- 100kb around the gene body, MAF>5%, all expressed genes on chromosomes 20-22). **(a)** Number of significant GxC effects (FDR<1%; y-axis) as a function of the FDR threshold to filter for variants based on the association signal (x axis). Second y-axis denotes the number of tested SNP-gene pairs. While the number of tested SNP-gene pairs increases for less stringent filtering, the number of detected GxC effects decreases, indicating that no discoveries are lost when considering the top eQTL only, while computation time is saved, and power to find GxC is also (slightly) increased. **(b)** Manhattan plots representing examples of same (left, for the *ADA2* gene) vs different (right, for the *CRYBB1* gene) signals being picked up by CellRegMap's GxC interaction test (blue) and CellRegMap association test (green).

2 The CellRegMap model

CellRegMap builds on and extends the structured linear mixed model (StructLMM [6]), which has recently been proposed to test for genotype-environment interactions on physiological traits in population cohorts. CellRegMap extends this model to test for interactions between genotype and cellular context on gene expression using single-cell RNA-seq as readout. The model is not designed for variant discovery but instead designed to identify and characterize genotype-context (G×C) interactions at known expression quantitative trait loci (eQTL). However, if eQTL are not known *a priori*, it is possible to run the Association test implemented within the CellRegMap software, in order to identify candidate eQTL to test for G×C interactions (section 3). In Section 2.1, the CellRegMap model is motivated and derived, followed by the introduction of an efficient scheme for parameter inference and statistical testing. Section 3 describes the association test implemented within CellRegMap, while section 4 describes the procedure to estimate cell-level effect sizes due to G×C.

2.1 Model definition

Commonly used methods for eQTL mapping based on linear or linear mixed models (LMM) relate individual genetic variants to the expression level of a gene of interest, *i.e.*,

$$\mathbf{y} = \mathbf{W}\boldsymbol{\alpha} + \mathbf{g}\beta_G + \boldsymbol{\psi}. \tag{1}$$

Here, $\mathbf{y}$ denotes a $N \times 1$ vector of the expression level of a gene of interest across N individuals (typically measured using bulk RNA sequencing); $\mathbf{W}$ is the $N \times P$ design matrix of covariates and $\alpha_i, i = 1, .., P$ are the corresponding weights (note that in the main text, *i.e.*, **Figure 1d**, covariates are omitted for brevity); $\mathbf{g}$ is the $N \times 1$ vector of alleles for each individual at the locus to be assessed and β_G denotes the corresponding effect size. Finally, $\boldsymbol{\psi}$ denotes i.i.d. noise, $\boldsymbol{\psi} \sim \mathcal{N}(\mathbf{0}, \sigma_n^2\mathbf{I})$. Additional random effect components have been introduced to account for relatedness between individuals [7], or to adjust for additive confounding sources of variation on gene expression [8].

Review of StructLMM

StructLMM [6] extends the conventional linear association test in Eq. (1) by including an additional random effect component to account for heterogeneity in effect sizes across individuals due to context-specific genetic effects. Briefly, StructLMM can be cast as:

$$\mathbf{y} = \mathbf{W}\boldsymbol{\alpha} + \mathbf{g}\beta_G + \mathbf{g} \odot \boldsymbol{\beta_{GxC}} + \mathbf{c} + \boldsymbol{\psi}, \tag{2}$$

where $\mathbf{g} \odot \boldsymbol{\beta_{GxC}}$ accounts for G × C and $\mathbf{c}$ for additive contributions of environmental variation. Unlike in conventional interaction tests, genotype-context interactions due to environmental variation are accounted by introducing an additional genetic effect with per-individual effects, where each individual has its own effect size. The symbol $\odot$ denotes the Hadamard product and $\boldsymbol{\beta_{GxC}} = [\beta_{GxC_1}, .., \beta_{GxC_N}]^T$ is a vector of per-individual effect sizes to account for heterogeneous genetic effects. Instead of explicitly estimating the GxC effect sizes, these

parameters are marginalised under a multivariate normal prior distribution that is defined by an environmental context covariance matrix:

$$\boldsymbol{\beta_{GxC}} \sim \mathcal{N}(\mathbf{0}, \sigma^2_{GxC}\mathbf{\Sigma}). \tag{3}$$

In this case the covariance $\mathbf{\Sigma}$ does not encode relatedness as in a classical LMM, but instead accounts for sample covariance due to different environmental states. The same environmental covariance matrix can also be used to parametrize a second random effect component to account for additive environmental contributions,

$$\mathbf{c} \sim \mathcal{N}(\mathbf{0}, \sigma^2_c\mathbf{\Sigma}). \tag{4}$$

Notably, StructLMM does not account for any additional structure across samples, such as population structure or repeat measurements. While discrete populations can be accounted for as covariates, more subtle relatedness or repeat structure cannot be effectively encoded using fixed effect covariates. Consequently, the model is not suitable for any experimental design where multiple observations are available for the same individual, which is the case in single-cell genetic studies, where multiple cells are assayed from each individual. Such a repeat structure results in un-calibrated p-values; see also the results from the simulation study to assess calibration (main text **Fig. 2**).

CellRegMap

CellRegMap extends StructLMM to allow for applications to single-cell expression data by introducing an additional random effect component that accounts for relatedness or sample repeat structure. First, we note that the phenotype $\mathbf{y}$ now represents single-cell resolved expression data, so our samples are expression levels in cells, not individuals. This introduces additional structure in the data, as typically multiple cells are sampled from the same individual. To account for this structure, an additional random effect component $\mathbf{u}$ is included in the model:

$$\mathbf{y} = \mathbf{W}\boldsymbol{\alpha} + \mathbf{g}\beta_G + \mathbf{g} \odot \boldsymbol{\beta_{GxC}} + \mathbf{c} + \mathbf{u} + \boldsymbol{\psi}. \tag{5}$$

Here, $\mathbf{y}$ has the cardinality of single-cells rather than individuals, and hence the G×C component ($\mathbf{g} \odot \boldsymbol{\beta_{GxC}}$) accounts for interactions with cellular states and contexts (which are well defined at the level of single cells) as well as environmental exposures and stimuli (which can also be individual-level). The terms $\mathbf{c} \sim \mathcal{N}(\mathbf{0}, \sigma^2_c\mathbf{\Sigma})$ and $\boldsymbol{\psi} \sim \mathcal{N}(\mathbf{0}, \sigma^2_n\mathbf{I})$ have the same meaning as previously, noting that for these $N \times 1$ vectors N now represents the total number of cells, not the number of unique individuals. Similarly, $\mathbf{\Sigma}$) is $N \times N$ and is again defined in the space of cells. The symbol $\boldsymbol{\beta_{GxC}} \sim \mathcal{N}(\mathbf{0}, \sigma^2_{GxC}\mathbf{\Sigma})$ now represents cell-level effect sizes, which again captures variation in genetic effects across cells. The additional random effect component $\mathbf{u}$ accounts for relatedness or the repeat structure, which is parametrized as a product kernel between relatedness ($\mathbf{R}$) and the environmental covariance ($\mathbf{\Sigma}$):

$$\mathbf{u} \sim \mathcal{N}(\mathbf{0}, \sigma^2_{rc}\mathbf{R} \odot \mathbf{\Sigma}). \tag{6}$$

Here, $\mathbf{R}$ denotes the relatedness matrix of individuals expanded to all cells based on the known assignment of cells to individuals and the covariance $\mathbf{\Sigma}$ again denotes the cell-level environmental context. Notably, this parametrization extends the classical LMM, which would exclusively consider a relatedness component $\mathbf{R}$. One way to interpret this covariance is to account for polygenic interactions between environmental and relatedness, which has previously been considered to estimate the GxE component of heritability [9].

2.2 Construction of the cellular context covariance

Typically, we define $\mathbf{\Sigma} = \mathbf{E}\mathbf{E}^T$, and hence the cellular context covariance is a linear function of a matrix of environmental states $\mathbf{E}$. In practice, we consider as cellular contexts axes of variation in the dataset (for example captured by principal components or MOFA [10] factors), appropriately standardized (mean=0, standard deviation=1) and build $\mathbf{\Sigma} = \mathbf{E}\mathbf{E}^T$ accordingly. Depending on the type and structure of cellular contexts, $\mathbf{\Sigma}$ can simply separate cells into groups, and appear as a block diagonal or capture continuous transitions (main text **Fig. 1c**). In principle, CellRegMap can also be use in conjunction with other parametrizations of the cell context covariance.

2.3 Statistical testing

The main operations in the CellRegMap model, including parameter inference and tests are implemented in its marginalised form. Integrating over the random effect components, the marginal likelihood of the model in Eq. (5) follows as:

$$\mathbf{y} \sim \mathcal{N}(\mathbf{W}\boldsymbol{\alpha} + \mathbf{g}\beta_G, \sigma^2_{GxC}\text{diag}(\mathbf{g})\mathbf{\Sigma}\text{diag}(\mathbf{g}) + \sigma^2_c\mathbf{\Sigma} + \sigma^2_{rc}\mathbf{R} \odot \mathbf{\Sigma} + \sigma^2_n\mathbf{I}). \tag{7}$$

While in principle CellRegMap can be used to test for different components, including additive genetics, interactions or the combination of both effects (see [6] for details on StructLMM), we here focus on GxC effects. In order to evaluate the significant contribution of GxC effects, we consider a statistical test that compares the following hypotheses, from Eq.(5):

$$H_0 : \sigma^2_{GxC} = 0,$$

$$H_1 : \sigma^2_{GxC} > 0.$$

We use Rao's Score test [11] to evaluate significance, which allows us to only calculate the MLE[1] of the parameters under the null hypothesis H_0, which follows from Eq.(7) as:

$$\mathbf{y}|H_0 \sim \mathcal{N}(\mathbf{W}\boldsymbol{\alpha} + \mathbf{g}\beta_G, \sigma^2_c\mathbf{\Sigma} + \sigma^2_{rc}\mathbf{R} \odot \mathbf{\Sigma} + \sigma^2_n\mathbf{I}). \tag{8}$$

[1]maximum likelihood estimator

To test for GxC interactions we adapt the score-based testing scheme employed in StructLMM, which in turn adopts fast LMM testing in LMMs as first proposed in Lippert et al., [12]. We here review the key steps involved.

First, we define the score-based test statistic Q as:

$$\mathrm{Q} = \frac{1}{2}\mathbf{y}^T\mathbf{P}_0\frac{\partial\mathbf{K}}{\partial\theta}\mathbf{P}_0\mathbf{y}, \tag{9}$$

where $\mathbf{K}$ denotes the full covariance matrix, *i.e.*, from Eq. (5):

$$\mathbf{K} = \sigma^2_{GxC}\mathrm{diag}(\mathbf{g})\mathbf{\Sigma}\ \mathrm{diag}(\mathbf{g}) + \sigma^2_c\mathbf{\Sigma} + \sigma^2_{rc}\mathbf{R}\odot\mathbf{\Sigma} + \sigma^2_n\mathbf{I}, \tag{10}$$

and

$$\mathbf{P}_0 = \mathbf{K}_0^{-1} - \mathbf{K}_0^{-1}\mathbf{X}(\mathbf{X}^T\mathbf{K}_0^{-1}\mathbf{X})^{-1}\mathbf{X}^T\mathbf{K}_0^{-1} \tag{11}$$

is a matrix that projects out the fixed effects [12, 13]. In our case (Eq.(5)), the fixed effects include covariates and the persistent effect of the variant tested: $\mathbf{X} = [\mathbf{W}, \mathbf{g}]$, and $\mathbf{K}_0$ is as in Eq.(23). Using Eq.(10) and considering the parameter $\theta = \sigma^2_{GxC}$, we can derive:

$$\frac{\partial\mathbf{K}}{\partial\sigma^2_{GxC}} = \mathrm{diag}(\mathbf{g})\mathbf{\Sigma}\ \mathrm{diag}(\mathbf{g}). \tag{12}$$

Next, let us define $\mathbf{K}_1 = \mathrm{diag}(\mathbf{g})\mathbf{\Sigma}\ \mathrm{diag}(\mathbf{g})$; substituting in Eq.(9), we can write:

$$\mathrm{Q} = \frac{1}{2}\mathbf{y}^T\mathbf{P}_0\mathbf{K}_1\mathbf{P}_0\mathbf{y}. \tag{13}$$

As before, $H_1 : \sigma^2_{GxC} > 0$, noting that as a variance parameter, σ^2_{GxC} is constrained to take on positive values. As a result, the score test statistic Q follows a mixture of χ^2 distributions[2]:

$$\mathrm{Q} \sim \sum_i \lambda_i\chi^2_1, \tag{14}$$

where λ_i's are the non-zero eigenvalues of $\frac{1}{2}\mathbf{P}_0^{\frac{T}{2}}\frac{\partial\mathbf{K}}{\partial\theta}\mathbf{P}_0^{\frac{1}{2}}$.

It can be shown that for a matrix $\mathbf{A}$ the non-zero eigenvalues of $\mathbf{A}\mathbf{A}^T$ are the same as those of $\mathbf{A}^T\mathbf{A}$, thus we can re-arrange and compute λ_i's as the eigenvalues of:

$$\frac{1}{2}\frac{\partial\mathbf{K}}{\partial\theta}^{\frac{T}{2}}\mathbf{P}_0\frac{\partial\mathbf{K}}{\partial\theta}^{\frac{1}{2}} \tag{15}$$

instead. To evaluate the significance of the score-best test statistic Q we use the approach described in Sequence Kernel Association Test (SKAT [14]), thereby using the Davies exact method [15] to compute the corresponding p values, and switching to the modified moment matching approximation method [16, 17, 18] when this fails to converge.

[2]We refer the reader specifically to the supplementary methods from [13] for a proof.

2.4 Implementation

To enable efficient parameter inference, we extended the strategy in StructLMM [6], which builds on the reparametrization of the LMM likelihood proposed in [12]. Briefly, the key is to rewrite the overall covariance of $\mathbf{y}|H_0$ in the form: $\sigma_m^2(\mathbf{M}+\delta\mathbf{I})$, where $\mathbf{M}$ is ideally low rank to enable an efficient singular value decomposition. To do so, we introduce a weight parameter ρ_1, such that the covariance matrix of $\mathbf{y}$ under the null hypothesis, $\mathbf{K}_0 = \mathrm{Cov}(\mathbf{y}|H_0)$ can be cast as:

$$\begin{aligned}\mathbf{K}_0 = \sigma_c^2\boldsymbol{\Sigma} + \sigma_{rc}^2\mathbf{R}\odot\boldsymbol{\Sigma} + \sigma_n^2\mathbf{I} = \\ \sigma_m^2\ [\rho_1\boldsymbol{\Sigma} + (1-\rho_1)\mathbf{R}\odot\boldsymbol{\Sigma}] + \sigma_n^2\mathbf{I} = \\ \sigma_m^2\{\mathbf{M}(\rho_1) + \delta_1\mathbf{I}\},\end{aligned} \tag{16}$$

where $\sigma_m^2\rho_1 = \sigma_c^2$, $\sigma_m^2(1-\rho_1) = \sigma_{rc}^2$, $\delta_1 = \sigma_n^2/\sigma_m^2$, and $\mathbf{M}(\rho_1) = \rho_1\boldsymbol{\Sigma} + (1-\rho_1)\mathbf{R}\odot\boldsymbol{\Sigma}$.

We note that $\mathbf{M}$ only depends on ρ_1. The parameter ρ_1 is optimized using a grid search with values in the range 0 to 1, selecting the value of ρ_1 that maximises the likelihood of $\mathbf{y}|H_0$ under the null. Once ρ_1 is fixed, the decomposition of $\rho_1\boldsymbol{\Sigma} + (1-\rho_1)\mathbf{R}\odot\boldsymbol{\Sigma}$ can be found using the observation that the decomposition of the linear combination of two square symmetric matrices, *e.g.,*:

$$\mathbf{M} = a\mathbf{A} + b\mathbf{B} \tag{17}$$

can be found as a function of the decomposition of the two original matrices, *i.e.,*:

$$\mathbf{N} = [\sqrt{a}\mathbf{C}|\sqrt{b}\mathbf{D}] \tag{18}$$

such that $\mathbf{M} = \mathbf{N}\mathbf{N}^T, \mathbf{A} = \mathbf{C}\mathbf{C}^T$ and $\mathbf{B} = \mathbf{D}\mathbf{D}^T$. In our case, $\mathbf{A} = \boldsymbol{\Sigma}$ and $\mathbf{B} = \mathbf{R}\odot\boldsymbol{\Sigma}$. If $\boldsymbol{\Sigma} = \mathbf{E}\mathbf{E}^T$, its decomposition is straight-forward, with $\mathbf{C} = \mathbf{E}$. The decomposition of the second term ($\mathbf{R}\odot\boldsymbol{\Sigma}$), assuming $\boldsymbol{\Sigma} = \mathbf{E}\mathbf{E}^T$ and $\mathbf{R} = \mathbf{G}\mathbf{G}^T$ is a bit less trivial. Assuming that the rank of $\mathbf{R}$ is the number of individuals, that the rank of $\boldsymbol{\Sigma}$ is the number of environments, and that the latter is smaller than the former, we can obtain $\mathbf{D}$ as the decomposition of $\mathbf{R}\odot\boldsymbol{\Sigma}$ by:

$$\mathbf{R}\odot\boldsymbol{\Sigma} = \mathbf{R}\odot\sum_i[\mathbf{v}_i\lambda_i\mathbf{v}_i^T] = \sum_i[\mathbf{R}\odot(\mathbf{v}_i\lambda_i\mathbf{v}_i^T)]. \tag{19}$$

The terms in the sum can be rewritten as:

$$
\begin{aligned}
\mathbf{R} \odot (\mathbf{v}_i \lambda_i \mathbf{v}_i^T) &= \mathbf{R} \odot (\mathbf{u}_i \mathbf{u}_i^T) \\
&= \mathbf{R} \odot (\mathrm{diag}(\mathbf{u}_i)\mathbf{e}\mathbf{e}^T \mathrm{diag}(\mathbf{u}_i)) \\
&= \mathrm{diag}(\mathbf{u}_i)[\mathbf{R} \odot \mathbf{e}\mathbf{e}^T]\mathrm{diag}(\mathbf{u}_i) \\
&= \mathrm{diag}(\mathbf{u}_i)\mathbf{R}\mathrm{diag}(\mathbf{u}_i) \\
&= \mathrm{diag}(\mathbf{u}_i)\mathbf{G}\mathbf{G}^T \mathrm{diag}(\mathbf{u}_i) \\
&= \mathbf{Lk}_i \mathbf{Lk}_i^T,
\end{aligned}
\tag{20}
$$

where $\mathbf{Lk}_i = \mathrm{diag}(\mathbf{u}_i)\mathbf{G}$; λ_i's and $\mathbf{v}_i$'s are eigenvalues and eigenvectors of $\mathbf{E}\mathbf{E}^T$; $\mathbf{u}_i = \sqrt{\lambda_i}\mathbf{v}_i$ and $\mathbf{e}$ is a vector of ones ($\mathbf{e} = [1..1]$).

Thus, $\mathbf{M} = \mathbf{\Sigma} + \mathbf{R} \odot \mathbf{\Sigma}$ can be written as the following sum:

$$
\mathbf{M} = \mathbf{\Sigma} + \sum_i \mathbf{Lk}_i \mathbf{Lk}_i^T, \tag{21}
$$

from which follows:

$$
\mathbf{N} = [\mathbf{E}\ |\mathbf{Lk}_1\ |..\ |\mathbf{Lk}_{ne}], \tag{22}
$$

where ne is the number of contexts considered (and the rank of $\mathbf{\Sigma}$).

3 CellRegMap-Association test

Within the CellRegMap software suite it is also possible to test for persistent genetic effects, while appropriately account for cellular context. This model essentially draws from the null hypothesis of CellRegMap, thus comparing the following two models (where we exclude the G× term from Eq. (5)):

$$H_0 : \mathbf{y} = \mathbf{W}\boldsymbol{\alpha} + \mathbf{c} + \mathbf{u} + \boldsymbol{\psi},$$

vs

$$H_1 : \mathbf{y} = \mathbf{W}\boldsymbol{\alpha} + \mathbf{g}\beta_G + \mathbf{c} + \mathbf{u} + \boldsymbol{\psi}.$$

All variables are defined as in section 2, with the exception of $\mathbf{u}$, which here is defined to account for the repeated structure due to many cell been drawn from the same individual:

$$\mathbf{u} \sim \mathcal{N}(\mathbf{0}, \sigma_r^2 \mathbf{R}).$$

This simplifies the implementation, where we note that Eq. (23) becomes:

$$\begin{aligned} \mathbf{K}_0 = \sigma_c^2 \boldsymbol{\Sigma} + \sigma_r^2 \mathbf{R} + \sigma_n^2 \mathbf{I} = \\ \sigma_m^2 \ [\rho_1 \boldsymbol{\Sigma} + (1 - \rho_1)\mathbf{R}] + \sigma_n^2 \mathbf{I} = \\ \sigma_m^2 \{\mathbf{M}'(\rho_1) + \delta_1 \mathbf{I}\}, \end{aligned} \tag{23}$$

from which follows:

$$\mathbf{M} = \boldsymbol{\Sigma} + \mathbf{R}, \tag{24}$$

and

$$\mathbf{N} = [\mathbf{E} \ |\mathbf{G}], \tag{25}$$

where we assume, as before, $\boldsymbol{\Sigma} = \mathbf{E}\mathbf{E}^T$ and $\mathbf{R} = \mathbf{G}\mathbf{G}^T$.
To assess significance, we use a likelihood ratio test (LRT). This is well defined since we are testing $\beta_G \neq 0$, which is not at the boundary of possible values of the parameter, as $-\infty < \beta_G < \infty$.

4 Predicting cell-specific effect sizes driven by GxC interactions

Using CellRegMap it is possible to estimate cell-level allelic effects due to GxC, (thus estimating $\boldsymbol{\beta_{GxC}}$ from eq. (7)) for each gene-SNP pair tested. For this derivation, we will use the function representation of linear mixed model as a Gaussian process ($\mathcal{GP}$). Briefly (for more details see section 5.1),

$$\mathbf{f} \sim \mathcal{GP}\{\mathbf{m}; k(\mathbf{X}, \mathbf{X})_{\boldsymbol{\theta}}\}, \tag{26}$$

or:

$$\mathbf{f} = \mathbf{m} + \mathbf{c}, \tag{27}$$

where we define:

$$\mathbf{m} = \mathbf{X}\boldsymbol{\beta}_{\theta} \;\text{ and }\; \mathbf{c} \sim \mathcal{N}(\mathbf{0}, k(\mathbf{X}, \mathbf{X})_{\theta}). \tag{28}$$

When we model our data ($\mathbf{X}$ and $\mathbf{y}$), we consider $\mathbf{y}$ as being a sample from the random variable $\mathbf{f}$, and $\mathbf{X}$ as being fixed. Let us collectively call the parameters $\boldsymbol{\theta}$ and $\hat{\boldsymbol{\theta}}_{BLUP}$ (or $\hat{\boldsymbol{\theta}}$) their best linear unbiased predictor (BLUP) estimator [19].

For out of sample ($*$) prediction, we can define [20]:

$$\mathbf{y}_* \coloneqq \mathrm{E}[\mathbf{f}_*|\mathbf{y}]_{\hat{\boldsymbol{\theta}}}, \tag{29}$$

which can be written as (see section 5.1 for intermediate steps):

$$\mathbf{y}_* = \mathbf{m}_* + k(\mathbf{X}_*, \mathbf{X})k(\mathbf{X}, \mathbf{X})^{-1}(\mathbf{y} - \mathbf{m}). \tag{30}$$

In our case, we use a modified model from eq. (5) where the additive environments are modelled as fixed effects:

$$\mathbf{y} = \overbrace{\mathbf{W}\boldsymbol{\alpha} + \mathbf{g}\beta_G + \mathbf{E}\boldsymbol{\gamma}}^{\mathbf{m}} + \overbrace{\mathbf{g} \odot \boldsymbol{\beta}_{GxE} + \mathbf{u} + \boldsymbol{\psi}}^{\mathbf{c}}. \tag{31}$$

By substituting $\mathbf{m}$ and $\mathbf{c}$ into eq. (30) we obtain a formula for $\mathbf{y}_*$:

$$\mathbf{y}_* = \mathbf{W}_*\boldsymbol{\alpha} + \mathbf{g}_*\beta_G + \mathbf{E}_*\boldsymbol{\gamma} + k(\mathbf{X}, \mathbf{X}_*)k(\mathbf{X}, \mathbf{X})^{-1}(\mathbf{y} - \mathbf{W}\boldsymbol{\alpha} + \mathbf{g}\beta_G + \mathbf{E}\boldsymbol{\gamma}). \tag{32}$$

Now, to obtain an estimate for $\boldsymbol{\beta}_*$, we can evaluate eq. (32) when $\mathbf{g}_* = 0$ ($\mathbf{y}_*$(ref)) and when $\mathbf{g}_* = 1$ ($\mathbf{y}_*$(alt)), and consider the difference, scaled by a number based on the MAF[3] (p):

[3] minor allele frequency

$$\boldsymbol{\beta}^*_{GxC} = \frac{1}{\sqrt{2p(1-p)}} \left(\mathbf{y}_*(\text{alt}) - \mathbf{y}_*(\text{ref})\right), \tag{33}$$

from which, skipping a few steps:

$$\boldsymbol{\beta}^*_{GxC} = \frac{1}{\sqrt{2p(1-p)}} \left\{\mathbf{1}\beta_G + \sigma^2_{GxC}\mathbf{E}_*(\mathbf{g}\odot\mathbf{E})^T\mathbf{K}^{-1}(\mathbf{y} - \mathbf{W}\boldsymbol{\alpha} - \mathbf{g}\beta_G - \mathbf{E}\boldsymbol{\gamma})\right\}, \tag{34}$$

where $\mathbf{K} = \text{Cov}(\mathbf{y}) = \sigma^2_{GxE}(\mathbf{g}\odot\mathbf{E})(\mathbf{g}\odot\mathbf{E})^T + \sigma^2_{re}\mathbf{R}\odot\mathbf{E}\mathbf{E}^T + \sigma^2_n\mathbf{I}$.

By setting $\mathbf{E}_* = \mathbf{E}$, we can perform in-sample estimation of allelic effects.

5 Derivations

5.1 Detailed derivations from section 4

A linear mixed model is a type of Gaussian Process, that can be written as:

$$\mathbf{f} \sim \mathcal{GP}\{\mathbf{m}; k(\mathbf{X}, \mathbf{X})_{\boldsymbol{\theta}}\}, \tag{35}$$

or, equivalently, as:

$$\mathbf{f} = \mathbf{m} + \mathbf{u}, \tag{36}$$

where

$$\mathbf{m} = \mathbf{X}\boldsymbol{\beta}_\theta \;\text{ and }\; \mathbf{u} \sim \mathcal{N}(\mathbf{0}, k(\mathbf{X}, \mathbf{X})_\theta). \tag{37}$$

When we model our data ($\mathbf{X}$ and $\mathbf{y}$), we consider $\mathbf{y}$ as being a sample from the random variable $\mathbf{f}$, and $\mathbf{X}$ as being fixed. Let us collectively call the parameters $\boldsymbol{\theta}$. For all parameters, we can find the set of best linear unbiased predictor (BLUP) estimators ($\hat{\boldsymbol{\theta}}_{BLUP}$ or simply $\hat{\boldsymbol{\theta}}$) which i) minimise the variance and ii) are unbiased [19]. We identify $\hat{\boldsymbol{\theta}}$ by maximising the likelihood:

$$\hat{\boldsymbol{\theta}} = \text{argmax}\{p(\mathbf{y})_{\boldsymbol{\theta}}\} \tag{38}$$

Let us reason about out-of-sample $\mathbf{f}_*$, assuming that we now have the BLUP-estimated parameters ($\hat{\boldsymbol{\theta}}$):

$$\begin{bmatrix} \mathbf{f} \\ \mathbf{f}_* \end{bmatrix} = \mathcal{N}\left(\begin{bmatrix} \mathbf{m}_{\hat{\boldsymbol{\theta}}} \\ \mathbf{m}_{*\hat{\boldsymbol{\theta}}} \end{bmatrix}; \begin{bmatrix} k(\mathbf{X}, \mathbf{X})_{\hat{\boldsymbol{\theta}}} & k(\mathbf{X}, \mathbf{X}_*)_{\hat{\boldsymbol{\theta}}} \\ k(\mathbf{X}_*, \mathbf{X})_{\hat{\boldsymbol{\theta}}} & k(\mathbf{X}_*, \mathbf{X}_*)_{\hat{\boldsymbol{\theta}}} \end{bmatrix} \right). \tag{39}$$

We want to know $\mathbf{y}_*$, it seems reasonable (argue better, maybe mentioning that this is also a BLUP estimation) to define (noise-free prediction according to [20])

$$\mathbf{y}_* \coloneqq \text{E}[\mathbf{f}_*|\mathbf{y}]_{\hat{\boldsymbol{\theta}}}. \tag{40}$$

We can show [20] that (note that we omit $\hat{\boldsymbol{\theta}}$ for reading purposes):

$$\mathbf{f}_*|\mathbf{f} \sim \mathcal{N}\left(\mathbf{m}_* + k(\mathbf{X}_*, \mathbf{X})k(\mathbf{X}, \mathbf{X})^{-1}(\mathbf{f} - \mathbf{m});\; k(\mathbf{X}_*, \mathbf{X}_*) - k(\mathbf{X}_*, \mathbf{X})k(\mathbf{X}, \mathbf{X})^{-1}k(\mathbf{X}, \mathbf{X}_*)\right). \tag{41}$$

Therefore (needs some steps to prove),

$$\mathbf{y}_* = \mathbf{m}_* + k(\mathbf{X}_*, \mathbf{X})k(\mathbf{X}, \mathbf{X})^{-1}(\mathbf{y} - \mathbf{m}). \tag{42}$$

In our case,

$$\mathbf{m} = \mathbf{W}\boldsymbol{\alpha} + \mathbf{g}\beta_G + \mathbf{E}\boldsymbol{\gamma}, \tag{43}$$

$$k(\mathbf{X}, \mathbf{X}) = \mathbf{K} = \sigma^2_{GxE}(\mathbf{g} \odot \mathbf{E})(\mathbf{g} \odot \mathbf{E})^T + \sigma^2_{re}\mathbf{R} \odot \mathbf{E}\mathbf{E}^T + \sigma^2_n\mathbf{I}. \tag{44}$$

Note that

$$k(\mathbf{X}_*, \mathbf{X}) = \mathbf{K}_{(*,.)} = \sigma^2_{GxE}(\mathbf{g}_* \odot \mathbf{E}_*)(\mathbf{g} \odot \mathbf{E})^T + \sigma^2_{re}\mathbf{R}_{(*,.)} \odot \mathbf{E}_*\mathbf{E}^T + \sigma^2_n\delta(\mathbf{X}_*, \mathbf{X}), \tag{45}$$

where δ is the Dirac distribution (0 when $i \neq j$ and 1 when $i = j$).

Therefore,

$$\mathbf{y}_* = \mathbf{W}_*\boldsymbol{\alpha} + \mathbf{g}_*\beta_G + \mathbf{E}_*\boldsymbol{\gamma} + k(\mathbf{X}_*, \mathbf{X})k(\mathbf{X}, \mathbf{X})^{-1}(\mathbf{y} - \mathbf{W}\boldsymbol{\alpha} + \mathbf{g}\beta_G + \mathbf{E}\boldsymbol{\gamma}) \tag{46}$$

Now, for each individual, we consider its environmental profile $\mathbf{e}_{*,i}$ (the ith corresponding row from $\mathbf{E}_*$), and estimate $y_{*,i}(\text{ref})$ when setting $g_{*,i} = 0$ and then $y_{*,i}(\text{alt})$ when setting $g_{*,i} = 1$ and obtain the corresponding allelic effect $\beta^*_{GxE,i}$ as the difference, normalised by a constant (based on p=MAF):

$$\beta^*_i = \frac{1}{\sqrt{2p(1-p)}}\,(y_{*,i}(\text{alt}) - y_{*,i}(\text{ref})) \tag{47}$$

For all samples, we consider the vectorial form:

$$\boldsymbol{\beta}^* = \frac{1}{\sqrt{2p(1-p)}}\,(\mathbf{y}_*(\text{alt}) - \mathbf{y}_*(\text{ref})), \tag{48}$$

where:

$$\mathbf{y}_*(\text{alt}) = \mathbf{y}_*(\mathbf{g}_* = \mathbf{1}) = \mathbf{m}_*(\mathbf{g}_* = \mathbf{1}) + \mathbf{K}_{(*,.)}(\mathbf{g}_* = \mathbf{1})\ \mathbf{K}^{-1}(\mathbf{y} - \mathbf{m}) \tag{49}$$

and

$$\mathbf{y}_*(\text{ref}) = \mathbf{y}_*(\mathbf{g}_* = \mathbf{0}) = \mathbf{m}_*(\mathbf{g}_* = \mathbf{0}) + \mathbf{K}_{(*,.)}(\mathbf{g}_* = \mathbf{0})\ \mathbf{K}^{-1}(\mathbf{y} - \mathbf{m}). \tag{50}$$

Let us set:

$$\mathbf{v} = \mathbf{K}^{-1}(\mathbf{y} - \mathbf{m}). \tag{51}$$

Next, we note that:

$$\mathbf{m}_*(\mathbf{g}_* = \mathbf{1}) - \mathbf{m}_*(\mathbf{g}_* = \mathbf{0}) = \mathbf{W}_*\boldsymbol{\alpha} + \mathbf{1}\beta_G + \mathbf{E}_*\boldsymbol{\gamma} - (\mathbf{W}_*\boldsymbol{\alpha} + \mathbf{E}_*\boldsymbol{\gamma}) = \mathbf{1}\beta_G. \tag{52}$$

Moreover,

$$\begin{aligned}
\mathbf{K}_{(*,.)}(\mathbf{g}_* = \mathbf{1}) - \mathbf{K}_{(*,.)}(\mathbf{g}_* = \mathbf{0}) = \\
\sigma^2_{GxE}\mathbf{E}_*(\mathbf{g} \odot \mathbf{E})^T + \sigma^2_{re}\mathbf{R}_{(*,.)} \odot \mathbf{E}_*\mathbf{E}^T + \sigma^2_n\mathbf{I} + \\
-(\sigma^2_{re}\mathbf{R}_{(*,.)} \odot \mathbf{E}_*\mathbf{E}^T + \sigma^2_n\mathbf{I}) = \\
\sigma^2_{GxE}\mathbf{E}_*(\mathbf{g} \odot \mathbf{E})^T
\end{aligned} \tag{53}$$

Thus,

$$\mathbf{y}_*(\text{alt}) - \mathbf{y}_*(\text{ref}) = \mathbf{1}\beta_G + \sigma^2_{GxE}\mathbf{E}_*(\mathbf{g} \odot \mathbf{E})^T\mathbf{v} \tag{54}$$

And finally, by substituting eq. (54) in eq. (48):

$$\boldsymbol{\beta}^* = \frac{1}{\sqrt{2p(1-p)}}\,\{\underbrace{\mathbf{1}\beta_G}_{\boldsymbol{\beta_{G^*}}} + \underbrace{\sigma^2_{GxE}\mathbf{E}_*(\mathbf{g} \odot \mathbf{E})^T\mathbf{K}^{-1}(\mathbf{y} - \mathbf{W}\boldsymbol{\alpha} - \mathbf{g}\beta_G - \mathbf{E}\boldsymbol{\gamma})}_{\boldsymbol{\beta_{GxE^*}}}\}, \tag{55}$$

where $\mathbf{K}$ is as defined in eq. (44).

References

[1] Julie Jerber, Daniel D Seaton, Anna SE Cuomo, Natsuhiko Kumasaka, James Haldane, Juliette Steer, Minal Patel, Daniel Pearce, Malin Andersson, Marc Jan Bonder, et al. Population-scale single-cell rna-seq profiling across dopaminergic neuron differentiation. *Nature genetics*, 53(3):304–312, 2021.

[2] Anna SE Cuomo, Daniel D Seaton, Davis J McCarthy, Iker Martinez, Marc Jan Bonder, Jose Garcia-Bernardo, Shradha Amatya, Pedro Madrigal, Abigail Isaacson, Florian Buettner, et al. Single-cell rna-sequencing of differentiating ips cells reveals dynamic genetic effects on gene expression. *Nature communications*, 11(1):1–14, 2020.

[3] Timothy A Myers, Stephen J Chanock, and Mitchell J Machiela. Ldlinkr: an r package for rapidly calculating linkage disequilibrium statistics in diverse populations. *Frontiers in genetics*, 11:157, 2020.

[4] Valentine Svensson, Adam Gayoso, Nir Yosef, and Lior Pachter. Interpretable factor models of single-cell rna-seq via variational autoencoders. *Bioinformatics*, 36(11):3418–3421, 2020.

[5] Davide Risso, Fanny Perraudeau, Svetlana Gribkova, Sandrine Dudoit, and Jean-Philippe Vert. A general and flexible method for signal extraction from single-cell rna-seq data. *Nature communications*, 9(1):1–17, 2018.

[6] Rachel Moore, Francesco Paolo Casale, Marc Jan Bonder, Danilo Horta, Lude Franke, Inês Barroso, and Oliver Stegle. A linear mixed-model approach to study multivariate gene–environment interactions. *Nature genetics*, 51(1):180–186, 2019.

[7] Gabriel E Hoffman. Correcting for population structure and kinship using the linear mixed model: theory and extensions. *PloS one*, 8(10):e75707, 2013.

[8] Nicoló Fusi, Oliver Stegle, and Neil D Lawrence. Joint modelling of confounding factors and prominent genetic regulators provides increased accuracy in genetical genomics studies. *PLoS Comput Biol*, 8(1):e1002330, 2012.

[9] David Heckerman, Deepti Gurdasani, Carl Kadie, Cristina Pomilla, Tommy Carstensen, Hilary Martin, Kenneth Ekoru, Rebecca N Nsubuga, Gerald Ssenyomo, Anatoli Kamali, et al. Linear mixed model for heritability estimation that explicitly addresses environmental variation. *Proceedings of the National Academy of Sciences*, 113(27):7377–7382, 2016.

[10] Ricard Argelaguet, Britta Velten, Damien Arnol, Sascha Dietrich, Thorsten Zenz, John C Marioni, Florian Buettner, Wolfgang Huber, and Oliver Stegle. Multi-omics factor analysis—a framework for unsupervised integration of multi-omics data sets. *Molecular systems biology*, 14(6), 2018.

[11] C Radhakrishna Rao. Large sample tests of statistical hypotheses concerning several parameters with applications to problems of estimation. In *Mathematical Proceedings of the Cambridge Philosophical Society*, volume 44, pages 50–57. Cambridge University Press, 1948.

[12] Christoph Lippert, Jennifer Listgarten, Ying Liu, Carl M Kadie, Robert I Davidson, and David Heckerman. Fast linear mixed models for genome-wide association studies. *Nature methods*, 8(10):833, 2011.

[13] Christoph Lippert, Jing Xiang, Danilo Horta, Christian Widmer, Carl Kadie, David Heckerman, and Jennifer Listgarten. Greater power and computational efficiency for kernel-based association testing of sets of genetic variants. *Bioinformatics*, 30(22):3206–3214, 2014.

[14] Michael C Wu, Seunggeun Lee, Tianxi Cai, Yun Li, Michael Boehnke, and Xihong Lin. Rare-variant association testing for sequencing data with the sequence kernel association test. *The American Journal of Human Genetics*, 89(1):82–93, 2011.

[15] Robert B Davies. Algorithm as 155: The distribution of a linear combination of χ 2 random variables. *Journal of the Royal Statistical Society. Series C (Applied Statistics)*, 29(3):323–333, 1980.

[16] Huan Liu, Yongqiang Tang, and Hao Helen Zhang. A new chi-square approximation to the distribution of non-negative definite quadratic forms in non-central normal variables. *Computational Statistics & Data Analysis*, 53(4):853–856, 2009.

[17] Seunggeun Lee, Michael C Wu, and Xihong Lin. Optimal tests for rare variant effects in sequencing association studies. *Biostatistics*, 13(4):762–775, 2012.

[18] Pierre Duchesne and Pierre Lafaye De Micheaux. Computing the distribution of quadratic forms: Further comparisons between the liu–tang–zhang approximation and exact methods. *Computational Statistics & Data Analysis*, 54(4):858–862, 2010.

[19] George K Robinson et al. That blup is a good thing: the estimation of random effects. *Statistical science*, 6(1):15–32, 1991.

[20] Carl Edward Rasmussen. Gaussian processes in machine learning. In *Summer school on machine learning*, pages 63–71. Springer, 2003.
