# Supplementary material for: CellRegMap: a statistical framework for mapping context‐specific regulatory variants using scRNA‐seq
Source: Mol Syst Biol. 2022 Aug 16;18(8):e10663. doi: 10.15252/msb.202110663 (PMC9380406; doi:10.15252/msb.202110663)
Supplement: Supplementary file 2 — Expanded View Figures PDF [file MSB-18-e10663-s007.pdf]

Expanded View Figures

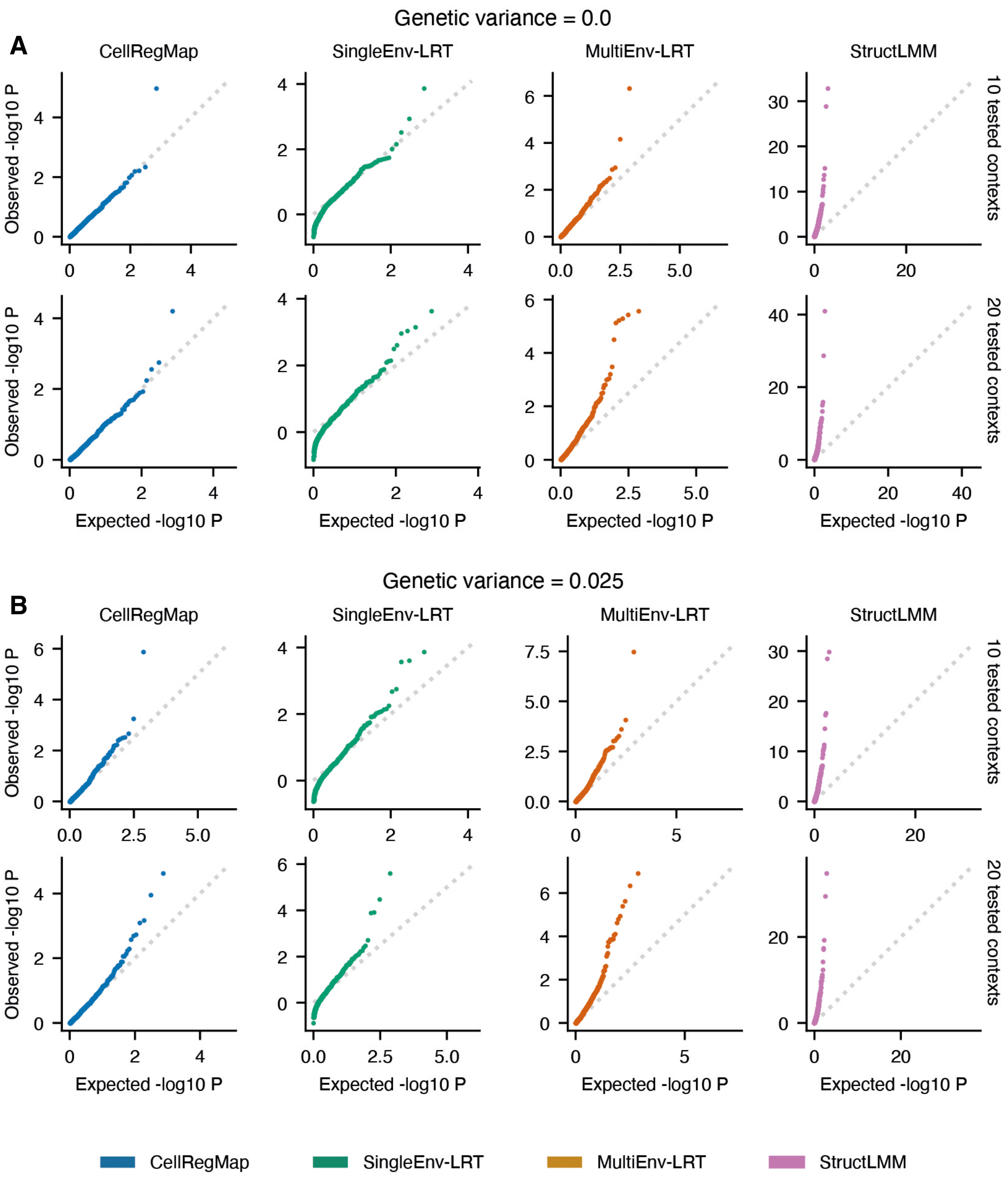

Figure EV1. Assessment of statistical calibration based on simulated data.

Semi-empirical simulated data as described in Materials and Methods.

A, B QQ plots displaying expected versus observed negative log *P*-values, considering different levels of persistent genetic variance (panel A versus panel B). Rows represent different numbers of tested context variables (10 or 20). All models control for the same number of background contexts as tested (additive effects of environmental context and context-repeat-structure interaction). Shown are CellRegMap (blue), a fixed-effect likelihood-ratio-test for single contexts (SingleEnv-LRT, min. *P*-value across all contexts, Bonferroni-adjusted for the number of tested contexts; green), a multicontext fixed-effect test (MultiEnv-LRT; orange) and StructLMM (pink; Moore *et al*, 2019). The latter model is related to CellRegMap but does not account for the repeat structure present in single-cell data. All other models include a random effect component that captures the donor repeat structure across sampled cells. MultiEnv-LRT and StructLMM do not achieve calibrated test statistics. Note that the QQ plots for CellRegMap, SingleEnv-LRT and StructLMM from the first row in panel A (genetic variance = 0 and 10 contexts tested) are reused in Fig 2A.

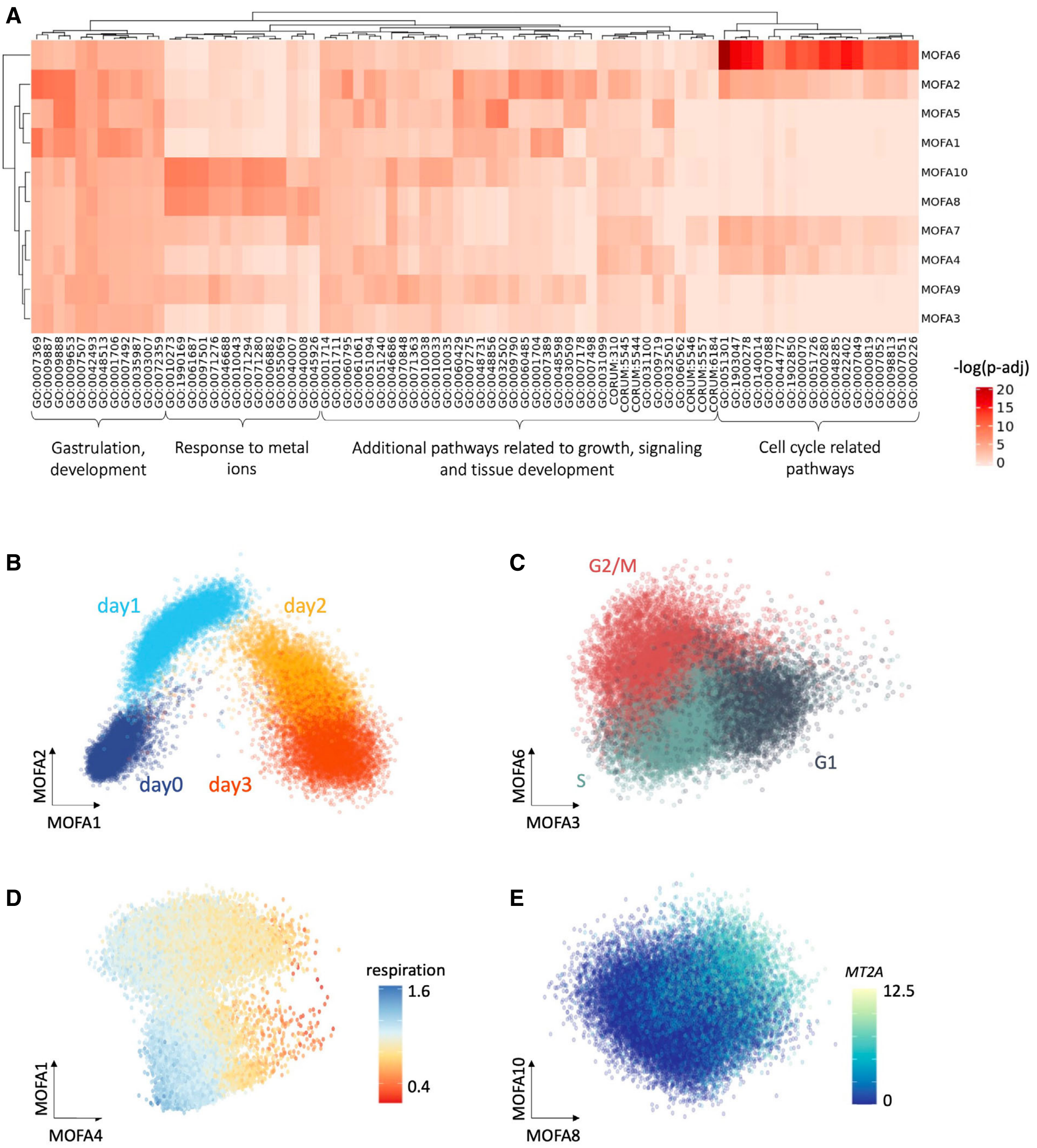

Figure EV2. Annotation of the MOFA factors from the endoderm differentiation study.

Data ref: Cuomo *et al* (2020b).

A Heatmap displaying negative log *P*-values from GO enrichment analyses based on the absolute values of the loadings of individual MOFA factors (Materials and Methods).

B Scatter plot of MOFA factors 1 & 2, with color corresponding to the time point of collection (day 0,1,2 & 3 of endoderm differentiation).

C Scatter plot of MOFA factors 3 & 6, colored by estimated cell cycle phase (G1, G2/M, S; estimated using Seurat).

D Scatter plot of MOFA factors 1 & 4 capturing respiration.

E Scatter plot of MOFA factors 8 & 10, capturing a signature linked to response to metal ions, colored by expression of gene with top loadings, *MT2A* (Materials and Methods). Note that the scatter plots of MOFA factors in panels (B, C and D) are reused as reference in Fig 3.

© 2022 The Authors

Figure EV3. Additional examples of GxC interactions identified in the endoderm differentiation study using CellRegMap.

Data ref: Cuomo *et al* (2020b).

A Analogous to main text Fig 3B (middle), examples of dynamic eQTL that vary continuously along MOFA 1 only.

B Similar to the main text Fig 3B (right), additional examples of eQTL with GxC effects associated with MOFA factors 1 & 2.

C Additional examples as in the main text to Fig 3C, displaying GxC effects across MOFA factors 3 & 6 (capturing cell cycle).

D Additional examples as in the main text Fig 3C, displaying GxC effects along MOFA factors 4 and 1 (capturing cell respiration).

E Examples of eQTL with GxC effects that are associated with cell states captured by MOFA 8 & 10, related to response to metal ions (c.f. Fig EV2).

© 2022 The Authors

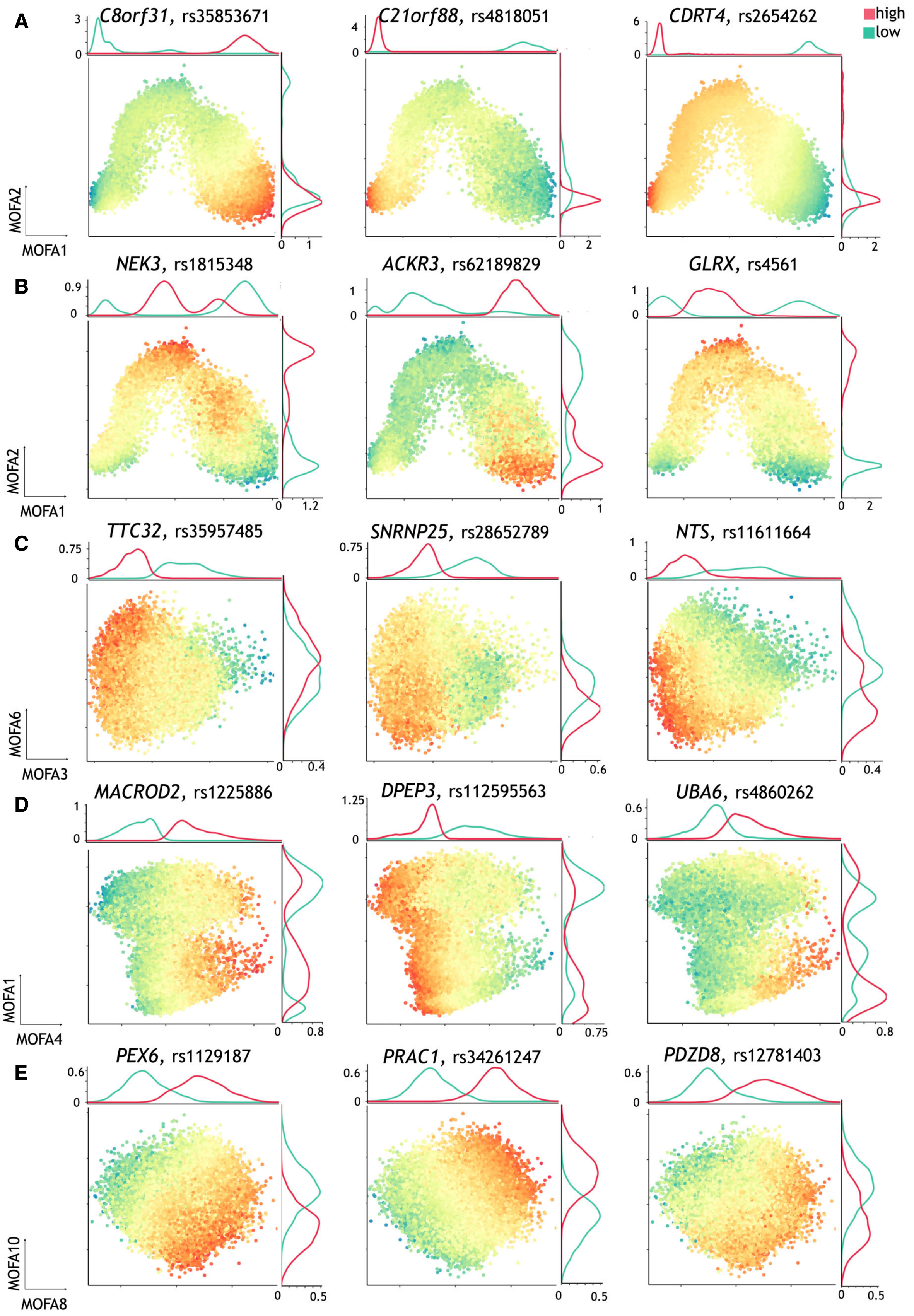

Figure EV3.

© 2022 The Authors

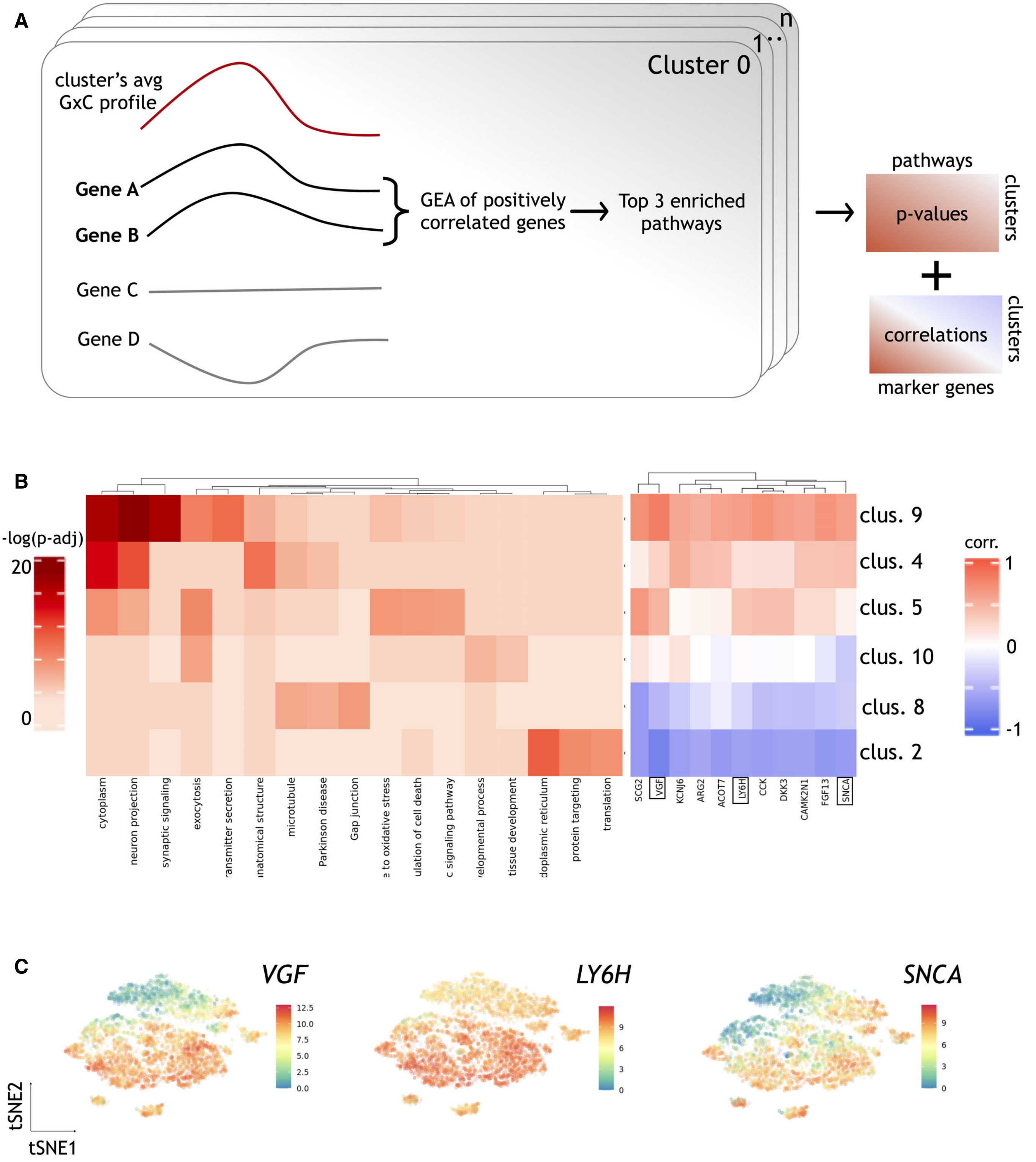
A
Cluster 0
1
n
cluster's avg GxC profile
Gene A
Gene B
Gene C
Gene D
GEA of positively correlated genes
Top 3 enriched pathways
pathways
p-values
clusters
correlations
clusters
marker genes
B
-log(p-adj)
20
0
clus. 9
clus. 4
clus. 5
clus. 10
clus. 8
clus. 2
corr.
1
0
-1
cytoplasm
neuron projection
synaptic signaling
exocytosis
microtubule
Parkinson disease
Gap junction
tissue development
protein targeting
translation
SCG2
VGF
KCNJ6
ARG2
ACOT7
LY6H
CCK
DKK3
CAMK2N1
FGF13
SNCA
C
VGF
LY6H
SNCA
tSNE2
tSNE1

Figure EV4.

Figure EV4. Annotation of GxC clusters based on gene set enrichment in the neuronal differentiation data.

Data ref: Jerber *et al* (2021b).

A Illustration of the clustering approach to identify patterns of GxC interactions. Briefly, for each cluster we considered genes whose single-cell profiles were positively correlated with the average GxC allelic effect profile. Genes with positive correlation (Pearson's correlation > 0.4) were considered for gene enrichment analysis using gprofiler, and up to 3 top significant (adjusted *P*-values < 0.05) terms were considered per cluster (Materials and Methods).

B Left: Enrichment results for the 6 clusters in Fig 4C–H. Shown is a heatmap of adjusted negative log *P*-values of enrichment results obtained by gprofiler. Right: Correlation coefficients between the GxC allelic effect profile and expression level for selected literature-curated dopaminergic neuron markers. For each cluster, the correlation coefficient between the expression level of the respective marker gene and the aggregate cluster allelic effect size profile is shown.

C Expression profiles across pseudocells for three selected neuronal marker genes highlighted in the right panel of (B). tSNE plots, colored by expression level of the three genes.

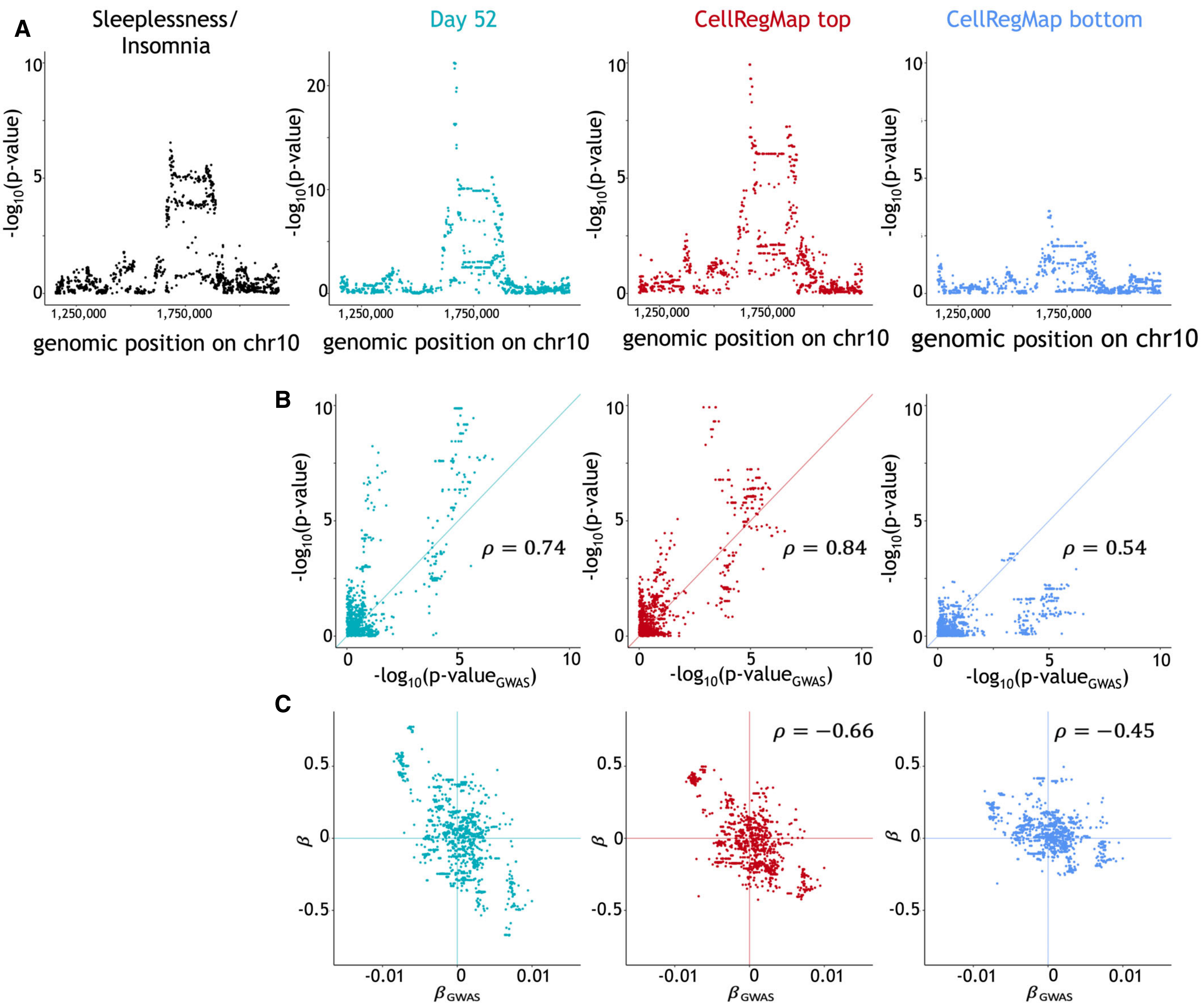

Figure EV5.

 © 2022 The Authors

Figure EV5. CellRegMap allows to characterize human disease variants colocalized with eQTL in dopaminergic neurons.

GxC profile at rs1972183 for *SLC35E2* identifies cellular population linked to GWAS variant for insomnia/sleeplessness. Data ref: Jerber *et al* (2021b), when considering cells identified as dopaminergic neurons only (Materials and Methods). Columns represent all cells at day 52 (aqua), cell population that CellRegMap identifies to have strongest effects (red, top 30% quantile from β_{GxC}) and weakest (blue, bottom 30% quantile).

A Manhattan plots for the relevant genomic region. From left to right: GWAS for Sleeplessness Insomnia, eQTL using aggregate expression estimates across all day 52 (untreated), eQTL Manhattan plot when considering day 52 cells in the top quantile, eQTL Manhattan plot when considering day 52 cells in the bottom quantile. Note that the latter two Manhattan plots for the top and bottom quantiles using CellRegMap are reused in Fig 5D.

B, C Comparison between GWAS signal and eQTL signal, considering alternative traits based on the cell populations as in (A). (B) Scatter plots of negative log *P*-values from GWAS (*x*-axis) versus eQTL (*y*-axis). (C) As in (B), displaying effect size estimates.
