# Supplementary material for: CellRegMap: a statistical framework for mapping context‐specific regulatory variants using scRNA‐seq
Source: Mol Syst Biol. 2022 Aug 16;18(8):e10663. doi: 10.15252/msb.202110663 (PMC9380406; doi:10.15252/msb.202110663)
Supplement: Supplementary file 8 [file MSB-18-e10663-s003.pdf]

molecular systems biology

CellRegMap: A statistical framework for mapping context-specific regulatory variants using scRNA-seq

Anna Cuomo, Tobias Heinen, Danai Vagiaki, Danilo Horta, John Marioni, and Oliver Stegle
DOI: 10.15252/msb.202110663

Corresponding authors: Oliver Stegle (o.stegle@dkfz-heidelberg.de) , Anna Cuomo (a.cuomo@garvan.org.au)

Review Timeline:

Submission Date:	31st Aug 21
Editorial Decision:	18th Oct 21
Revision Received:	1st May 22
Editorial Decision:	8th Jun 22
Revision Received:	28th Jun 22
Accepted:	1st Jul 22

Editor: Jingyi Hou

Transaction Report:

(Note: With the exception of the correction of typographical or spelling errors that could be a source of ambiguity, letters and reports are not edited. Depending on transfer agreements, referee reports obtained elsewhere may or may not be included in this compilation. Referee reports are anonymous unless the Referee chooses to sign their reports.)

18th Oct 2021

Manuscript Number: MSB-2021-10663
Title: CellRegMap: A statistical framework for mapping context-specific regulatory variants using scRNA-seq
Author: Anna Cuomo
Tobias Heinen
Danai Vagiaki
Danilo Horta
John Marioni
Oliver Stegle

Dear Dr Stegle,

Thank you for submitting your work to Molecular Systems Biology. We have now heard back from two of the three reviewers who agreed to evaluate your manuscript. As you will see from the reports below, the reviewers acknowledge the potential interest of the study. They raise however a series of concerns, which we would ask you to address in a major revision.

Since the reviewers' recommendations are rather clear, there is no need to reiterate all the points listed below. All issues raised by the reviewers need to be satisfactorily addressed. As you may already know, our editorial policy allows in principle a single round of major revision and it is therefore essential to provide responses to the reviewers' comments that are as complete as possible.

On a more editorial level, we would ask you to address the following issues:

- Please provide a .docx formatted version of the manuscript text (including legends for main figures, EV figures and tables). Please make sure that the changes are highlighted to be clearly visible.

- Please provide individual production quality figure files as .eps, .tif, .jpg (one file per figure).

-Please provide a .docx formatted letter INCLUDING the reviewers' reports and your detailed point-by-point responses to their comments. As part of the EMBO Press transparent editorial process, the point-by-point response is part of the Review Process File (RPF), which will be published alongside your paper.

-Please note that all corresponding authors are required to supply an ORCID ID for their name upon submission of a revised manuscript.

-We replaced Supplementary Information with Expanded View (EV) Figures and Tables that are collapsible/expandable online (see examples in http://msb.embopress.org/content/11/6/812). A maximum of 5 EV Figures can be typeset. EV Figures should be cited as 'Figure EV1, Figure EV2" etc... in the text and their respective legends should be included in the main text after the legends of regular figures.

Additional Tables/Datasets should be labeled and referred to as Table EV1, Dataset EV1, etc. Legends have to be provided in a separate tab in case of .xls files. Alternatively, the legend can be supplied as a separate text file (README) and zipped together with the Table/Dataset file.

For the figures and tables that you do NOT wish to display as Expanded View figures, they should be bundled together with their legends in a single PDF file called *Appendix*, which should start with a short Table of Content. Each legend should be below the corresponding Figure/Table in the Appendix. Appendix figures and tables should be referred to in the main text as: "Appendix Figure S1, Appendix Figure S2, Appendix Table S1" etc. See detailed instructions regarding expanded view here: https://www.embopress.org/page/journal/17444292/authorguide#expandedview.

-Before submitting your revision, primary datasets (and computer code, where appropriate) produced in this study need to be deposited in an appropriate public database (see https://www.embopress.org/page/journal/17444292/authorguide#dataavailability).

Please remember to provide a reviewer password if the datasets are not yet public.

The accession numbers and database should be listed in a formal "Data Availability " section (placed after Materials & Method) that follows the model below (see also https://www.embopress.org/page/journal/17444292/authorguide#dataavailability). Please note that the Data Availability Section is restricted to new primary data that are part of this study.

Data availability

The datasets (and computer code) produced in this study are available in the following databases:

- RNA-Seq data: Gene Expression Omnibus GSE46843 (https://www.ncbi.nlm.nih.gov/geo/query/acc.cgi?acc=GSE46843)
- [data type]: [name of the resource] [accession number/identifier/doi] ([URL or identifiers.org/DATABASE:ACCESSION])

*** Note - All links should resolve to a page where the data can be accessed. ***

- We would encourage you to include the source data for figure panels that show essential quantitative information. Additional information on source data and instruction on how to label the files are available at <
https://www.embopress.org/page/journal/17444292/authorguide#sourcedata
>.

- All Materials and Methods need to be described in the main text. Since the study presents a new approach, we would kindly ask you to use 'Structured Methods', our new Materials and Methods format. According to this format, the Material and Methods section should include a Reagents and Tools Table (listing key reagents, experimental models, software and relevant equipment and including their sources and relevant identifiers) followed by a Methods and Protocols section in which we encourage the authors to describe their methods using a step-by-step protocol format with bullet points, to facilitate the adoption of the methodologies across labs. More information on how to adhere to this format as well as downloadable templates (.doc or .xls) for the Reagents and Tools Table can be found in our author guidelines: <
https://www.embopress.org/page/journal/17444292/authorguide#researcharticleguide>. An example of a Method paper with Structured Methods can be found here: .

-Regarding data quantification:
Please ensure to specify the name of the statistical test used to generate error bars and P values, the number (n) of independent experiments (please specify technical or biological replicates) underlying each data point and the test used to calculate p-values in each figure legend. Discussion of statistical methodology can be reported in the materials and methods section, but figure legends should contain a basic description of n, P and the test applied.
Graphs must include a description of the bars and the error bars (s.d., s.e.m.).
Please also include scale bars in all microscopy images.

- Please provide a "standfirst text" summarizing the study in one or two sentences (approximately 250 characters, including space), three to four "bullet points" highlighting the main findings and a "synopsis image" (550px width and 400-600 px height, PNG format) to highlight the paper on our homepage.
Here are a couple of examples:
https://www.embopress.org/doi/10.15252/msb.20199356
https://www.embopress.org/doi/10.15252/msb.20209475
https://www.embopress.org/doi/10.15252/msb.209495

When you resubmit your manuscript, please download our CHECKLIST (http://bit.ly/EMBOPressAuthorChecklist) and include the completed form in your submission.
Please note that the Author Checklist will be published alongside the paper as part of the transparent process (https://www.embopress.org/page/journal/17444292/authorguide#transparentprocess).

If you feel you can satisfactorily deal with these points and those listed by the referees, you may wish to submit a revised version of your manuscript. Please attach a covering letter giving details of the way in which you have handled each of the points raised by the referees. A revised manuscript will be once again subject to review and you probably understand that we can give you no guarantee at this stage that the eventual outcome will be favorable.

Kind regards,

Jingyi

Jingyi Hou
Editor
Molecular Systems Biology

Reviewer #1:

Cuomo et al. present CellRegMap, a method for improved mapping of context-specific eQTLs from single cell data. These types of methods are much needed in the community as the population-scale single-cell data sets are growing, and most existing methods are not ideally suited for single cells. Here, they expand an existing linear mixed model approach to account for multiple cells from the same individual, and allow joint analysis of multiple contexts derived from PCA or factor analysis approaches of single cell data. They demonstrate improved performance over alternative methods and apply their method to two existing data sets. This is a valuable method and a well-written manuscript. I have only relatively minor suggestions, primarily to provide a more transparent analysis of circumstances where the method might start to underperform or even fail.

It was unclear to me what is the interpretation of the direction of the effect of the interaction term, and if there's a biological interpretation in the switch in direction which seems to happen for most of the example genes.

In the SLC35E2 example, it seems that the effect size is higher in cell populations where the expression of the gene is higher (SFig 5.1). This raises a more general question: Is it possible that some of the GxC effect is due to expression often hitting 0 when gene expression is low (forming effectively a lower bound of detection due to sparsity of the sc data)? Thus, one wouldn't be able to detect an eQTL effect in cells where expression is low, whereas in cells with higher expression it can be seen? While

this could reflect real biology, it could be also a study design artefact of a given expression level, and a higher number of reads or otherwise a more sensitive detection of the gene's expression would actually uncover a similar eQTL effect. This should be explored in some detail by observing if eQTL effects are typically low when gene expression is low (a pattern not seen in bulk data across tissues, see GTEx Consortium 2019). Downsampling or simulations could be used to address this further.

A related question: it is mentioned that the quantile normalization of the expression data is quite harsh for lower expressed genes, as one would expect.

I would assume that the method performs best when the interaction context distribution is approximately normal. What happens if there are strong outliers, very tight clusters, etc? Should e.g. MOFA factors (or whatever is used instead) be normalized somehow?

A minor point: "This analysis confirmed the expected difference in effect sizes (Fig. 5e,f), but also highlighted subtle differences in the cis eQTL mapping profile for each of these traits (Fig. 5d). Notably, the trait associated with the top quantile of increased allelic effects also yielded higher evidence for co-localization with the disease GWAS signal (Supplementary Fig. 5.2)." - I don't quite understand what the "subtle differences" refers to. Isn't the improved evidence for colocalization just an outcome of better power, with more variants having real signal with meaningful correlation? The patterns of CellRegMap top/bottom in SFig 5.2 don't really look qualitatively different to me (in terms of the association landscape or p-value correlations).

It would be good to mention that discovering the cell population where a given GWAS eQTL has its strongest effect does not necessarily mean that this is The Cell State that is driving the disease association. It could be that a genetic effect of smaller magnitude in a different cell type is what actually matters.

Speed seems to be a limiting factor here. I'd like to see an expansion of SFig 1.1 to more realistic sample sizes - QTL mapping with 25 individuals is not a great idea to begin with, and even the real data used here has much higher sample sizes than presented here.

I would encourage the authors to add more material in the CellRegMap website to provide more guidance on the important practicalities regarding thresholds, pseudocell calculation (when is it needed exactly?), normalization, multiple testing correction, etc.

Reviewer #3:

Summary:
The authors create a tool to estimate the impact of genotype on single cell RNA profiles, called CellRegMap. This tool is based on a linear model that takes into account: 1) persistent genetic effects across all cells; 2) cellular context as defined from a co-variance structure obtained from e.g. PCA or MOFA; 3) interaction between genotype and cellular context (which also includes environmental effects on the individuals); 4) relatedness of cells profiled from the same individual.

This tool has the following advantages compared to existing approaches:
• Cellular contexts (cell types / states / differentiation pseudotimes) do not have to be discretized, which more accurately captures variation of gene expression
• Interactions between cellular contexts and genotype are tested, which allows for the discovery of e.g. loci with different impacts on expression in different cell types
• Different cellular contexts are modelled together, which allows to identify shared effects

In general, the paper makes a good impression; most parts of it are very clearly written (exception see below) and the method as such is very solid. Needless to say, a (good) method to detect eQTL in single cell data is really needed. I think the way to take subtle cell subtypes into account is elegant. Further, the code on GitHub is very well structured and documented. Further, the testing was done on scRNA-seq data from two different platforms (Smart-seq2 & 10X).
While I have no concerns regarding the method as such, its validation needs to be improved (see below). Also, in their submission the authors restricted the application of CellRegMap to a priori known eQTL. There might be computational and statistical (multiple testing problem) reasons for such a restriction. However, the authors do not even attempt to discover new eQTL, although their method provides great promise to detect context-specific eQTL that might remain undetected in an unspecific we-gonna-merge-everything approach. Further, the authors need to characterize the genes that benefit from explicit modelling of the context. What makes them different? (see below)

This is a pure methods paper. I.e. the editor needs to consider if this submission fits the scope of MSB. However, I do see a need for such method.

Major Points:
1. Simulations are based on the model underlying CellRegMap. Obviously, this will favor CellRegMap as the best method to detect the eQTL. This is kind of circular reasoning. I do appreciate that the simulations without interaction effects contribute, because they show that the method does not produce excessively many false positives. Further, those simulations do show that the method works under its assumptions. However, the simulations need to be more realistic. (1) Real genotypes should be used to capture realistic distributions of MAFs and LD. (2) Low read counts and dropouts should be simulated. scRNA-seq data suffers from many specific problems such as dependency on library size/capturing efficiency, etc. There are existing tools such as splatPop (http://www.bioconductor.org/packages/devel/bioc/vignettes/splatter/inst/doc/splatPop.html) that should be used. Even better would be a methods comparison on real data. This is of course much harder, since the ground truth is not known. One possible way would be to quantify how much the observed eQTL colocalize with relevant genes and regulatory loci from previous GWAS or eQTL studies, or based on annotation. The authors already touched on this concept in the final results paragraphs.
2. The authors state that there are already established approaches for multi-tissue eQTL analysis. I don't know whether specific methods for single-cell-eQTL analysis exist already. Either way, I would like to see how their approach fares compared to these "established" methods, both in the simulation and the real data. The only comparison they performed was on the simulated methods, where they also applied a simpler model that could not handle multiple genetic contexts as well as their CellRegMap (Figure 2). This is not surprising, because this simpler model only allowed for a single context by design. So this comparison is not really a fair one, especially because the simulated data were created according to the assumed model of CellRegMap (and with only 50 cells).
3. The current approach assumes a Gaussian normal distribution although it is community standard to assume a Negative Binomial and (sometimes) zero inflation. This concern is addressed in the Discussion section, but it would be relevant to show how applicable this model is to highly expressed vs lowly expressed target genes with a clear performance comparison in real scRNA-seq data. Supplementary Figure 2.2 shows that the power is almost twice as high when assuming a Negative Binomial as opposed to a Gaussian (compare panels b and d "Number of contexts with GxC"). Why do the authors not assume NB by default? How does the power depend on the expression level? It is likely that the Gaussian will fail especially for lowly expressed genes.
4. The way CellRegMap is used so far is only re-analyzing eQTL that were previously detected using traditional analyses (see above). Why is that? The runtimes don't seem so terribly bad that running it in an unbiased way would be completely prohibitive. It should be tested at least for some traits. Furthermore, the authors only state that "the computational complexity of CellRegMap scales linearly with the number of cells". A little more detail about computation times in the main text would be helpful to give readers an idea of which application would be feasible. Finally, I think the text in the Introduction and Abstract should be adapted to honestly reflect the fact that this method is not feasible for de novo eQTL detection in larger datasets. Since the methods used for locus-gene pair preselection do not take diverse contexts into account, one would still "fail to detect changes in allelic regulation across subtle cell subtypes" if the effect are not also visible on the global scale.
5. Better show with real data the benefit of using a continuous context as compared to discretizing the context. Do both on the same real data and compare the results. How much do you gain? Also: what about the reverse? What if the cell types are actually discrete, but the context is modelled as a continuous (linear) variable. What would you lose?
6. Which genes benefit most from modelling GxC interactions? There is one simple explanation: a gene is expressed in one context, but not in the other. In such a case it would be impossible to detect an eQTL in the context where the gene is not expressed. That example would represent a bona fide context-specific eQTL and the explanation would be really simple. How many of the context-specific GxC eQTL are of this type? Note that this is related to the question of an unbiased de novo screen for GxC eQTL. (Without that you wouldn't know unbiased fractions of eQTL of certain types.)
7. The impact of using other upstream analyses (PCA or ZINB-WaVe, instead of MOFA) on the model performance is not clear.

Minor Points:
8. Figure 1e: make explicit that the color indicates allelic effects. This is particularly important to distinguish the meaning of color from panel c in the same figure.
9. Page 4: "GxC interactions are modelled as an element-wise product between the expanded genotype vector g at a given locus and a GxC effect size vector beta_GxC ..." What does the index [1 ... N] in this equation refer to? Is this really an iteration over all cells? I thought iterating over the contexts would make more sense. But the first sentence in the paragraph suggests that the index refers to cells.
10. Figure 2d: Why does SingleEnv-Int still perform better when there are two nonzero GxC contributions? What if you have only one C with a real effect? Would CellRegMap overfit, i.e. would you get more false positives?
11. Page 7: "The model with 10 components yielded a substantially larger number GxC effects (322 versus 183, FDR<5%;" Did the FDR estimation account for the fact that the model with 10 components is testing ten times more hypotheses?
12. Figure 4: Why was t-SNE used here for the visualization instead of MOFA?
13. Page 9: "We tested for GxC effects at 1,374 SNP-gene pairs identified as eQTL in at least one of the three discrete cell populations in the primary analysis of the data ..." This entire paragraph is very difficult to read. Don't use fluffy terms like 'landscape of GxC effects'. Be explicit about what you are doing. Same with 'allelic effect pattern'. What is that? What 'pattern'? Just say clearly what the distance measure is based on. Say explicitly what you are clustering. I think you are clustering eQTL, but that should be made explicit. Why do you correlate allelic effects with gene expression? Why can't you directly sort by allelic effect? Please, re-write this paragraph and let it be prove-read by some external colleague.
14. Figure 4 c-h: The ordering of panels/clusters seems quite arbitrary. Why don't you use the same ordering as in panel b (i.e. by size)? Could be fewer examples.

15. Page 11: "CellRegMap allowed for fine-mapping a specific sub-populations ..." Don't use 'fine mapping' here, because it has a different meaning in statistical genetics.
16. Following sentence: check spelling and grammar.
17. Figure 5b (caption): Make explicit that these are the top and bottom quantiles of the 52 day untreated cells only (as opposed to top and bottom from all cells shown in the plot).

Dear Jingyi,

We would like to thank you and the reviewers for the detailed and constructive feedback on our manuscript, especially given the current circumstances, and apologise for the delay in submitting a revised manuscript. We have now comprehensively addressed the specific comments raised by the referees (see below). Briefly, the key changes of our manuscript are:

- **Improvement of simulation setup,** which is now based on real genotypes and real single-cell expression profiles. The revised simulation strategy also features a more realistic approach to simulating dropout rates and we consider larger numbers of donors and cells as expected in real data.

- **Implementation of a persistent genetic effect test**, which allows our approach to be used for discovery on a dataset where eQTL are not known. This change will increase the applicability of the model, enabling users to create an end-to-end workflow of variant discovery followed by GxC analysis.

- **Improvements to the usability of the software**, including more guidance on the usage of the package, with more detail on data preprocessing and the generation of input files. We have also refactored the code to make the functions as intuitive as possible and provide extensive documentation on the CellRegMap webpage.

- **Refined analysis and presentation of the results on the considered single-cell datasets,** including exploration of the relationship between genetic effect and gene expression dynamics, and characterisation of different eGenes.

A point-by-point response to the individual comments raised by the reviewers is provided below. Sections in the paper with substantial changes are highlighted in colour. Collectively, we feel that these changes have substantially strengthened our manuscript and we hope that our revised manuscript is now suitable for publication in *Molecular Systems Biology*.

On behalf of all authors,

Anna Cuomo and Oliver Stegle

Key:

- Reviewers' comments
- Our response

Reviewer #1:

Cuomo et al. present CellRegMap, a method for improved mapping of context-specific eQTLs from single cell data. These types of methods are much needed in the community as the population-scale single-cell data sets are growing, and most existing methods are not ideally suited for single cells. Here, they expand an existing linear mixed model approach to account for multiple cells from the same individual, and allow joint analysis of multiple contexts derived from PCA or factor analysis approaches of single cell data. They demonstrate improved performance over alternative methods and apply their method to two existing data sets. This is a valuable method and a well-written manuscript. I have only relatively minor suggestions, primarily to provide a more transparent analysis of circumstances where the method might start to underperform or even fail.

We thank the reviewer for their valuable feedback and have addressed their comments one-by-one below. In particular, we have:
1) expanded our simulation framework to more clearly assess the performance benefits of CellRegMap in realistic settings,
2) clarified processing steps that are required prior to running CellRegMap, both in terms of preprocessing of the single-cell raw data itself, but also in terms of processing steps to identify candidate eQTL variants to be tested for GxC within the CellRegMap framework, and
3) explored in more detail the biological and technical characteristics of genes that display GxC effects, particularly in the case of "opposite sign effects" across cells.

1. It was unclear to me what is the interpretation of the direction of the effect of the interaction term, and if there's a biological interpretation in the switch in direction which seems to happen for most of the example genes.

It is correct that the maps of genetic effect size estimates obtained from CellRegMap (*e.g.,* Figures 3 and 4) can in principle be used to interpret the specific form of GxC effects. A caveat is that these estimates as shown in the 2D plots in the paper do not include estimated uncertainties, and hence we recommend to consider them as qualitative evidence.

This applies in particular to the putative opposite effects the reviewer is referring to. Without taking the uncertainty estimates into account, the "visual interpretation" of possible opposite effects may be too optimistic. We also note that the CellRegMap GxC test does not formally assess the evidence for opposite effects, but only for whether the cellular environments are modulating genetic effects on expression.

Having said this, we agree that it is an interesting question to explore to what extent the output from CellRegMap can facilitate the discovery of opposite effects. To this end, we now provide summary results (**Supplementary Table 5**) of putative opposite effects identified by CellRegMap, which are indeed relatively frequent (21%, considering the neuronal differentiation data from Jerber et al. (Jerber et al. 2021)). We have also employed a classical approach, which is based on estimating genetic effects in the extreme strata of cells

identified by CellRegMap (top and bottom 20% of estimated betaGxC values). This baseline approach confirms 72% of the opposite effects identified (**Supplementary Fig. 9**). Of note, this replication strategy may be underpowered to detect true opposite effects as only 40% of the dataset is used, depending on the distribution of cells with different effect sizes. This is also evident from the observation that replicating opposite effects have higher portions of cells with different direction of effect (closer to a 50-50 split), while the non-replicating are characterised by a smaller magnitude of the GxC effects (estimated as the delta between top and bottom 10% of betaGxC values; **Supplementary Fig. 9**). We have included this analysis in the main text (page 11, paragraph 2 (excluding figure captions)).

2. In the SLC35E2 example, it seems that the effect size is higher in cell populations where the expression of the gene is higher (SFig 5.1). This raises a more general question: Is it possible that some of the GxC effect is due to expression often hitting 0 when gene expression is low (forming effectively a lower bound of detection due to sparsity of the sc data)? Thus, one wouldn't be able to detect an eQTL effect in cells where expression is low, whereas in cells with higher expression it can be seen? While this could reflect real biology, it could be also a study design artefact of a given expression level, and a higher number of reads or otherwise a more sensitive detection of the gene's expression would actually uncover a similar eQTL effect. This should be explored in some detail by observing if eQTL effects are typically low when gene expression is low (a pattern not seen in bulk data across tissues, see GTEx Consortium 2019). Downsampling or simulations could be used to address this further.

The reviewer raises an important point. We have now systematically assessed the relationship between the gene expression dynamics and variation in genetic effects (**Supplementary Fig. 6a**). First, we note that the number of genes where such an association exist is moderate (40 out of 322 GxC effects in the endoderm differentiation data, $R^2>0.2$), and includes both positive and negative associations at a similar proportion (24 positive correlations vs 16 negative ones). We also note that the strongest GxC effects identified tend to be independent of the expression dynamics (**Supplementary Fig. 6b**). Regarding possible causes for such associations, we have separately considered two relevant explanations.

First, as mentioned by the reviewer, true biological factors could underlie GxC effects while also being drivers of wide-spread expression changes. It is expected that major cell state transitions occurring *e.g.*, during cellular differentiation may manifest in both expression changes and changes in the regulatory landscape. Indeed, the majority of the 40 GxC effects with an expression association are strongly linked to the differentiation trajectory (24/40 have $R^2>0.2$ between expression and pseudotime, 36/40 have $R^2>0.2$ between GxC and pseudotime, **Supplementary Fig. 6c**), suggesting a biological cause.

Second, in principle there could be technical reasons whereby allelic effects may not be reliably estimated in a subset of cells with low expression. This could either result in false positive or false negative GxC effects. We have addressed both possibilities.

1. First, we extended the statistical calibration analysis on real data using permuted genotypes, and now consider genes with and without a gradient in expression separately. Critically, we observe that the model retains calibration in both cases,

thus ruling out technical factors as drivers of the observed GxC effects (**Supplementary Fig. 7**).

2. Second, using simulations, we considered the impact of expression level and expression variance on the power to detect genuine GxC effects. Briefly, varying either average expression level or variance of simulated expression profiles, we observed a modest increase in power to detect true signals for CellRegMap (blue) for higher expression mean and lower variance (**Supplementary Fig. 3**).

In sum, there are intrinsic relationships between expression level and the ability to pick up GxC effects. Such associations can be biological, and our data indicate that the moderate number of cases where such an association exists are driven by shared biological factors. Additionally, expression level and variance will inevitably affect the power to detect true GxC signals. We have included these analyses in the main text (page 7, paragraph 3).

3. A related question: it is mentioned that the quantile normalization of the expression data is quite harsh for lower expressed genes, as one would expect.
I would assume that the method performs best when the interaction context distribution is approximately normal. What happens if there are strong outliers, very tight clusters, etc? Should e.g. MOFA factors (or whatever is used instead) be normalized somehow?

We thank the reviewer for raising this point.

First, we note that in our applications the MOFA factors used to construct the cell context covariance are indeed standardised, such that each factor is centred at 0 and with standard deviation=1 to mitigate these effects. We now discuss alternative processing choices and provide more in-depth guidelines on data processing in **Methods** (page 15, paragraph 2). This information has also been included as part of the software web page (https://limix.github.io/CellRegMap) - see also response to comment 7 below.

Regarding the suitability of this preprocessing step especially in cases with extreme outliers, we agree that this strategy is conservative in nature and may result in loss in power. In general, we note that our “E” (or “C”) is meant to serve as a proxy for the actual causal cellular context and these estimates are bound by technology, the set of genes measured, etc. While conservative pre-processing may reduce the extent to which this proxy captures the true underlying structure, the power to detect interactions with rare cell states is intrinsically limited anyway. Hence, we feel this is an intrinsic limitation of any GxC analysis method. We have included this limitation in the discussion section (page 14, paragraph 1).

4. A minor point: "This analysis confirmed the expected difference in effect sizes (Fig. 5e,f), but also highlighted subtle differences in the cis eQTL mapping profile for each of these traits (Fig. 5d). Notably, the trait associated with the top quantile of increased allelic effects also yielded higher evidence for co-localization with the disease GWAS signal (Supplementary Fig. 5.2)." - I don't quite understand what the "subtle differences" refers to. Isn't the improved evidence for colocalization just an outcome of better power, with more variants having real signal with meaningful correlation? The patterns of CellRegMap top/bottom in SFig 5.2 don't really look qualitatively different to me (in terms of the association landscape or p-value correlations).

We thank the reviewer and have now rephrased the text (page 11, paragraph 3 (excluding figure captions)). In particular, we note that considering fewer cells (and fewer donors) in a specific stratum decreases our power to detect eQTL in general, because of the smaller sample size and the larger number of zeroes considered when assessing expression level. Despite this, a model that considers only the most relevant subset of cells as identified by CellRegMap does increase (slightly) the colocalization signal and the eQTL effect size (*i.e.*, panel f vs e) "quantitatively", if not qualitatively.

5. It would be good to mention that discovering the cell population where a given GWAS eQTL has its strongest effect does not necessarily mean that this is The Cell State that is driving the disease association. It could be that a genetic effect of smaller magnitude in a different cell type is what actually matters.

We agree with the reviewer that what matters is the improved colocalization signal (despite the lower power to detect the eQTL to begin with, see also our response to the previous comment) - we have revised the text to reflect this (page 11, paragraph 3).

6. Speed seems to be a limiting factor ,here. I'd like to see an expansion of SFig 1.1 to more realistic sample sizes - QTL mapping with 25 individuals is not a great idea to begin with, and even the real data used here has much higher sample sizes than presented here.

First, we have now more clearly specified the computational complexity of the model (page 4, paragraph 3 and **Methods**, page 18, "Computational complexity"). Briefly, the runtime complexity of CellRegMap is O(n), where n is the minimum between i) total number of cells and ii) product of number of individuals and number of cellular contexts considered (**Supplementary Fig. 1**). We also extended the simulation studies to more realistic numbers of individuals (and more realistic parameters in general, see response to reviewer3, comment 1, and **Methods**, pages 16-17, **Fig. EV1**, **Supplementary Figs. 1,3**).

Finally, while it is certainly true that runtime complexity can be a limiting factor, CellRegMap is primarily aimed at mapping GxC effects for a set of known eQTL, rather than identifying genetic associations *de novo*. In order to offer a comprehensive end-to-end workflow, we have extended our framework and software, which now includes a much more efficient association test that corresponds to the null model of CellRegMap (CellRegMap-association test; **Supplementary Fig. 1**). This test can be used to screen for variants with evidence for association signals, which in general can serve as an appropriate selection step for the discovery of GxC effects (see also response to reviewer3, comment 4 for further details).

7. I would encourage the authors to add more material in the CellRegMap website to provide more guidance on the important practicalities regarding thresholds, pseudocell calculation (when is it needed exactly?), normalization, multiple testing correction, etc.

We thank the reviewer for this point and have now added more information in the webpage (https://limix.github.io/CellRegMap), while also having streamlined and added features to the package itself (see reviewer3, comment 4 below for more detail).

In particular, the webpage now includes better guidelines regarding i) installation and usage of CellRegMap, ii) practical pre- and post-processing steps. It also includes more tutorials and info on the relationship of CellRegMap to the related models StructLMM (Moore et al. 2019) and LIMIX (Lippert et al., n.d.).

See also response to comment 3 above.

Reviewer #3:

Summary:
The authors create a tool to estimate the impact of genotype on single cell RNA profiles, called CellRegMap. This tool is based on a linear model that takes into account: 1) persistent genetic effects across all cells; 2) cellular context as defined from a co-variance structure obtained from e.g. PCA or MOFA; 3) interaction between genotype and cellular context (which also includes environmental effects on the individuals); 4) relatedness of cells profiled from the same individual.

This tool has the following advantages compared to existing approaches:
• Cellular contexts (cell types / states / differentiation pseudotimes) do not have to be discretized, which more accurately captures variation of gene expression
• Interactions between cellular contexts and genotype are tested, which allows for the discovery of e.g. loci with different impacts on expression in different cell types
• Different cellular contexts are modelled together, which allows to identify shared effects

In general, the paper makes a good impression; most parts of it are very clearly written (exception see below) and the method as such is very solid. Needless to say, a (good) method to detect eQTL in single cell data is really needed. I think the way to take subtle cell subtypes into account is elegant. Further, the code on GitHub is very well structured and documented. Further, the testing was done on scRNA-seq data from two different platforms (Smart-seq2 & 10X).
While I have no concerns regarding the method as such, its validation needs to be improved (see below). Also, in their submission the authors restricted the application of CellRegMap to a priori known eQTL. There might be computational and statistical (multiple testing problem) reasons for such a restriction. However, the authors do not even attempt to discover new eQTL, although their method provides great promise to detect context-specific eQTL that might remain undetected in an unspecific we-gonna-merge-everything approach. Further, the authors need to characterize the genes that benefit from explicit modelling of the context. What makes them different? (see below)

This is a pure methods paper. I.e. the editor needs to consider if this submission fits the scope of MSB. However, I do see a need for such method.

We thank the reviewer for their valuable detailed feedback and their overall positive assessment of our work, and have addressed their comments below. In particular, we have 1) revised and extended our simulation strategy to demonstrate the performance of CellRegMap using more realistic settings, 2) implemented strategies to enable the *de novo*

discovery of eQTL prior to testing for GxC effects (while maintaining computational feasibility and speed) and 3) explored in more detail characteristics of genes that display GxC effects.

Major Points:

1. Simulations are based on the model underlying CellRegMap. Obviously, this will favor CellRegMap as the best method to detect the eQTL. This is kind of circular reasoning. I do appreciate that the simulations without interaction effects contribute, because they show that the method does not produce excessively many false positives. Further, those simulations do show that the method works under its assumptions. However, the simulations need to be more realistic. (1) Real genotypes should be used to capture realistic distributions of MAFs and LD. (2) Low read counts and dropouts should be simulated. scRNA-seq data suffers from many specific problems such as dependency on library size/capturing efficiency, etc. There are existing tools such as splatPop (http://www.bioconductor.org/packages/devel/bioc/vignettes/splatter/inst/doc/splatPop.html) that should be used. Even better would be a methods comparison on real data. This is of course much harder, since the ground truth is not known. One possible way would be to quantify how much the observed eQTL colocalize with relevant genes and regulatory loci from previous GWAS or eQTL studies, or based on annotation. The authors already touched on this concept in the final results paragraphs.

We thank the reviewer for raising this important point, and have now improved our simulation strategy in multiple ways. First, instead of relying on the CellRegMap model itself to simulate context-specific genetic effects, we now simulate GxC interactions using a semi-synthetic procedure, which is based on empirically observed genotypes, as well as background gene expression profiles and cellular contexts derived from the endoderm differentiation data (see **Methods**, pages 16-17).

First, to avoid confounding with true existing *cis* eQTL in this dataset, we swap the genotype and gene labels, *i.e.,* eQTL and GxC effects are generated using genotypes from a gene located on another chromosome. Next, to simulate context-specific genetic effects, we draw samples from a conventional linear interaction model with a Poisson noise model:

$$y \sim Poisson(\lambda), \lambda = exp\,(y_{base} + \sum_{i=1}^{k} g \odot c_i {\beta_i}^{G\times C} + g\beta^G),$$

where

- y_{base} is the log-transformed observed (base line) gene expression profile for a given gene in the reference dataset,
- g is the SNP genotype from the reference dataset (non-match permuted gene),
- c_i denotes the *i*-th context variable (MOFA factor),
- ${\beta_i}^{G\times C}$ is the interaction effect size for context *i,* and,
- β^G is the effect size of the persistent genetic effect

(for more details, please see **Methods**, pages 16-18). The advantage of this strategy is that possible confounding factors such as read count distribution (dropout, overdispersion), batch effects or context-specific expression variation are present in the observed expression counts y_{base} and do not need to be simulated using a parametric / model-based approach. Furthermore, it allows us to simulate continuous interaction effects, which to the best of our knowledge is currently not possible using splatPop or related simulation frameworks (which only allow to model effects that are specific to one or more discrete cell group).

Our power and calibration assessments under this revised simulation procedure, largely follow the trends previously described in the original submission (revised **Fig. 2, Fig. EV1**).

Finally, we also use the simulated dataset to assess the calibration of CellRegMap as well as other methods (revised **Fig. 2**, **Fig. EV1**). Reassuringly, CellRegMap and also the corresponding association test (see response to comment 4 below) are calibrated.

2. The authors state that there are already established approaches for multi-tissue eQTL analysis. I don't know whether specific methods for single-cell-eQTL analysis exist already. Either way, I would like to see how their approach fares compared to these "established" methods, both in the simulation and the real data. The only comparison they performed was on the simulated methods, where they also applied a simpler model that could not handle multiple genetic contexts as well as their CellRegMap (Figure 2). This is not surprising, because this simpler model only allowed for a single context by design. So this comparison is not really a fair one, especially because the simulated data were created according to the assumed model of CellRegMap (and with only 50 cells).

First, the reviewer makes an important point about the extent to which these “established” methods are applicable in this setting. Specifically, while multi-tissue eQTL methods (for bulk expression data) do exist [references 5-13 in the main text], current methods:

1. rely on discretization of cells into distinct cell types, which CellRegMap explicitly aims to avoid,
2. do not explicitly test for “interaction effects”, but rather test for genetic effects that are present in one or multiple of the defined cell types, and
3. do not effectively account for repeated or related samples, thus are not well suited for single-cell data, where multiple cells are assayed for each individual (see main text **Figure 2**).

We have revised the main text to clarify these distinctions (page 2, paragraph 2). In particular points 2&3 imply that we can neither run these methods out of the box, nor can their results be directly compared to those obtained from CellRegMap.

Having said this, we agree that a comparison to a “discretization-based” strategy would certainly be a useful addition. To address this, we now compare continuous cell state representations to using discrete cell states, which can also be encoded in the cell context covariance matrix. Both simulated data benchmarks and a comparison on real data (see our answer to point 5 from the same reviewer below and **Supplementary Fig. 2**) demonstrate the expected benefits of CellRegMap by avoiding the discretization step.

Moreover, as the reviewer points out, we compare CellRegMap to a baseline approach that considers each context individually using a linear fixed effect model, and assesses the evidence for context-specific genetic effects using multiple likelihood ratio tests (SingleEnv-LRT), followed by multiple testing correction across contexts; a strategy that has been considered elsewhere (*e.g.,* (Zhernakova et al. 2017; van der Wijst et al. 2018)). Indeed, one could argue that a multiple degrees of freedom fixed effect test that jointly tests for an interaction effect with all contexts would be a more natural comparison partner. We have implemented this approach using a multiple degree of freedom likelihood ratio test (MultiEnv-LRT). However, as has been noted elsewhere (*e.g.,* (Moore et al. 2019)), such a method is

not very well calibrated, especially for large numbers of tested contexts relative to the sample size. We have confirmed that this lack of robust calibration of MultiEnv-LRT also applies to the single-cell setting (**Fig. EV1),** which is a major limitation of this baseline approach. As the method is not calibrated, we have not considered this strategy in the power assessment.

3. The current approach assumes a Gaussian normal distribution although it is community standard to assume a Negative Binomial and (sometimes) zero inflation. This concern is addressed in the Discussion section, but it would be relevant to show how applicable this model is to highly expressed vs lowly expressed target genes with a clear performance comparison in real scRNA-seq data. Supplementary Figure 2.2 shows that the power is almost twice as high when assuming a Negative Binomial as opposed to a Gaussian (compare panels b and d "Number of contexts with GxC"). Why do the authors not assume NB by default? How does the power depend on the expression level? It is likely that the Gaussian will fail especially for lowly expressed genes.

We agree with the reviewer that a Gaussian likelihood model is in general not optimal for count data. On the other hand, the explicit implementation of a non-Gaussian likelihood in a Generalised Linear Mixed Model (GLMM) setting is computational very prohibitive (Knudson et al. 2021; Bolker et al. 2009) and in particular in the context of genetics applications is hence frequently avoided. For these reasons, we have opted for retaining the assumption of Gaussian-distributed data and account for the distributional properties of the scRNA-seq data using appropriate data processing steps. Specifically, in order to improve model-fit we quantile-normalised the single-cell expression phenotypes and standardise the cell contexts (*i.e.,* in our application, MOFA factors, see also response to reviewer 1, comment 3). Nevertheless, our new simulation procedure is designed to produce read count distributions more similar to those found in real data and no longer assumes Gaussian noise. In this context, we also assess the dependency between mean/variance and the power to detect GxC effects, finding an expected association with expression level (see **Supplementary Fig. 3** in response to reviewer 1, comment 2).

Based on these data, we can conclude that false positive associations caused by the likelihood mismatch are not to be expected. It is of course plausible that in particular for lowly expressed genes, there would be power benefits when explicitly modelling the Poisson component in the likelihood. To some extent this effect is also mitigated by the pseudo cell approach, which we recommend for high-throughput sparse droplet-based methods. We provide guidance on how and when to use it in **Methods** (page 20). Further refinements are an area of future work, which we mention in the discussion section (page 13 paragraph 4 and page 14, paragraph 1).

4. The way CellRegMap is used so far is only re-analyzing eQTL that were previously detected using traditional analyses (see above). Why is that? The runtimes don't seem so terribly bad that running it in an unbiased way would be completely prohibitive. It should be tested at least for some traits. Furthermore, the authors only state that "the computational complexity of CellRegMap scales linearly with the number of cells". A little more detail about computation times in the main text would be helpful to give readers an idea of which application would be feasible. Finally, I think the text in the Introduction and Abstract should be adapted to honestly reflect the fact that this method is not feasible for de novo eQTL detection in larger datasets. Since the methods used for locus-gene pair preselection do not

take diverse contexts into account, one would still "fail to detect changes in allelic regulation across subtle cell subtypes" if the effect are not also visible on the global scale.

First of all, the reviewer is correct that the primary application of CellRegMap is the (re)analysis of known eQTL variants to assess the evidence for GxC interaction effects. It is also correct that the model can in principle also be used for discovery, although the computational cost of the model can be prohibitive for this task (see also revised **Supplementary Fig. 1**). The reanalysis of known eQTL variants makes the assumption that evidence from a conventional association test can be used to prioritise interaction tests, an assumption that is also commonly employed in the analysis of genotype-environment interactions (GxE) in organismal traits (Moore et al. 2019; Zhernakova et al. 2017). Such a filtering step implicitly assumes that the majority of GxC effects still manifest as (significant) associations that can be detected using a conventional test.

Having said this, we agree with the referee that the selection of eQTL variants and the possibility to conduct unbiased GxC analyses deserve more attention and the basis of using an association test as a filtering step to detect GxC effects needs to be established in this context. To address these points, we now provide additional analyses and we have extended the software so that it can be used in an end-to-end manner in case there exist no suitable set of reference eQTL variants.

In particular, when using CellRegMap for discovery, we recommend employing a two-stage testing approach, combining (i) variant discovery and (ii) GxC interaction test (CellRegMap). To facilitate this workflow, the CellRegMap software package now comes with an “association test” that corresponds to the null model of CellRegMap, *i.e.,* assessing a persistent effect with both additive and multiplicative background effects due to cellular environment (see **Methods**, page 17). Critically, this test is computationally much more efficient than the interaction test (c.f. **Supplementary Fig. 1;** see also **Methods,** “Computational Complexity”), while retaining calibration (**Fig. EV1**). Applied to the endoderm differentiation dataset considered in the paper (Cuomo et al. 2020), this workflow yields near-identical results compared to the set of “published” eQTL variants, which we consider in the application of CellRegMap described in the main text (**Supplementary Fig. 4**). Beyond the analysis of lead eQTL variants, this analysis naturally generalises to considering a larger set of variants, *i.e.,* by relaxing the filtering criterion from the association test, variants with weaker evidence for additive effects can be considered for GxC analysis. By varying the threshold for the association test, sub-threshold SNP-gene pairs may be rescued that may still display GxC. As an illustration, we considered varying significant thresholds for the association signal to test for GxC for a subset of genes (n=643 expressed genes on chromosomes 20, 21 and 22, see also **Methods**, page 21). These results confirm the expected relationship between associations and GxC, thus justifying usage of the association signal as independent filter (**Supplementary Fig. 11a**). Having said that, there will be instances where a true GxC effect can be entirely missed when prioritising variants based on associations, and we agree these could be of interest. For example, in the analysis above there are 33 “novel” GxC effects without any additive effect (P_association>0.05). An example of this is shown in **Supplementary Fig. 11b**. We discuss this now in the paper (page 13, paragraph 1) and provide guidance on pre-selection of variants (page 16, paragraph 2).

Whether the method should be used in a discovery mode or as interpretation tool for known variants of interest (*e.g.,* eQTL from other single-cell or bulk studies, GWAS hits for relevant traits) will depend on the specific application. See also usage page on the website: https://limix.github.io/CellRegMap/usage.html.

Finally, we have revised both the introduction (page 2, paragraph 3) and the abstract (page 1) to describe the interpretation of known variants as the primary used case of CellRegMap.

5. Better show with real data the benefit of using a continuous context as compared to discretizing the context. Do both on the same real data and compare the results. How much do you gain? Also: what about the reverse? What if the cell types are actually discrete, but the context is modelled as a continuous (linear) variable. What would you lose?

We have now run CellRegMap on the neuronal development data ((Jerber et al. 2021)) and considered an increasing number of discrete environments (3, 9 and to 18 cell clusters) as opposed to using the MOFA factors as considered in the main analysis. As expected, the results from this alternative analysis recover the strongest GxC effects identified using continuous cell states, however the continuous analysis remains much better powered even when considering a very large number of discrete clusters (**Supplementary Fig. 2d-f**). This suggests that CellRegMap allows for the identification of more fine-grained effects, which would be missed by an approach that relies on discretization of the data.

Additionally, we considered the same analysis using simulated data (derived from empirical parameters obtained from the endoderm differentiation data (Cuomo et al. 2020) as described above). Context-specific effects were simulated for the leading MOFA factors and we compared the continuous CellRegMap model (based on MOFA factors) to a version of CellRegMap testing for genetic effects across using discrete cell clusters (**Supplementary Fig. 2a-c)**. As with real data, the power was slightly improved when considering a higher number of discrete clusters (12, 24, see **Methods**), but performance remained remarkably worse as compared to using continuous contexts (**Supplementary Fig. 2)**.

Nevertheless, it is possible to account for model and account for discrete cell states in the cell context covariance if this reflects the underlying biology, e.g. to detect changes between sexes, or between cases and controls. See also response to comment 2 above.

6. Which genes benefit most from modelling GxC interactions? There is one simple explanation: a gene is expressed in one context, but not in the other. In such a case it would be impossible to detect an eQTL in the context where the gene is not expressed. That example would represent a bona fide context-specific eQTL and the explanation would be really simple. How many of the context-specific GxC eQTL are of this type? Note that this is related to the question of an unbiased de novo screen for GxC eQTL. (Without that you wouldn't know unbiased fractions of eQTL of certain types.)

For a detailed response to this point, see our Reviewer 1, comment 2 and **Supplementary Figures 3, 6** and **7**. Briefly, we observe low correlation between the dynamic profile of single-cell expression across contexts and that of GxC genetic effects, suggesting that these are not dominant effects. We also confirmed that we retain test calibration when considering genes whose expression varies along the contexts, ruling out false positive effects. Finally,

we do observe small power differences as a function of the mean and variance of gene expression, with a improvement in power for more highly expressed and less variable genes.

7. The impact of using other upstream analyses (PCA or ZINB-WaVe, instead of MOFA) on the model performance is not clear.

We thank the reviewer and now have, for a subset of eQTL (considering only chromosome 22, 88 eGenes and 121 eQTL from the original study (Cuomo et al. 2020)) run CellRegMap interaction test on the endoderm differentiation data using 1) principal component analysis (PCA), 2) linearly decoded variational autoencoder (LDVAE (Svensson et al. 2020)) and zero-inflated negative binomial-based wanted variational extraction (ZINB-WaVE (Risso et al. 2018)), finding overall good concordance (**Supplementary Fig. 5**). We are describing these results in the main text (page 7, paragraph 2).

Minor Points:

8. Figure 1e: make explicit that the color indicates allelic effects. This is particularly important to distinguish the meaning of color from panel c in the same figure.

We thank the reviewer for this very good point, a legend has been added appropriately.

9. Page 4: "GxC interactions are modelled as an element-wise product between the expanded genotype vector g at a given locus and a GxC effect size vector beta_GxC ..." What does the index [1 ... N] in this equation refer to? Is this really an iteration over all cells? I thought iterating over the contexts would make more sense. But the first sentence in the paragraph suggests that the index refers to cells.

The index refers indeed to the cells. A cell-level effect size is estimated here (for a given SNP-gene pair, across all contexts tested, see **Methods**, page 14 and **Supplementary Methods**).

10. Figure 2d: Why does SingleEnv-Int still perform better when there are two nonzero GxC contributions? What if you have only one C with a real effect? Would CellRegMap overfit, i.e. would you get more false positives?

No, CellRegMap will retain calibration in this scenario. However, the single-environment model will be better powered to detect the effects when only one or two contexts have a real effect, the weakest of which will be missed by CellRegMap. CellRegMap is preferable in the case of effects driven by multiple contexts (>5, see main **Figure 2**).

11. Page 7: "The model with 10 components yielded a substantially larger number GxC effects (322 versus 183, FDR<5%;" Did the FDR estimation account for the fact that the model with 10 components is testing ten times more hypotheses?

No, because the hypothesis is just one, is beta_GxC different from 0 or no, in both cases.

12. Figure 4: Why was t-SNE used here for the visualization instead of MOFA?

We note that the tSNE representation here is calculated from all of the MOFA factors. While for the endoderm differentiation data (Figure 3) we show individual MOFA factors to provide more characterisation (particularly to illustrate that there were more axes of variation beyond the most obvious differentiation time), here we wanted to offer a global representation of all factors at once, largely to illustrate their ability to recapitulate the three main cell populations present in this dataset, and our ability to detect genetic effects at the level of sub-populations of cells.

13. Page 9: "We tested for GxC effects at 1,374 SNP-gene pairs identified as eQTL in at least one of the three discrete cell populations in the primary analysis of the data ..." This entire paragraph is very difficult to read. Don't use fluffy terms like 'landscape of GxC effects'. Be explicit about what you are doing. Same with 'allelic effect pattern'. What is that? What 'pattern'? Just say clearly what the distance measure is based on. Say explicitly what you are clustering. I think you are clustering eQTL, but that should be made explicit. Why do you correlate allelic effects with gene expression? Why can't you directly sort by allelic effect? Please, re-write this paragraph and let it be prove-read by some external colleague.

We have now rephrased this paragraph for clarity (page 9, paragraphs 2 and 3).

14. Figure 4 c-h: The ordering of panels/clusters seems quite arbitrary. Why don't you use the same ordering as in panel b (i.e. by size)? Could be fewer examples.

The order chosen here reflects the order in which these are introduced in the text, *i.e.,* first condition-specific effects (day 30, day 52, then day 52 rotenone-treated), then more granular effects, *i.e.,* effects specific to sub populations of cells.

15. Page 11: "CellRegMap allowed for fine-mapping a specific sub-populations ..." Don't use 'fine mapping' here, because it has a different meaning in statistical genetics.

The reviewer makes an important point, we have now rephrased (page 11, paragraph 3 (excluding figure captions)).

16. Following sentence: check spelling and grammar.

Fixed.

17. Figure 5b (caption): Make explicit that these are the top and bottom quantiles of the 52 day untreated cells only (as opposed to top and bottom from all cells shown in the plot).

Rephrased (page 12).

References

Bolker, Benjamin M., Mollie E. Brooks, Connie J. Clark, Shane W. Geange, John R. Poulsen, M. Henry H. Stevens, and Jada-Simone S. White. 2009. “Generalized Linear Mixed Models: A Practical Guide for Ecology and Evolution.” *Trends in Ecology & Evolution* 24 (3): 127–35.

Cuomo, Anna S. E., Daniel D. Seaton, Davis J. McCarthy, Iker Martinez, Marc Jan Bonder, Jose Garcia-Bernardo, Shradha Amatya, et al. 2020. “Single-Cell RNA-Sequencing of Differentiating iPS Cells Reveals Dynamic Genetic Effects on Gene Expression.” *Nature Communications* 11 (1): 810.

Jerber, Julie, Daniel D. Seaton, Anna S. E. Cuomo, Natsuhiko Kumasaka, James Haldane, Juliette Steer, Minal Patel, et al. 2021. “Population-Scale Single-Cell RNA-Seq Profiling across Dopaminergic Neuron Differentiation.” *Nature Genetics* 53 (3): 304–12.

Knudson, Christina, Sydney Benson, Charles Geyer, and Galin Jones. 2021. “Likelihood□based Inference for Generalized Linear Mixed Models: Inference with the R Package Glmm.” *Stat*. https://doi.org/10.1002/sta4.339.

Lippert, Christoph, Franceso Paolo Casale, Barbara Rakitsch, and Oliver Stegle. n.d. “LIMIX: Genetic Analysis of Multiple Traits.” https://doi.org/10.1101/003905.

Moore, Rachel, Francesco Paolo Casale, Marc Jan Bonder, Danilo Horta, BIOS Consortium, Lude Franke, Inês Barroso, and Oliver Stegle. 2019. “A Linear Mixed-Model Approach to Study Multivariate Gene-Environment Interactions.” *Nature Genetics* 51 (1): 180–86.

Risso, Davide, Fanny Perraudeau, Svetlana Gribkova, Sandrine Dudoit, and Jean-Philippe Vert. 2018. “A General and Flexible Method for Signal Extraction from Single-Cell RNA-Seq Data.” *Nature Communications* 9 (1): 284.

Svensson, Valentine, Adam Gayoso, Nir Yosef, and Lior Pachter. 2020. “Interpretable Factor Models of Single-Cell RNA-Seq via Variational Autoencoders.” *Bioinformatics* 36 (11): 3418–21.

Wijst, Monique G. P. van der, Harm Brugge, Dylan H. de Vries, Patrick Deelen, Morris A. Swertz, LifeLines Cohort Study, BIOS Consortium, and Lude Franke. 2018. “Single-Cell RNA Sequencing Identifies Celltype-Specific Cis-eQTLs and Co-Expression QTLs.” *Nature Genetics* 50 (4): 493–97.

Zhernakova, Daria V., Patrick Deelen, Martijn Vermaat, Maarten van Iterson, Michiel van Galen, Wibowo Arindrarto, Peter van ’t Hof, et al. 2017. “Identification of Context-Dependent Expression Quantitative Trait Loci in Whole Blood.” *Nature Genetics* 49 (1): 139–45.

1st Revision - Editorial Decision — 8th Jun 2022

8th Jun 2022

Manuscript Number: MSB-2021-10663R
Title: CellRegMap: A statistical framework for mapping context-specific regulatory variants using scRNA-seq
Author: Anna Cuomo
Tobias Heinen
Danai Vagiaki
Danilo Horta
John Marioni
Oliver Stegle

Thank you for sending us your revised manuscript. We have now received the comments from both referees. As you will see below, the referees are satisfied with the modifications and think the study is now suitable for publication.

Before we can formally accept your manuscript, we would ask you to address the following issues:
1. Please address the minor comment of Referee #3.

On a more editorial level:

1. please provide up to 5 keywords and incorporate them into the main text.

2. Figures
- Please remove the main figures from the manuscript file and EV figures from the Appendix.
- Upload main and EV figures separately with individual production-quality figure files as .eps, .tif, and .jpg (one file per figure).
- Figure 5 A,b, and C are not called out. Please fix it.
- Please combine the legends for the main figures in the manuscript file in a section called main Figure Legends. Legends for EV figures should be placed after the main Figure Legends section.

3. Appendix
- Please update the nomenclature in Appendix and manuscript file to Appendix Figure S1, S2 etc.
- Please remove the EV figures from the Table of Content.
- Please note that this file will not be typeset or proofread.

4. The Supplementary Tables are rather large. Please turn them into EV Datasets using the nomenclature Dataset EV1, Dataset EV2, and so on. Remove their legends from the Appendix file and insert each legend into each corresponding dataset in a separate sheet. Please update all corresponding callouts in the manuscript and remove them from the Table of Content in the Appendix file.

5. We updated our journal's competing interests policy in January 2022 and request authors to consider both actual and perceived competing interests. Please review the policy https://www.embopress.org/competing-interests and add your competing interests if necessary.

Please add a disclosure statement using the heading "Disclosure statement and competing interests".

6. The references must be formatted according to the Molecular Systems Biology reference style. Please list up to 10 co-authors of a paper before adding et al. to the reference list. Citations should be listed in alphabetical order. Please remove DOI links for published articles.

7. Methods section:

- Please merge the Supplementary methods with the main Method section.
- Please use 'Structured Methods', our new Materials and Methods format. According to this format, the Material and Methods section should include a Reagents and Tools Table (listing key reagents, experimental models, software and relevant equipment and including their sources and relevant identifiers) followed by a Methods and Protocols section in which we encourage the authors to describe their methods using a step-by-step protocol format with bullet points, to facilitate the adoption of the methodologies across labs. More information on how to adhere to this format as well as downloadable templates (.doc or .xls) for the Reagents and Tools Table can be found in our author guidelines: <
https://www.embopress.org/page/journal/17444292/authorguide#researcharticleguide>. Several examples of Method papers with Structured Methods can be found here: https://www.embopress.org/doi/full/10.15252/msb.20199083;

https://www.embopress.org/doi/full/10.15252/msb.20188552; https://www.embopress.org/doi/full/10.15252/msb.20209701.

8. Data availability

- Please merge the Data and Code into a single section named Data Availability.
- Please note that the Data Availability Section is restricted to new primary data that are part of this study. Therefore, please remove the Zenodo dataset links. Instead, the datasets from References 1 and 3 can be cited with *data citations in the reference list*. Data citations in the article text are distinct from normal bibliographical citations and should directly link to the database records from which the data can be accessed. In the main text, data citations are formatted as follows: "Data ref: Smith et al, 2001". In the Reference list, data citations must be labeled with "[DATASET]". A data reference must provide the database name, accession number/identifiers, and a resolvable link to the landing page from which the data can be accessed at the end of the reference. Further instructions are available at .

9. Please provide a "standfirst text" summarizing the study in one or two sentences (approximately 250 characters, including space), three to four "bullet points" highlighting the main findings, and a "synopsis image" (550px width and 400-600px height, PNG format) to highlight the paper on our homepage.
Here are a couple of examples:
https://www.embopress.org/doi/10.15252/msb.20199356
https://www.embopress.org/doi/10.15252/msb.20209475
https://www.embopress.org/doi/10.15252/msb.209495

10. Our data editors have seen the manuscript and made some comments and suggestions that need to be addressed (see attached file). Please send back a revised version (in track change mode), as we will need to go through the changes.

Click on the link below to submit your revised paper.

https://msb.msubmit.net/cgi-bin/main.plex

As a matter of course, please make sure that you have correctly followed the instructions for authors as given on the submission website.

Thank you for submitting this paper to Molecular Systems Biology.

Kind regards,

Jingyi

Jingyi Hou
Editor
Molecular Systems Biology

Reviewer #1:

The authors have done comprehensive revisions and addressed all my comments. The manuscript looks great and I have no further concerns.

Reviewer #3:

The authors have fully addressed my concerns. In particular I appreciate the more realistic simulations, the more detailed description of the methods and the clear advice on how to use CellRegMap.

Congratulations!

Just one very minor point:

In the section "The CellRegMap tests and downstream analysis" the beta for the interaction is sometimes typeset with GxC as a superscript and sometimes as a subscript. (\beta^{GxC} versus \beta_{GxC}). In the Poisson on page 17 this seems necessary, because beta is getting the index i. However, this switching between superscript and subscript is a bit confusing or at least distracting. E.g. on page 16 it is a superscript in the sentence before the equation, in which it is shown as a subscript. Maybe there is some meaning behind this that I am not getting. Otherwise one could maybe use superscripts throughout.

The authors performed the requested editorial changes.

1st July 2022
Manuscript number: MSB-2021-10663RR
Title: CellRegMap: A statistical framework for mapping context-specific regulatory variants using scRNA-seq

Dear Dr Stegle,

Thank you again for sending us your revised manuscript. We are now satisfied with the modifications made and I am pleased to inform you that your paper has been accepted for publication.

*** PLEASE NOTE *** As part of the EMBO Publications transparent editorial process initiative (see our Editorial at https://dx.doi.org/10.1038/msb.2010.72), Molecular Systems Biology publishes online a Review Process File with each accepted manuscripts. This file will be published in conjunction with your paper and will include the anonymous referee reports, your point- by-point response and all pertinent correspondence relating to the manuscript. If you do NOT want this File to be published, please inform the editorial office at msb@embo.org within 14 days upon receipt of the present letter.

Should you be planning a Press Release on your article, please get in contact with msb@wiley.com as early as possible, in order to coordinate publication and release dates.

LICENSE AND PAYMENT:
All articles published in Molecular Systems Biology are fully open access: immediately and freely available to read, download and share.

Molecular Systems Biology charges an article processing charge (APC) to cover the publication costs. You, as the corresponding author for this manuscript, should have already received a quote with the article processing fee separately. Please let us know in case this quote has not been received.

Once your article is at Wiley for editorial production you will receive an email from Wiley's Author Services system, which will ask you to log in and will present you with the publication license form for completion. Within the same system the publication fee can be paid by credit card, an invoice or pro forma can be requested.

Payment of the publication charge and the signed Open Access Agreement form must be received before the article can be published online.

Molecular Systems Biology articles are published under the Creative Commons licence CC BY, which facilitates the sharing of scientific information by reducing legal barriers, while mandating attribution of the source in accordance to standard scholarly practice.

Proofs will be forwarded to you within the next 2-3 weeks.

Thank you very much for submitting your work to Molecular Systems Biology.

Sincerely,
Jingyi

Jingyi Hou
Editor
Molecular Systems Biology

EMBO Press Author Checklist

Corresponding Author Name: Oliver Stegle, Anna Cuomo	
Journal Submitted to: Molecular Systems Biology	
Manuscript Number: MSB-2021-10663R	

USEFUL LINKS FOR COMPLETING THIS FORM
The EMBO Journal - Author Guidelines
EMBO Reports - Author Guidelines
Molecular Systems Biology - Author Guidelines
EMBO Molecular Medicine - Author Guidelines

Reporting Checklist for Life Science Articles (updated January 2022)
This checklist is adapted from Materials Design Analysis Reporting (MDAR) Checklist for Authors. MDAR establishes a minimum set of requirements in transparent reporting in the life sciences (see Statement of Task: 10.31222/osf.io/9sm4x). Please follow the journal's guidelines in preparing your manuscript.
Please note that a copy of this checklist will be published alongside your article.

Abridged guidelines for figures
1. Data
The data shown in figures should satisfy the following conditions:
- the data were obtained and processed according to the field's best practice and are presented to reflect the results of the experiments in an accurate and unbiased manner.
- ideally, figure panels should include only measurements that are directly comparable to each other and obtained with the same assay.
- plots include clearly labeled error bars for independent experiments and sample sizes. Unless justified, error bars should not be shown for technical replicates.
- if n<5, the individual data points from each experiment should be plotted. Any statistical test employed should be justified.
- Source Data should be included to report the data underlying figures according to the guidelines set out in the authorship guidelines on Data Presentation.

2. Captions
Each figure caption should contain the following information, for each panel where they are relevant:
- a specification of the experimental system investigated (eg cell line, species name).
- the assay(s) and method(s) used to carry out the reported observations and measurements.
- an explicit mention of the biological and chemical entity(ies) that are being measured.
- an explicit mention of the biological and chemical entity(ies) that are altered/varied/perturbed in a controlled manner.
- the exact sample size (n) for each experimental group/condition, given as a number, not a range;
- a description of the sample collection allowing the reader to understand whether the samples represent technical or biological replicates (including how many animals, litters, cultures, etc.).
- a statement of how many times the experiment shown was independently replicated in the laboratory.
- definitions of statistical methods and measures:
  - common tests, such as t-test (please specify whether paired vs. unpaired), simple χ2 tests, Wilcoxon and Mann-Whitney tests, can be unambiguously identified by name only, but more complex techniques should be described in the methods section;
  - are tests one-sided or two-sided?
  - are there adjustments for multiple comparisons?
  - exact statistical test results, e.g., P values = x but not P values < x;
  - definition of 'center values' as median or average;
  - definition of error bars as s.d. or s.e.m.

Please complete ALL of the questions below.
Select "Not Applicable" only when the requested information is not relevant for your study.

Materials

Newly Created Materials	Information included in the manuscript?	In which section is the information available? (Reagents and Tools Table, Materials and Methods, Figures, Data Availability Section)
New materials and reagents need to be available; do any restrictions apply?	Not Applicable	

Antibodies	Information included in the manuscript?	In which section is the information available? (Reagents and Tools Table, Materials and Methods, Figures, Data Availability Section)
For **antibodies** provide the following information:<br>- Commercial antibodies: RRID (if possible) or supplier name, catalogue number and or/clone number<br>- Non-commercial: RRID or citation	Not Applicable	

DNA and RNA sequences	Information included in the manuscript?	In which section is the information available? (Reagents and Tools Table, Materials and Methods, Figures, Data Availability Section)
**Short novel DNA or RNA including primers, probes**: provide the sequences.	Not Applicable	

Cell materials	Information included in the manuscript?	In which section is the information available? (Reagents and Tools Table, Materials and Methods, Figures, Data Availability Section)
**Cell lines:** Provide species information, strain. Provide accession number in repository **OR** supplier name, catalog number, clone number, and/**OR** RRID.	Not Applicable	
**Primary cultures:** Provide species, strain, sex of origin, genetic modification status.	Not Applicable	
Report if the cell lines were recently **authenticated** (e.g., by STR profiling) and tested for mycoplasma contamination.	Not Applicable	

Experimental animals	Information included in the manuscript?	In which section is the information available? (Reagents and Tools Table, Materials and Methods, Figures, Data Availability Section)
**Laboratory animals or Model organisms:** Provide species, strain, sex, age, genetic modification status. Provide accession number in repository **OR** supplier name, catalog number, clone number, **OR** RRID.	Not Applicable	
**Animal observed in or captured from the field:** Provide species, sex, and age where possible.	Not Applicable	
Please detail **housing and husbandry conditions**.	Not Applicable	

Plants and microbes	Information included in the manuscript?	In which section is the information available? (Reagents and Tools Table, Materials and Methods, Figures, Data Availability Section)
**Plants:** provide species and strain, ecotype and cultivar where relevant, unique accession number if available, and source (including location for collected wild specimens).	Not Applicable	
**Microbes:** provide species and strain, unique accession number if available, and source.	Not Applicable	

Human research participants	Information included in the manuscript?	In which section is the information available? (Reagents and Tools Table, Materials and Methods, Figures, Data Availability Section)
If collected and within the bounds of privacy constraints report on age, sex and gender or ethnicity for all study participants.	Not Applicable	

Core facilities	Information included in the manuscript?	In which section is the information available? (Reagents and Tools Table, Materials and Methods, Figures, Data Availability Section)
If your work benefited from core facilities, was their service mentioned in the acknowledgments section?	Not Applicable	

Design

Study protocol	Information included in the manuscript?	In which section is the information available? (Reagents and Tools Table, Materials and Methods, Figures, Data Availability Section)
If study protocol has been **pre-registered, provide DOI in the manuscript**. For clinical trials, provide the trial registration number **OR** cite DOI.	Not Applicable	
Report the **clinical trial registration number** (at ClinicalTrials.gov or equivalent), where applicable.	Not Applicable	

Laboratory protocol	Information included in the manuscript?	In which section is the information available? (Reagents and Tools Table, Materials and Methods, Figures, Data Availability Section)
Provide DOI OR other citation details if **external detailed step-by-step protocols** are available.	Not Applicable	

Experimental study design and statistics	Information included in the manuscript?	In which section is the information available? (Reagents and Tools Table, Materials and Methods, Figures, Data Availability Section)
Include a statement about **sample size** estimate even if no statistical methods were used.	Not Applicable	
Were any steps taken to minimize the effects of subjective bias when allocating animals/samples to treatment (e.g. **randomization procedure**)? If yes, have they been described?	Not Applicable	
Include a statement about **blinding** even if no blinding was done.	Not Applicable	
Describe **inclusion/exclusion criteria** if samples or animals were excluded from the analysis. Were the criteria pre-established?<br><br>If sample or data points were omitted from analysis, report if this was due to attrition or intentional exclusion and provide justification.	Not Applicable	
For every figure, are **statistical tests** justified as appropriate? Do the data meet the assumptions of the tests (e.g., normal distribution)? Describe any methods used to assess it. Is there an estimate of variation within each group of data? Is the variance similar between the groups that are being statistically compared?	Yes	Figure captions, Methods

Sample definition and in-laboratory replication	Information included in the manuscript?	In which section is the information available? (Reagents and Tools Table, Materials and Methods, Figures, Data Availability Section)
In the figure legends: state number of times the experiment was **replicated** in laboratory.	Not Applicable	
In the figure legends: define whether data describe **technical or biological replicates**.	Not Applicable	

Ethics

Ethics	Information included in the manuscript?	In which section is the information available? (Reagents and Tools Table, Materials and Methods, Figures, Data Availability Section)
Studies involving **human participants**: State details of **authority granting ethics approval** (IRB or equivalent committee(s), provide reference number for approval.	Not Applicable	
Studies involving **human participants**: Include a statement confirming that **informed consent** was obtained from all subjects and that the experiments conformed to the principles set out in the WMA Declaration of Helsinki and the Department of Health and Human Services Belmont Report.	Not Applicable	
Studies involving **human participants:** For publication of **patient photos**, include a statement confirming that consent to publish was obtained.	Not Applicable	
Studies involving experimental **animals**: State details of **authority granting ethics approva**l (IRB or equivalent committee(s), provide reference number for approval. Include a statement of compliance with ethical regulations.	Not Applicable	
Studies involving **specimen and field samples:** State if relevant **permits** obtained, provide details of authority approving study; if none were required, explain why.	Not Applicable	

Dual Use Research of Concern (DURC)	Information included in the manuscript?	In which section is the information available? (Reagents and Tools Table, Materials and Methods, Figures, Data Availability Section)
Could your study fall under dual use research restrictions? Please check biosecurity documents and list of **select agents and toxins** (CDC): https://www.selectagents.gov/sat/list.htm	Not Applicable	
If you used a select agent, is the security level of the lab appropriate and reported in the manuscript?	Not Applicable	
If a study is subject to dual use research of concern regulations, is the name of the **authority granting approval and reference number** for the regulatory approval provided in the manuscript?	Not Applicable	

Reporting

The MDAR framework recommends adoption of discipline-specific guidelines, established and endorsed through community initiatives. Journals have their own policy about requiring specific guidelines and recommendations to complement MDAR.

Adherence to community standards	Information included in the manuscript?	In which section is the information available? (Reagents and Tools Table, Materials and Methods, Figures, Data Availability Section)
State if relevant guidelines or checklists (e.g., **ICMJE, MIBBI, ARRIVE, PRISMA**) have been followed or provided.	Not Applicable	
For **tumor marker prognostic studies,** we recommend that you follow the **REMARK** reporting guidelines (see link list at top right). See author guidelines, under 'Reporting Guidelines'. Please confirm you have followed these guidelines.	Not Applicable	
For **phase II and III randomized controlled trials**, please refer to the **CONSORT** flow diagram (see link list at top right) and submit the CONSORT checklist (see link list at top right) with your submission. See author guidelines, under 'Reporting Guidelines'. Please confirm you have submitted this list.	Not Applicable	

Data Availability

Data availability	Information included in the manuscript?	In which section is the information available? (Reagents and Tools Table, Materials and Methods, Figures, Data Availability Section)
Have **primary datasets** been deposited according to the journal's guidelines (see 'Data Deposition' section) and the respective accession numbers provided in the Data Availability Section?	Not Applicable	
Were **human clinical and genomic datasets** deposited in a public access-controlled repository in accordance to ethical obligations to the patients and to the applicable consent agreement?	Not Applicable	
Are **computational models** that are central and integral to a study available without restrictions in a machine-readable form? Were the relevant accession numbers or links provided?	Yes	Code availability section, Supplementary Methods
If publicly available data were reused, provide the respective **data citations in the reference list**.	Yes	Data availability section, References
